# Py-CoMSIA: An Open-Source Implementation of Comparative Molecular Similarity Indices Analysis in Python

**DOI:** 10.3390/ph18030440

**Published:** 2025-03-20

**Authors:** Christopher L. Haga, Crystal N. Le, Xue D. Yang, Donald G. Phinney

**Affiliations:** Department of Molecular Medicine, The Herbert Wertheim UF Scripps Institute for Biomedical Innovation & Technology, Jupiter, FL 33458, USA; crystal.le@ufl.edu (C.N.L.); xyang2@ufl.edu (X.D.Y.); dphinney@ufl.edu (D.G.P.)

**Keywords:** CoMSIA, Python, 3D QSAR, grid-based QSAR

## Abstract

**Background/Objectives:** The progression of three-dimensional (3D) quantitative structure–activity relationship (QSAR) methodologies has significantly contributed to the advancement of medicinal chemistry and pharmaceutical discovery. Comparative Molecular Similarity Indices Analysis (CoMSIA) is a widely used 3D-QSAR technique. However, its reliance on discontinued proprietary software creates accessibility challenges. This work aims to develop an open-source Python library to address these limitations and broaden access to grid-based 3D-QSAR methods. **Methods:** Py-CoMSIA was developed in Python using RDKit and NumPy for calculations and PyVista for visualizations. **Results:** Py-CoMSIA provides a functional open-source alternative to proprietary CoMSIA software. It successfully implements the core CoMSIA algorithm and generates comparable similarity indices, as demonstrated by testing several benchmarking datasets including the original CoMSIA steroid dataset. **Conclusions:** The Py-CoMSIA library addresses the accessibility issues associated with proprietary 3D-QSAR software by providing an open-source Python implementation of CoMSIA. This tool broadens access to complex grid-based 3D-QSAR methodologies and offers a flexible platform for integrating advanced statistical and machine learning techniques.

## 1. Introduction

Comparative Molecular Similarity Indices Analysis (CoMSIA) is an advanced computational method that plays an important role in three-dimensional quantitative structure–activity relationship (3D-QSAR) modeling. CoMSIA is specifically designed to identify correlations between the structural properties of molecules and their corresponding biological activities, offering deep insights into key molecular features that drive interactions with biological targets. First introduced by Klebe and colleagues in the 1990s [1,2], CoMSIA emerged as a significant advancement over earlier methodologies such as Comparative Molecular Field Analysis (CoMFA) [3]. Building upon the foundational principles of CoMFA, CoMSIA addressed many of its predecessor’s shortcomings while introducing methodological enhancements that improved the robustness, accuracy, and interpretability of 3D-QSAR models. Over the past three decades, CoMSIA has established itself as a useful tool in pharmaceutical research, particularly in drug discovery and design, where understanding the intricate relationship between molecular structure and biological activity is critical for the development of potent and selective therapeutic agents.

Unlike traditional QSAR methods that rely on the two-dimensional representation of molecular structures [4,5], 3D grid-based QSAR techniques such as CoMSIA incorporate the three-dimensional nature of biological interactions. Molecular recognition, such as the binding of small molecules to target proteins, is inherently a spatial phenomenon, driven by steric interactions, electrostatic forces, hydrophobic effects, and hydrogen bonding. CoMSIA provides a means to systematically quantify and correlate these spatially dependent molecular properties with experimental data, such as ligand potencies or inhibition constants. By analyzing three-dimensional molecular similarity indices, this technique facilitates the precise mapping of molecular fields relevant to biological activity. These maps can be used to identify regions within a molecule that can be modified to enhance interaction with the target receptor, ultimately aiding in the rational design of optimized compounds.

Whereas CoMFA models are constructed using these two basic fields to compute 3D interaction maps, CoMSIA introduces a broader range of descriptors that encompass a more comprehensive spectrum of chemical and physicochemical interactions, incorporating five distinct types of molecular fields: steric, electrostatic, hydrophobic, hydrogen bond donor, and hydrogen bond acceptor fields [1]. These additional fields significantly enhance the method’s applicability by accounting for key interactions often overlooked by CoMFA, especially in cases where hydrophobic forces or hydrogen bonding dominate receptor-ligand recognition. By integrating these descriptors into its modeling framework, CoMSIA provides a more holistic view of the molecular determinants underlying biological activity.

CoMSIA uses a Gaussian function to calculate molecular similarity indices, representing a departure from the discrete interaction energy calculations traditionally employed in CoMFA [2]. In CoMFA, steric and electrostatic fields are derived using fixed interaction energy cutoffs based on molecular grids and probe atoms. While functional in theory, this approach often results in abrupt, discontinuous field distributions that poorly reflect the gradual nature of changes in molecular structure. CoMSIA, in contrast, avoids this limitation by generating continuous molecular similarity maps for all five field types, eliminating the sharp and non-physical cutoffs observed in CoMFA models. This ensures that small differences in molecular conformation or alignment translate into proportionately small differences in activity predictions, enhancing the interpretability of CoMSIA models, as derived maps are less prone to artifacts or unexpected changes. Additionally, the Gaussian-based calculation makes CoMSIA models less sensitive to factors that traditionally complicated CoMFA, such as molecular alignment, grid spacing, and probe atom selection [6].

Classically, CoMSIA analysis has been conducted using the Sybyl molecular modeling software platform developed by Tripos [7]. Sybyl provided the necessary computational framework to construct CoMSIA models, including tools for molecular alignment, grid creation, field calculation, and statistical modeling techniques such as partial least squares (PLS) regression. Throughout the 1990s and 2000s, Sybyl’s integration of CoMSIA made it the standard platform for researchers working in 3D-QSAR and molecular modeling. However, the discontinuation of Tripos’ Sybyl in the mid-2010s prompted a shift in the field, forcing researchers to transition to alternative software platforms capable of replicating CoMSIA’s functionality. Despite this, CoMSIA remains a core methodology in modern QSAR analysis, supported by closed-source, proprietary tools such as Schrödinger and Molecular Operating Environment (MOE) platforms that have adapted the functionalities originally popularized in Sybyl.

While the discontinuation of Sybyl presented challenges for some practitioners, the continued reliance on CoMSIA underscores its vital role in rational drug design. CoMSIA’s ability to provide detailed and interpretable structure–activity insights ensures its continued relevance as a critical tool in medicinal chemistry and computational drug discovery. Therefore, we sought to create an open-source, python-based implementation of CoMSIA (Py-CoMSIA) that attempts to replicate the entire CoMSIA pipeline. Herein, we describe the implementation of CoMSIA in python and compare our implementation with previous Sybyl-based analyses across multiple test sets of compounds.

## 2. Results

### 2.1. Steroid Benchmark Test Case

**Figure 1:**
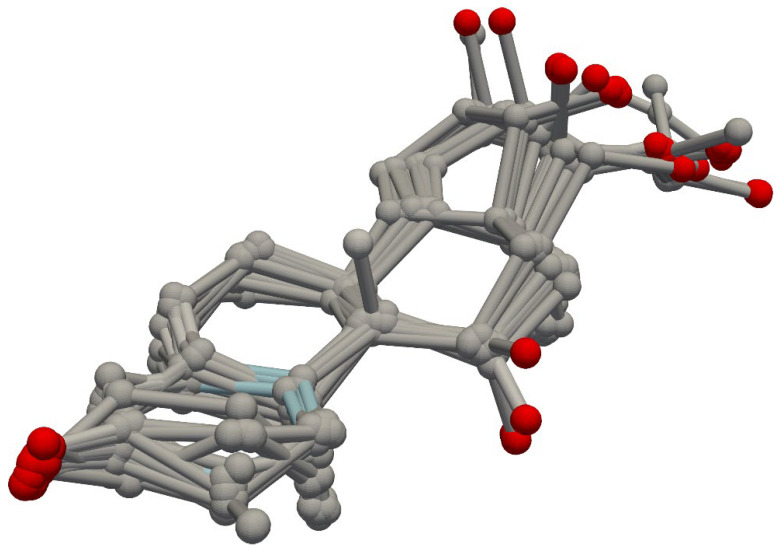
Alignment of steroid benchmark training set. Compounds in the steroid training set were overlayed using RDKit (release 2024.09.6) and rendered using PyVista (v. 0.44.2). Hydrogens have been removed from the molecules for clarity. Silver atoms represent carbons, and red represents oxygens.

**Figure 2:**
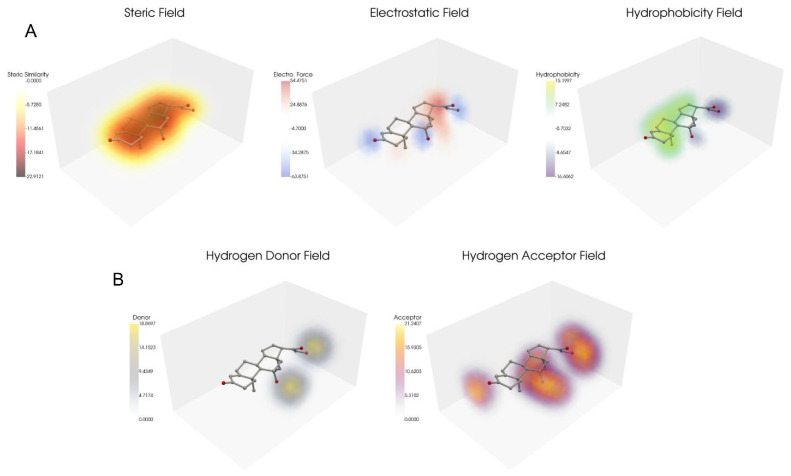
CoMSIA fields. (**A**) Steric, electrostatic, and hydrophobic (SEH) field renderings generated by Py-CoMSIA overlayed on top of aldosterone. (**B**) Additional hydrogen donor and acceptor fields overlayed on top of aldosterone. The gray box represents the grid used for calculating field points.

**Figure 3:**
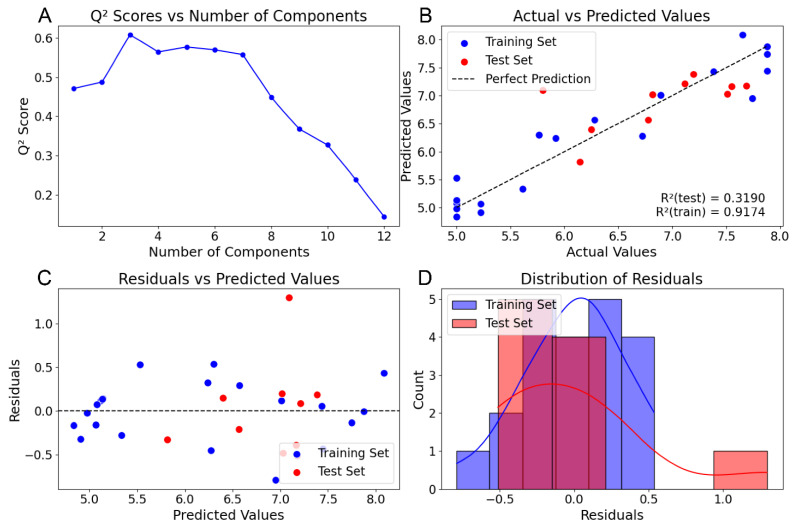
PLS regression analysis of SEH fields. (**A**) The q^2^ value for the indicated number of components from PLS regression with Leave One Out cross validation. (**B**) r^2^_train_ and r^2^_pred_ from final PLS regression using the optimal number of components. Blue dots indicate training set compounds. Red dots indicate test set compounds. (**C**) Residual values of training (blue) and test (red) compounds from the final PLS regression. (**D**) Distribution of residual values. A single outlier is detected (Compound 10) for the test set compounds. Blue = Training Set. Red = Test set.

To validate our open-source CoMSIA implementation, we conducted a comparative analysis of its results against previously published CoMSIA studies using Sybyl software. For initial validation, we selected the steroid benchmark dataset [8] that was used in the original CoMSIA analysis paper [2] for QSAR analysis with CoMSIA grid parameters. We analyzed this dataset using two parameter sets: the standard steric, electrostatic, and hydrophobic (SEH) parameters, and an extended set (SEHAD) including hydrogen bond donors and acceptors. Both sets were used for QSAR analysis. We used the Sybyl pre-aligned dataset [9] from Coats’ steroid benchmarking study, comprising 21 training and 10 test molecules, consistent with the original publication. A visual assessment of the alignment indicated close molecular grouping (Figure 1). Notably, the Coats’ dataset includes an extra test molecule, which we omitted from our analysis for consistency with prior studies. We used a grid spacing of 1, padding of 4, and attenuation of 0.3, consistent with the original research parameters. Fields were calculated and visualized using Py-CoMSIA’s visualization tool to confirm the Gaussian distribution of molecular properties for the SEH (Figure 2A) and hydrogen bond (Figure 2B) fields. Initial PLS regression with LOOCV was used to determine the optimal number of components for SEH and SEHAD field datasets, selecting for the lowest cross-validated q^2^ score. Figure 3A shows the q^2^ scores per component from the leave-one-out analysis. Following optimization, a final PLS regression model with the optimal number of components was trained using the training set, and the test set was used for prediction.

To evaluate Py-CoMSIA’s performance, we compared key metrics—q^2^, r^2^, S_PRESS_, S_train_, and field contributions—for SEH and SEHAD datasets against published Sybyl results. Table 1 shows that Py-CoMSIA (SEH) results closely matched Sybyl analyses, with minor variations likely from alignment differences due to the lack of original alignment data. Our analysis found three optimal components versus Sybyl’s four at the highest q^2^ value of 0.609 (Sybyl: 0.665) (Figure 3A). Despite a slightly lower r^2^ (0.937 vs. 0.917), our r^2^_pred_ (0.40) was comparable to Sybyl’s 0.318 (Figure 3B), indicating good predictive capability with acceptable residuals (Figure 3C,D). Importantly, like Sybyl, our analysis identified compound 10 as a predictive outlier, further validating Py-CoMSIA’s predictive performance.

**Figure 4:**
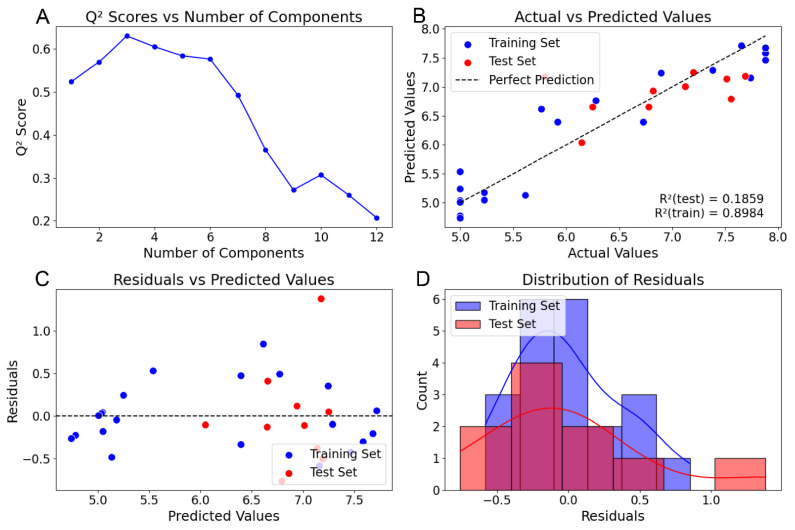
PLS regression analysis of SEHAD fields. (**A**) The q^2^ value for the indicated number of components from PLS Regression with LOOCV. (**B**) r^2^_train_ and r^2^_pred_ from final PLS regression using the optimal number of components. Blue dots indicate training set compounds. Red dots indicate test set compounds. (**C**) Residual values of training (blue) and test (red) compounds from the final PLS regression. (**D**) Distribution of residual values. Blue = Training Set. Red = Test Set.

Although no direct comparative analysis utilizing Sybyl with all fields (SEHAD) for this specific compound set is available in the literature, we incorporated a SEHAD analysis to showcase the comprehensive field handling capabilities of our Py-CoMSIA implementation. As presented in Table 1, the model incorporating all five fields (SEHAD) demonstrates a somewhat reduced overall predictive capacity compared to the models utilizing only the SEH subset. Nevertheless, the performance metrics obtained for the SEHAD model remain within a statistically acceptable performance envelope for CoMSIA-based QSAR analyses, indicating functional utility. Consistent with the SEH analysis, cross-validation of the SEHAD model identified an optimal component number of 3 (Figure 4A). However, the SEHAD model exhibited a demonstrably lower predictive r^2^ (0.186) compared to the SEH model (0.319) (Figure 4B) and displayed a broader and more dispersed distribution of prediction residuals (Figure 4C,D), suggesting a less robust model. Table 2 provides a comprehensive compilation of residual values for both the SEH and SEHAD analyses alongside those reported in the original steroid benchmarking study, enabling a detailed side-by-side comparison. Notably, despite the aforementioned differences in individual residual directionality, the mean and standard deviation of the residuals calculated for both our training and test sets in both SEH and SEHAD analyses are statistically comparable to those reported in the original publication. This parity in overall error magnitude suggests that while the specific predictions may exhibit some variation, the overall predictive accuracy and reliability of our Py-CoMSIA implementation remained on par with the original Sybyl-based analyses, further validating its applicability and performance for CoMSIA modeling.

### 2.2. Steroid Dataset Contour Plots

**Figure 5:**
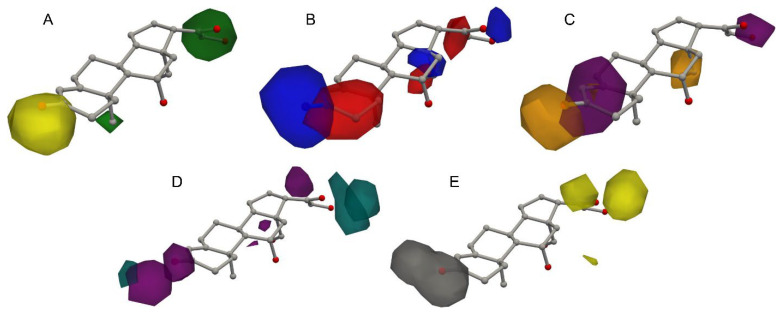
Contour plots of partial least squares (PLS) regression coefficients, illustrating regions of favorable and unfavorable interactions for various molecular properties. (**A**) Steric: yellow (favorable), green (unfavorable). (**B**) Electrostatic: red (favorable), blue (unfavorable). (**C**) Hydrophobic: orange (favorable), purple (unfavorable). (**D**) Hydrogen bond acceptor: teal (favorable), purple (unfavorable). (**E**) Hydrogen bond donor: yellow (favorable), gray (unfavorable). The molecule overlayed is aldosterone. Top 5% significant coefficients were used for the analysis.

A significant advantage inherent in grid-based QSAR methodologies employing PLS regression lies in their ability to generate informative contour plots [10]. These visualizations delineate specific spatial regions surrounding the investigated molecules that are deemed critical for modulating biological activity. Such plots are invaluable tools in rational drug design, as they provide a visual guide for strategically modifying molecular structures to potentially enhance activity profiles. Py-CoMSIA inherently incorporates the functionality to generate these contour plots by leveraging the PLS regression coefficients calculated for each grid point. A user-defined cutoff value is then applied to these coefficients, effectively thresholding the data to highlight and delineate regions in the molecular space that exhibit the most substantial influence on the predicted activity. Figure 5 illustrates the generated contour plots for the steric, electrostatic, and hydrophobic fields (Figure 5A, Figure 5B and Figure 5C, respectively), superimposed onto the aldosterone molecule. These plots effectively pinpoint specific regions around the molecule where alterations to these physicochemical properties are predicted to result in enhanced binding affinity. Similarly, Figure 5D,E presents the contour plots corresponding to the additional hydrogen bond acceptor and donor fields. These plots guide the rational modification of the molecule by highlighting areas where the introduction or modification of hydrogen bond functionalities is anticipated to positively impact the molecule’s biological activity.

### 2.3. Additional Benchmark Datasets

To further benchmark the performance and generalizability of Py-CoMSIA, we extended our analysis to six additional, diverse datasets, selected based on the availability of pre-existing, Sybyl-generated 3D molecular structures and prior, published CoMSIA analyses for direct performance comparison. The datasets chosen for this expanded benchmarking included well-characterized sets of angiotensin converting enzyme (ACE) inhibitors [11], acetylcholinesterase (AChE) inhibitors [11], anti-tuberculosis agents (ATA) [12], CCR5 receptor antagonists (CCR5) [13], thermolysin inhibitors (THERM) [11], and thrombin inhibitors (THR) [11]. Consistent with the steroid dataset analysis, we generated all five CoMSIA field types (steric, electrostatic, hydrophobic, hydrogen bond donor, and hydrogen bond acceptor) for each of these datasets, using a standardized grid spacing of 1 and grid padding of 4. For each dataset, a leave-one-out cross-validation PLS regression was performed on the training set compounds to determine the optimal number of PLS components, and to calculate the cross-validated q^2^ and S_PRESS_ metrics, A final PLS regression model was constructed using the training set with the optimal number of components, and the corresponding test set compounds were used for external validation. Statistical parameters including r^2^_train_, r^2^_pred_, S_train_, and S_test_ were calculated for Py-CoMSIA and compared to previously published results. As summarized in Table 3, the performance metrics obtained with Py-CoMSIA were, for the majority of benchmark datasets, demonstrably on par with or even slightly exceeding those reported in prior publications utilizing Sybyl. The notable exception to this trend was the anti-tuberculosis agents (ATA) dataset. It is important to note that even in previously published studies, the ATA dataset has consistently exhibited suboptimal performance in 3D-QSAR modeling, failing to reach generally accepted predictive r^2^ thresholds, suggesting inherent limitations in the dataset itself rather than the analytical methodology. Comprehensive analytical results and complete statistical outputs for each of these benchmark datasets are available in the Supplementary Materials.

## 3. Discussion

The development of Py-CoMSIA directly addresses a gap in the landscape of computational drug discovery: the lack of a freely available, open-source implementation of the widely used CoMSIA QSAR method. For years, CoMSIA, and its predecessor CoMFA, have been powerful tools for QSAR modeling, enabling the prediction of the biological activity of molecules based on their 3D structural and physicochemical properties and the design of novel derivatives through contour plots produced by the application. However, access to these techniques has largely been confined to users of proprietary software packages, most prominently Sybyl. The discontinuation of the Sybyl software suite has increased the need for alternative, accessible implementations of established QSAR methods like CoMSIA. While a non-open source, web-based implementation of CoMFA has been made freely available [14], an open-source version of CoMSIA has yet to be created. Py-CoMSIA directly addresses these needs, offering the scientific community a validated open-source solution.

Py-CoMSIA can be extended beyond providing a functional reimplementation of CoMSIA. By choosing Python as the programming language, we have positioned Py-CoMSIA within an expansive ecosystem of scientific computing tools. Py-CoMSIA can be readily integrated into existing computational workflows, such as utilizing other Sci-Kit based machine learning algorithms, as well as extended to function with deep learning architectures such as PyTorch [15] and TensorFlow [16]. This inherent interoperability allows for the seamless combination of Py-CoMSIA with other open-source tools for molecular model interpretation. As an open-source project, Py-CoMSIA benefits from the inherent advantages of community-driven development.

The validation studies presented here demonstrate the accuracy and reliability of Py-CoMSIA. By benchmarking Py-CoMSIA against established Sybyl results across a range of datasets, we have shown that it delivers comparable and, in some cases, even improved predictive performance. Researchers in academic settings and resource-limited environments, who may have previously been constrained by the high cost of proprietary software, now have access to a powerful CoMSIA implementation without limitations. 

Py-CoMSIA represents a significant contribution to the open-source cheminformatics toolkit. By providing a validated, freely available, and readily extensible Python implementation of CoMSIA, we have addressed a critical need in the scientific community. Py-CoMSIA not only preserves access to a valuable QSAR methodology, but also fosters a more open, collaborative, and accessible future for computer-aided molecular design and discovery.

## 4. Methods

### 4.1. Software Packages

Py-CoMSIA was created in Python 3.11 [17] using the Visual Studio Code (v. 1.97) integrated development environment. The application uses RDKit (release 2024.09.6) [18] and NumPy (v. 2.2.4) [19] for molecular properties and data calculations. PyVista (v. 0.44.2) [20] is used for visualizations. Scikit Learn (v. 1.6.1) [21] is used for conducting Partial Least Squares Regression analysis.

### 4.2. Datasets

Coats’ steroid dataset was used for the steroid analysis [8,9]. All other pre-aligned datasets were obtained from Rango’s Py-CoMFA publication [14].

### 4.3. Molecular Alignment

CoMSIA is highly dependent on accurate molecular alignment. While our implementation can accept previously aligned molecules in the sdf file format with included activities, we also implemented a basic molecular alignment algorithm for previously unaligned molecules using the RDKit cheminformatics library. Our basic implementation accepts a list of molecules represented by their Simplified Molecular-Input Line-Entry System (SMILES) [22] strings as input and can be called independently if utilizing the program for third-party applications. SMILES strings are converted into RDKit molecule objects. The algorithm then identifies the Maximum Common Substructure (MCS) among all input molecules, which defines the shared core structure upon which the alignment is based.

The first molecule in the input list serves as the template molecule, with its 2D coordinates computed, and is subsequently embedded in 3D space using a random seed for reproducibility. The alignment process iteratively considers each molecule in the input list. For every molecule, substructure matching is performed to identify occurrences of the MCS within both the template and the current molecule. A coordinate map is constructed to link the atoms of the MCS in the current molecule to their corresponding atoms in the template. Energy minimization is performed iteratively until convergence, ensuring a physically plausible and energetically favorable conformation. Following energy minimization, the Root Mean Square Deviation (RMSD) is calculated for the core atoms between the template molecule and the aligned molecule.

### 4.4. Molecular Grid Generation

A crucial step in grid-based CoMSIA studies is the generation of a consistent three-dimensional Cartesian grid that encompasses all aligned molecules in the dataset. This grid serves as the framework upon which molecular fields are calculated and compared. In our pythonic version of CoMSIA, the grid is implemented utilizing the NumPy library. The process begins with a set of molecules that have been aligned using a suitable method. For each molecule, the three-dimensional coordinates of its atoms are extracted. These coordinates are then discretized by mapping them onto a regular grid. This discretization involves rounding each atomic coordinate to the nearest grid point, effectively “snapping” the atoms to the grid. This step ensures that all molecules, regardless of their initial positions, are represented consistently with respect to a common spatial reference frame.

To define the boundaries of the grid, the minimum and maximum coordinates of the snapped atoms are determined along each of the x, y, and z axes. Padding is added to these extreme values to create a buffer zone around the molecules. This padding is essential to ensure that no part of any molecule falls outside the grid’s boundaries, particularly during subsequent field calculations, preventing potential edge effects and ensuring accurate representation of the molecular properties.

The grid itself is defined by its spacing, dimensions, and origin. The spacing between grid points is a crucial parameter, determining the resolution at which molecular properties are evaluated. A finer grid spacing leads to higher resolution but also increases computational cost. The dimensions of the grid, i.e., the number of grid points along each axis, are calculated based on the range of atomic coordinates (including padding) and the chosen grid spacing. Specifically, the number of points in each direction is determined by dividing the total range of coordinates in that direction by the grid spacing and rounding up to the nearest integer to ensure complete coverage of the molecular ensemble. The origin of the grid is set to the minimum coordinate values after padding has been applied.

### 4.5. Steric, Electrostatic, Hydrophobic, Hydrogen Donor, and Acceptor Field Calculations

A critical component of 3D-QSAR studies, particularly those employing grid-based methods like CoMSIA, is the calculation of molecular fields. These fields represent the various physicochemical properties of the molecules under investigation and are essential for understanding their interactions with biological targets. This process involves calculating five distinct types of molecular fields: steric, electrostatic, hydrophobic, hydrogen bond donor, and hydrogen bond acceptor.

Steric interactions are modeled using a Gaussian function centered on each atom, weighted by the cube of its van der Waals radius. Electrostatic interactions are similarly represented through a Gaussian function, weighted by the Gasteiger partial charge [23] assigned to each atom. The hydrophobic field follows a parallel approach, employing a Gaussian function weighted by Crippen’s hydrophobicity contributions [24] for each atom, providing a quantitative measure of the hydrophobic character of the local molecular environment.

Specific SMARTS patterns are used to identify potential hydrogen bond donor and acceptor atoms within the molecule [25]. In line with the original CoMSIA field calculations, for each identified atom, a set of pseudoatoms are generated at a fixed distance (1.9 Å) from the original atom, representing probable hydrogen bond interaction points. These positions are selected to avoid steric clashes with other atoms in the molecule, thereby ensuring the physical plausibility of the generated interaction sites [26]. The strength of the hydrogen bond interaction at each grid point is then calculated by applying Gaussian functions centered on these pseudoatom positions. Gaussian functions are used for both donor and acceptor atoms; however, the placement of the Gaussians differs. For donor atoms, the Gaussian function is placed at the calculated position of the hydrogen atom involved in the hydrogen bond. For acceptor atoms, multiple Gaussian functions are placed along vectors determined by the atom’s hybridization, representing the directional nature of the acceptor interaction. The Gaussian function parameters, specifically the decay constant (α), determine the spatial extent and falloff of these interactions.

The result of this process is a set of five 3D grids for each molecule, with each grid representing one of the molecular fields. These grids provide a detailed spatial representation of the molecule’s physicochemical properties and form the basis for subsequent 3D-QSAR analysis, enabling the correlation of these properties with biological activity.

### 4.6. Data Processing

The fields are initially stored as separate arrays for each molecule and each field type. The application first combines the field data for the training and prediction sets, if a prediction set is provided, to ensure consistent feature scaling and filtering across all molecules. Although more common to CoMFA analysis, we have implemented column filtering to eliminate low variance data points across molecules based on the range of values observed for each grid point across the dataset. Grid points with a range below a specified threshold are considered to have low variance and are removed from the analysis. This filtering step can reduce noise and focus the analysis on the most informative regions of the molecular fields in certain analyses. By default, the application does not perform column filtering but this feature can be implemented via command line argument. Each field is then scaled to have zero mean and unit variance. This scaling is performed across all molecules for each field separately to prevent bias from fields with larger absolute values. The standard deviation of each scaled field is also calculated and stored for later use in contour plot generation.

Finally, the processed and scaled fields are combined into a single feature matrix (X). For each molecule, the scaled values for all kept grid points across all fields are concatenated to form a single feature vector. These feature vectors form the rows of the X matrix. The feature matrix is then mean-centered by subtracting the mean of each column. The activities for each molecule are also mean-centered by subtracting the mean of activities in preparation for Partial Least Squares regression analysis.

### 4.7. Partial Least Squares Regression

The Partial Least Squares (PLS) regression [27] is used for model optimization, fitting, prediction, and performance evaluation, and is implemented via Sci-Kit Learn’s built-in PLS regression algorithm. Typically, classic CoMFA and CoMSIA utilizes a modified version of PLS regression, SAMPLS, that computes a covariance matrix representing the covariance between molecules, reducing the dimensionality. While SAMPLS [8] was useful in past days of low-power computing, its advantages in the modern era in terms of speed improvements are minimal. Additionally, it has yet to be implemented in Python. Model optimization is handled by iteratively determining the optimal number of latent variables through Leave-One-Out Cross-Validation (LOOCV) [28]. For each component number, the method trains a PLS model on all but one molecule and predicts the activity of the left-out molecule. The difference between the predicted and actual activity is used to calculate the PRESS (Predicted Residual Sum of Squares) statistic. The q^2^ value, a measure of predictive ability, is then calculated from the PRESS. The optimal number of components is chosen as the one that maximizes the q^2^ value. The available data are then split into training and testing sets. The final PLS model is trained on the training set, and its predictive performance is evaluated on the test set. The method calculates and stores several key metrics, including the r^2^ score for both training and test sets, S_PRESS_ and S (standard error of regression) values, and the contribution fraction of each field to the model. If a prediction set is provided, the method also makes predictions for these unseen molecules.

### 4.8. Contour Plots

As with other CoMSIA implementations, contour plots can be visualized by employing the marching cubes algorithm for isosurface generation overlaid on top of a molecule. Significant data ranges, defined by percentile-based thresholds, are automatically computed to highlight high and low regions within the fields of the PLS regression coefficients. The coefficients are multiplied by the standard deviation of the corresponding scaled field. These regions are then visually represented using 3D isosurfaces, where high-value regions are color-coded differently from low-value regions to facilitate interpretation.

## Figures and Tables

**Table 1:** PLS regression analysis of the SEH and SEHAD fields for the steroid benchmark dataset.

	Published (SEH)	Py-CoMSIA (SEH)	Py-CoMSIA (SEHAD)
q^2^	0.665	0.609	0.630
S_PRESS_	0.759	0.718	0.698
r^2^	0.937	0.917	0.898
S	0.33	0.33	0.366
no. comp	4	3	3
Field Contributions			
steric	0.073	0.149	0.065
electrostatic	0.513	0.534	0.258
hydrophobic	0.415	0.316	0.154
hydrogen donor			0.274
hydrogen acceptor			0.248

**Table 2:** Comprehensive analysis of residuals for the steroid benchmarking dataset.

Steroid	pKi	CoMSIA (SEH Published)	Py-CoMSIA (SEH)	Py-CoMSIA (SEHAD)
**Training Set**				
aldosterone	6.28	0.07	0.29	0.49
androstanediol	5	−0.08	0.12	0.05
androstenediol	5	0.21	−0.17	−0.22
androstenedione	5.76	−0.43	0.54	0.85
androsterone	5.61	0.41	−0.27	−0.48
corticosterone	7.88	0.14	0.00	−0.30
cortisol	7.88	0.16	−0.43	−0.42
cortisone	6.89	−0.19	0.12	0.35
dehydroepiandrostrone	5	0.38	0.14	0.03
deoxycorticosterone	7.65	−0.04	0.43	0.07
deoxycortisol	7.88	0.08	−0.14	−0.20
dihydrotestosterone	5.92	−0.46	0.32	0.47
estradiol	5	−0.04	0.08	0.01
estriol	5	0.2	−0.03	−0.26
estrone	5	0.11	0.14	0.25
etiocholanolone	5.26	−0.36	−0.16	−0.05
pregnenolone	5.26	−0.59	−0.32	−0.18
hydroxypregnenlone	5	0.35	0.53	0.54
progesterone	7.38	−0.32	0.06	−0.09
hydroxyprog	7.74	0.04	−0.79	−0.58
testosterone	6.72	0.36	−0.45	−0.33
**Mean**	**6.15**	**0**	**0.00**	**0.00**
**Standard Deviation**	**1.17**	**0.29**	**0.33**	**0.37**
**Test Set**				
prednisolone	7.51	−0.11	−0.48	−0.37
cortisol-21-acetate	7.55	−0.13	−0.39	−0.76
4-pregnene-3,11,20-trione	6.78	0.26	−0.21	−0.13
epicorticosterone	7.2	−0.55	0.19	0.05
19-nortestosterone	6.14	0.3	−0.33	−0.10
16a,17-dihydroxy-4-pregnene-3,20-dione	6.25	−0.93	0.15	0.41
16a-methyl-4-pregnene-3,20-dione	7.12	−0.23	0.09	−0.11
19-norprogesterone	6.82	−0.08	0.20	0.12
11b,17,21-trihydroxy-2a-methyl-4-pregnen-3,20-dione	7.69	0.03	−0.51	−0.50
11b,17,21-trihydroxy-2a-methyl-9a-fluoro-4-pregnen-3,20-dione	5.8	−2.02	1.30	1.38
**Mean**	**6.89**	**−0.35**	**0.00**	**0.00**
**Standard Deviation**	**0.65**	**0.69**	**0.51**	**0.56**

**Table 3:** PLS regression results of a collection of benchmarking datasets. Field codes: S = steric, E = electrostatic, H = hydrophobic, A = hydrogen bond acceptor, D = hydrogen bond donor. N/A information not available in original study.

Dataset	Fields	r^2^_train_	r^2^_train_ Published	r^2^_test_	r^2^_test_ Published	q^2^	q^2^ Published	S_PRESS_	S_PRESS_ Published	S_train_	S_train_ Published	S_test_	S_test_ Published	No. Comps	No. Comps Published
ACE	SEH	0.76	0.73	0.57	0.49	0.65	0.65	1.38	1.36	1.14	1.15	1.38	1.48	3	2
AChE	SEHAD	0.82	0.86	0.57	0.44	0.43	0.49	0.91	0.89	0.51	0.45	0.84	0.98	6	4
ATA	SEHAD	0.74	0.86	−0.77	−0.41	0.29	0.46	1.10	N/A	0.65	N/A	1.28	N/A	5	6
CCR5	SEHAD	0.93	0.94	N/A	N/A	0.81	0.78	0.38	N/A	0.22	N/A	0.84	N/A	5	5
THER	SEAD	0.87	0.77	0.43	0.53	0.63	0.51	1.15	1.35	0.66	0.91	1.65	1.6	4	3
THR	SEHAD	0.84	0.89	0.51	0.63	0.66	0.72	0.55	0.56	0.38	0.32	0.77	0.69	3	4

## Data Availability

All code for Py-CoMSIA is available for download at https://github.com/clhaga/pycomsia/ (accessed on 31 January 2025).

## Supplementary Materials

The following supporting information can be downloaded at: https://www.mdpi.com/article/10.3390/ph18030440/s1, File S1. Additional benchmarking dataset analyses.
