# Supplementary figures and images for: Py-CoMSIA: An Open-Source Implementation of Comparative Molecular Similarity Indices Analysis in Python
Source: Pharmaceuticals (Basel). 2025 Mar 20;18(3):440. doi: 10.3390/ph18030440 (PMC11945924; doi:10.3390/ph18030440)

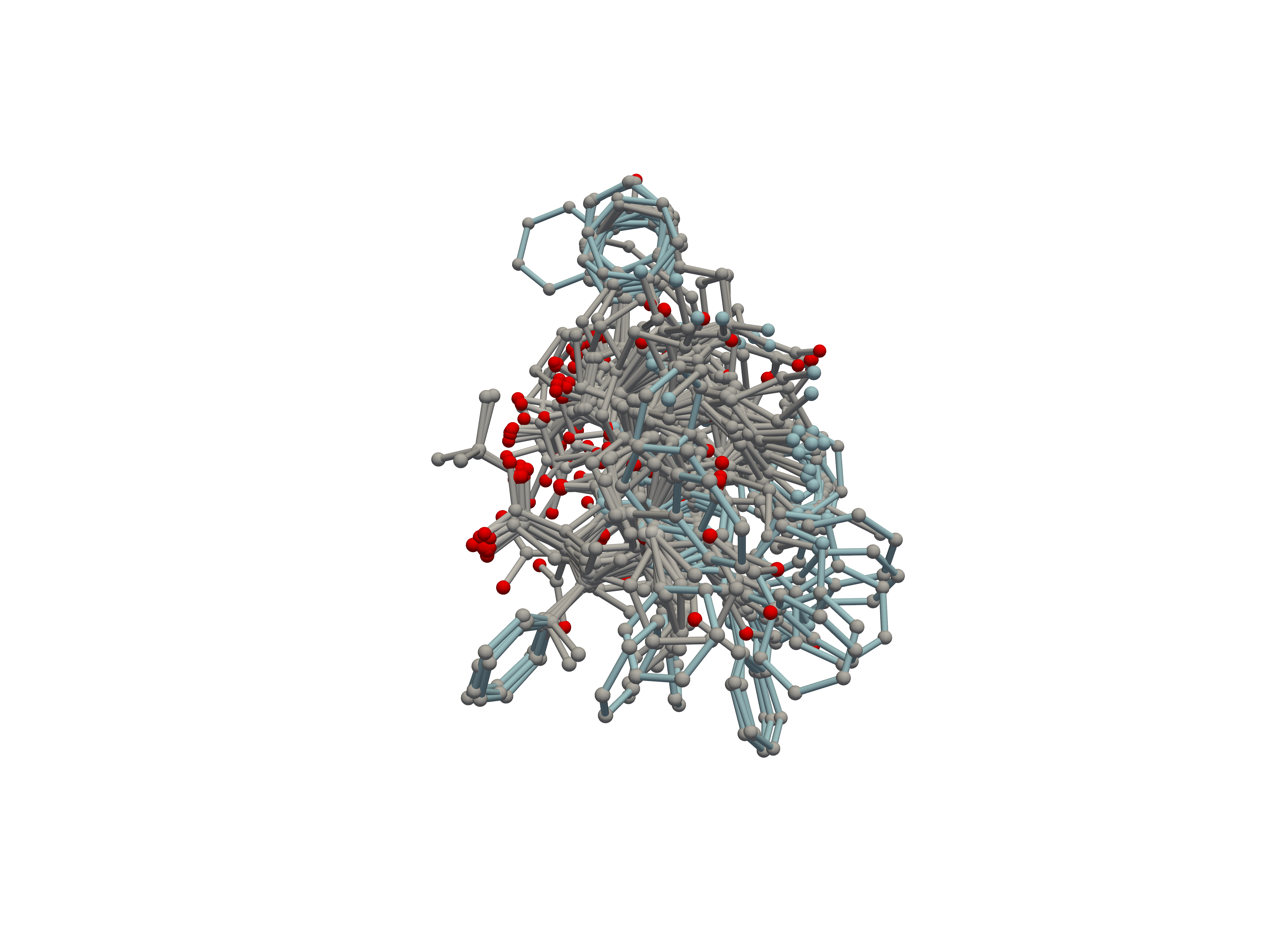

Supplement: Supplementary file 1 [file pharmaceuticals-18-00440-s001.zip › File S1/ACE_SEH_2025-02-21_11-36-26/Alignments/aligned_molecules.png]

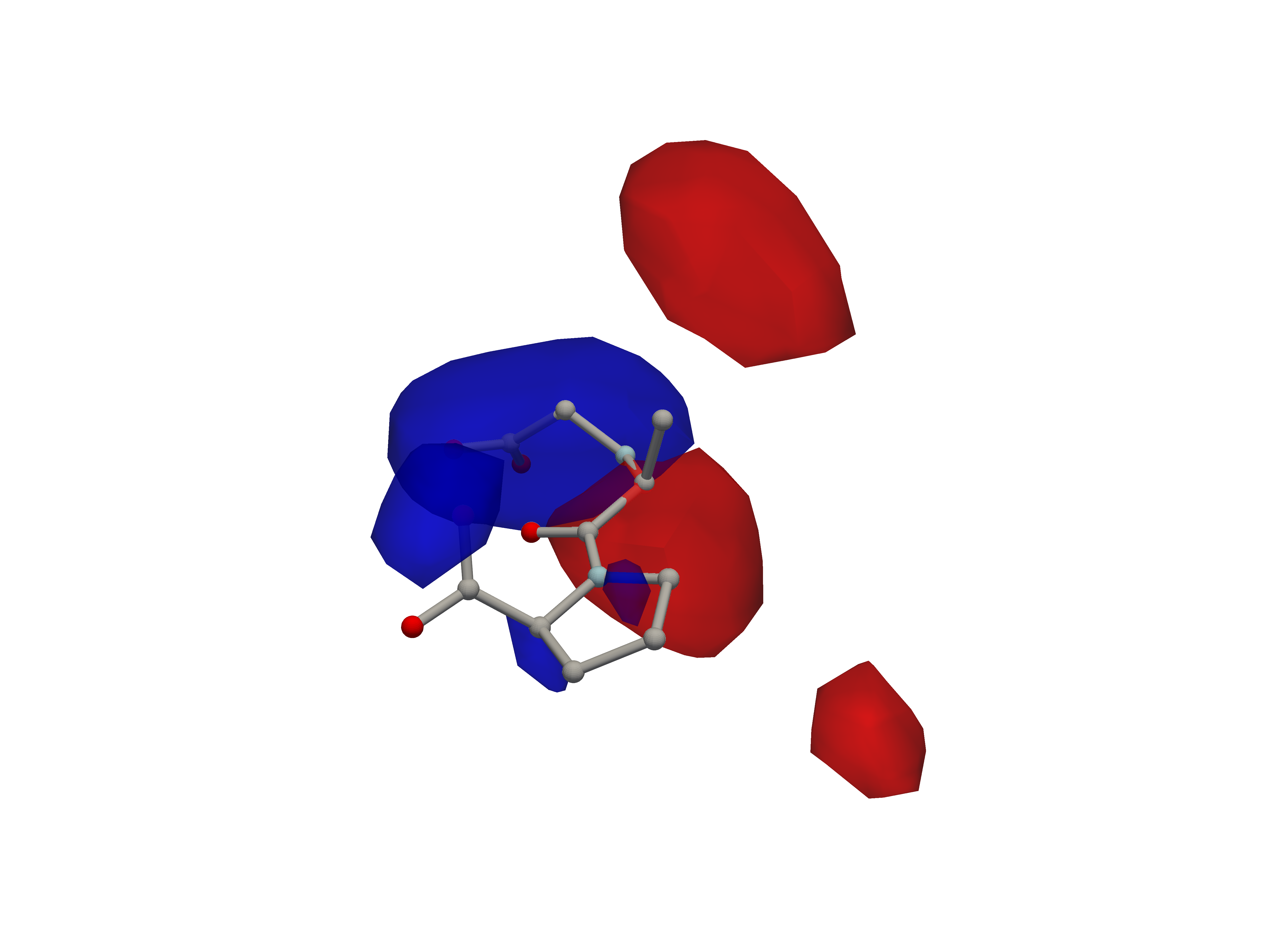

Supplement: Supplementary file 1 [file pharmaceuticals-18-00440-s001.zip › File S1/ACE_SEH_2025-02-21_11-36-26/Contour_Plots/electrostatic_field_contourplot.png]

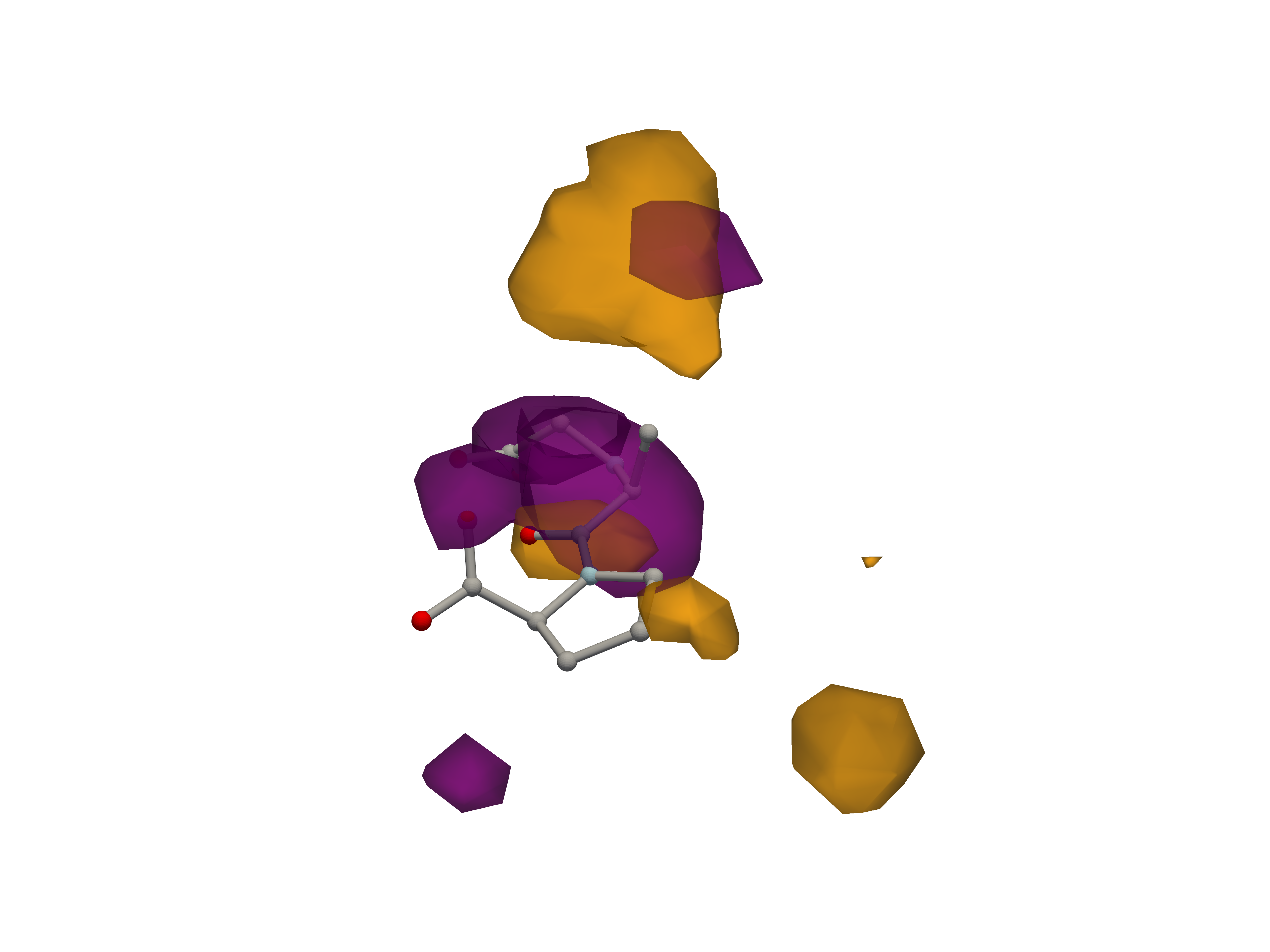

Supplement: Supplementary file 1 [file pharmaceuticals-18-00440-s001.zip › File S1/ACE_SEH_2025-02-21_11-36-26/Contour_Plots/hydrophobic_field_contourplot.png]

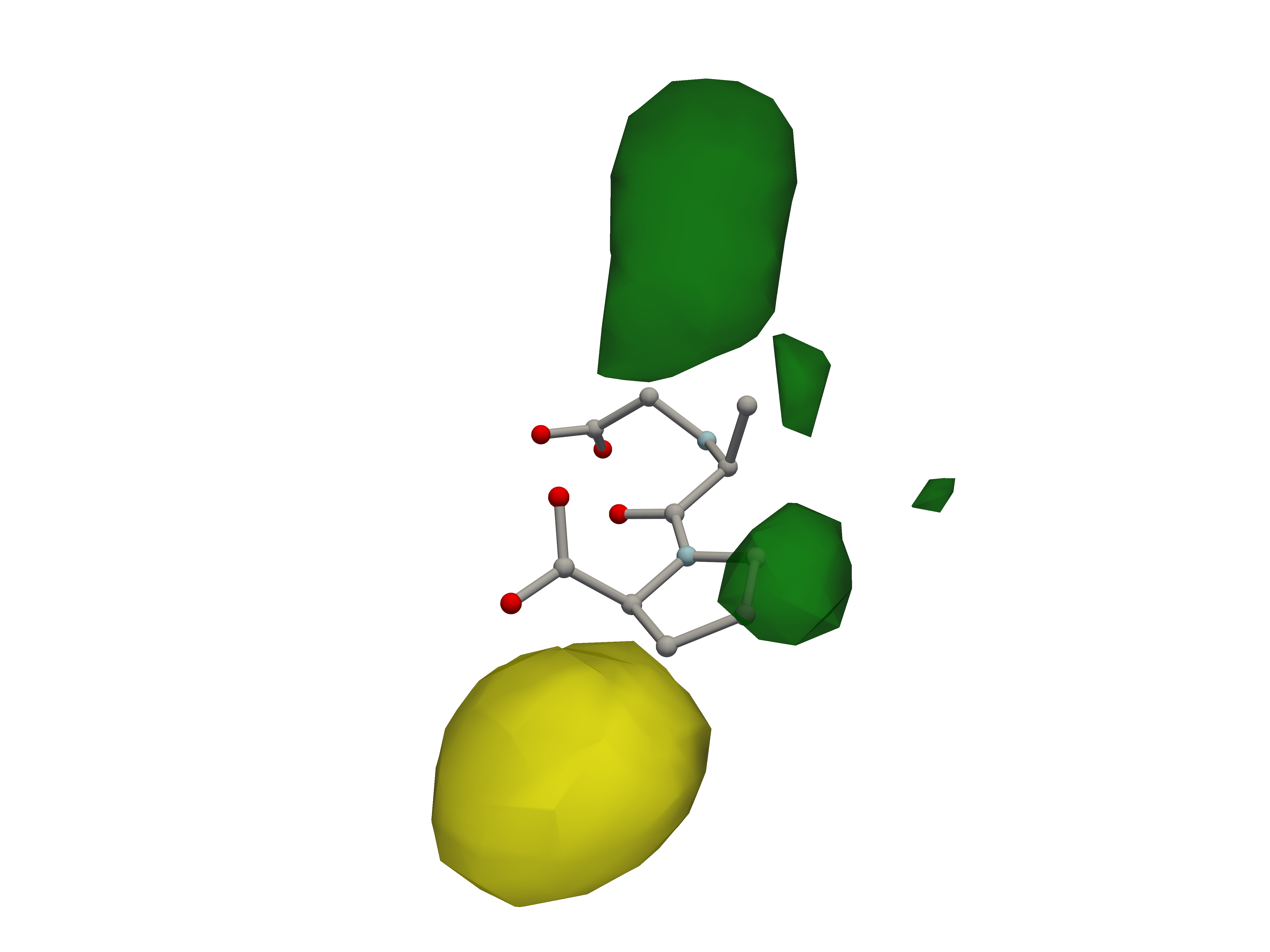

Supplement: Supplementary file 1 [file pharmaceuticals-18-00440-s001.zip › File S1/ACE_SEH_2025-02-21_11-36-26/Contour_Plots/steric_field_contourplot.png]

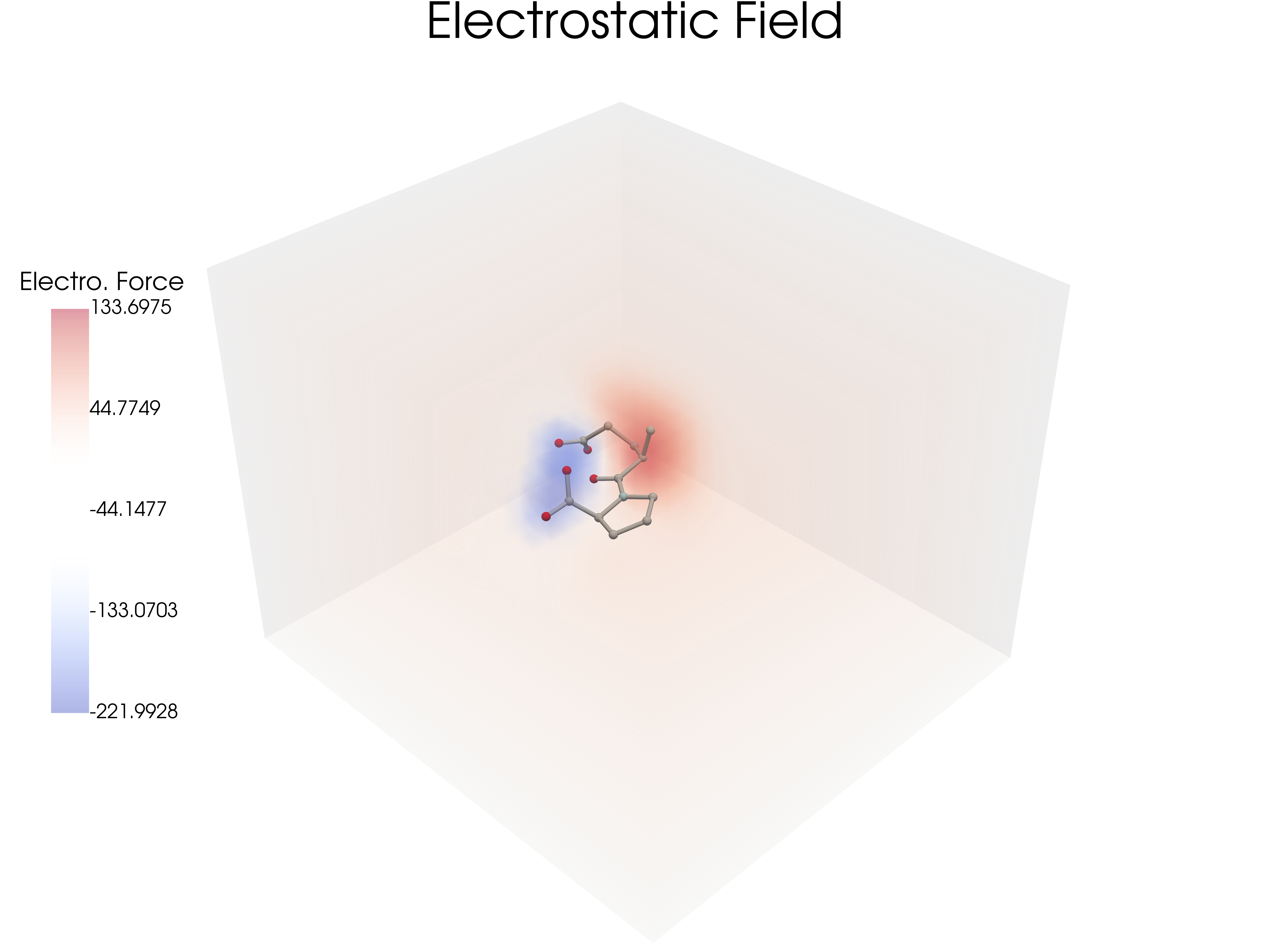

Supplement: Supplementary file 1 [file pharmaceuticals-18-00440-s001.zip › File S1/ACE_SEH_2025-02-21_11-36-26/Field_Plots/electrostatic.png]

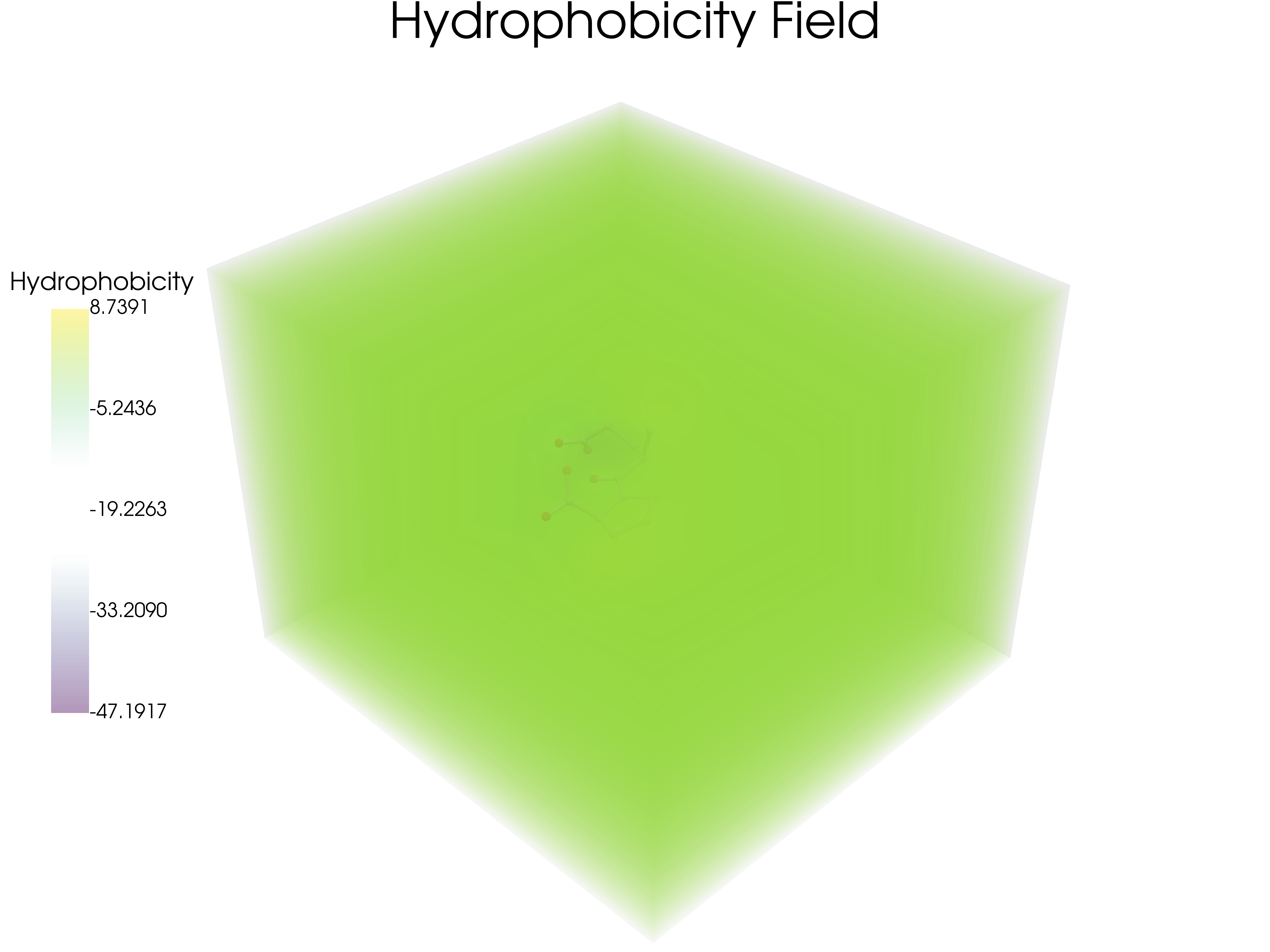

Supplement: Supplementary file 1 [file pharmaceuticals-18-00440-s001.zip › File S1/ACE_SEH_2025-02-21_11-36-26/Field_Plots/hydrophobic.png]

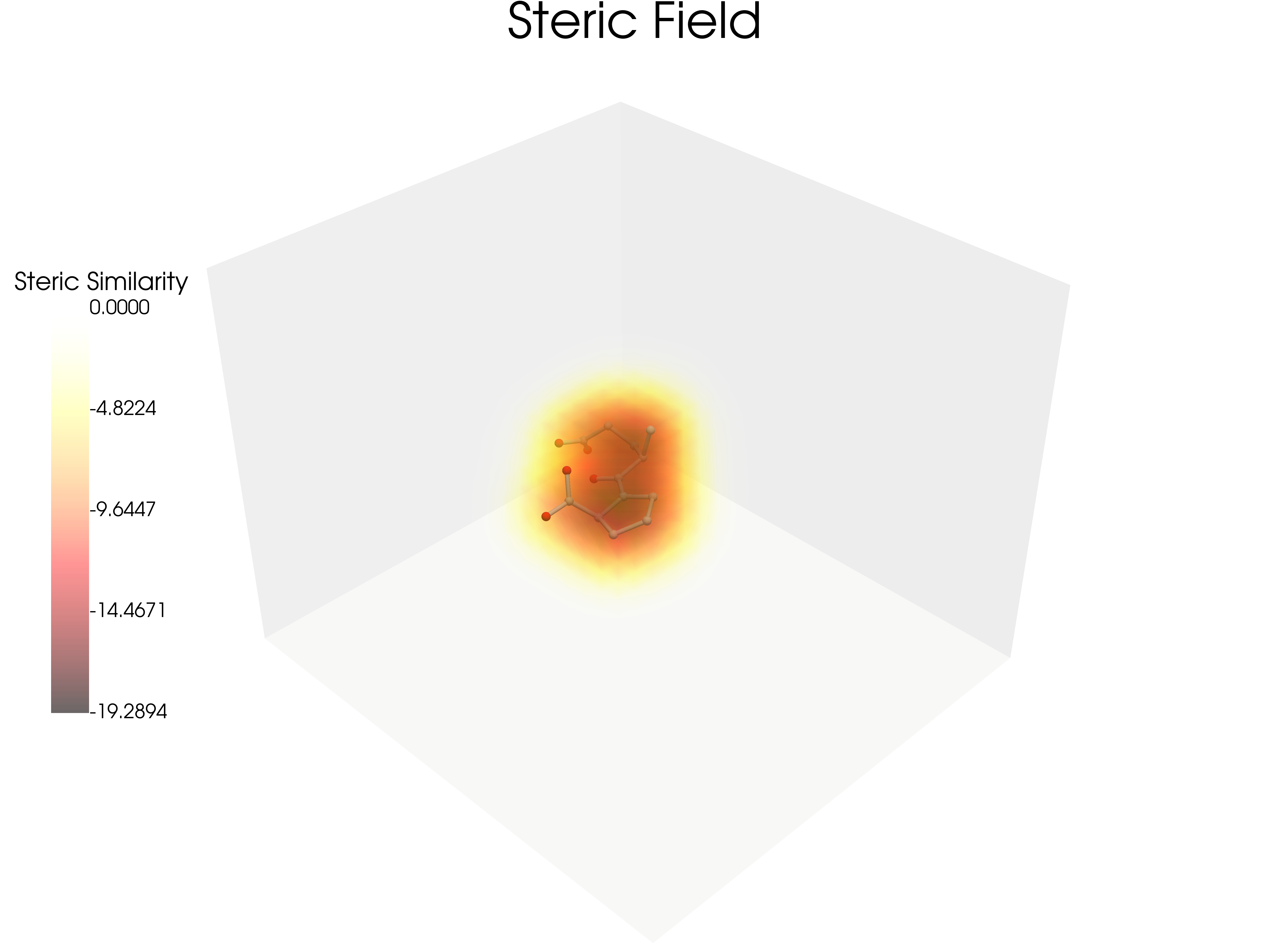

Supplement: Supplementary file 1 [file pharmaceuticals-18-00440-s001.zip › File S1/ACE_SEH_2025-02-21_11-36-26/Field_Plots/steric.png]

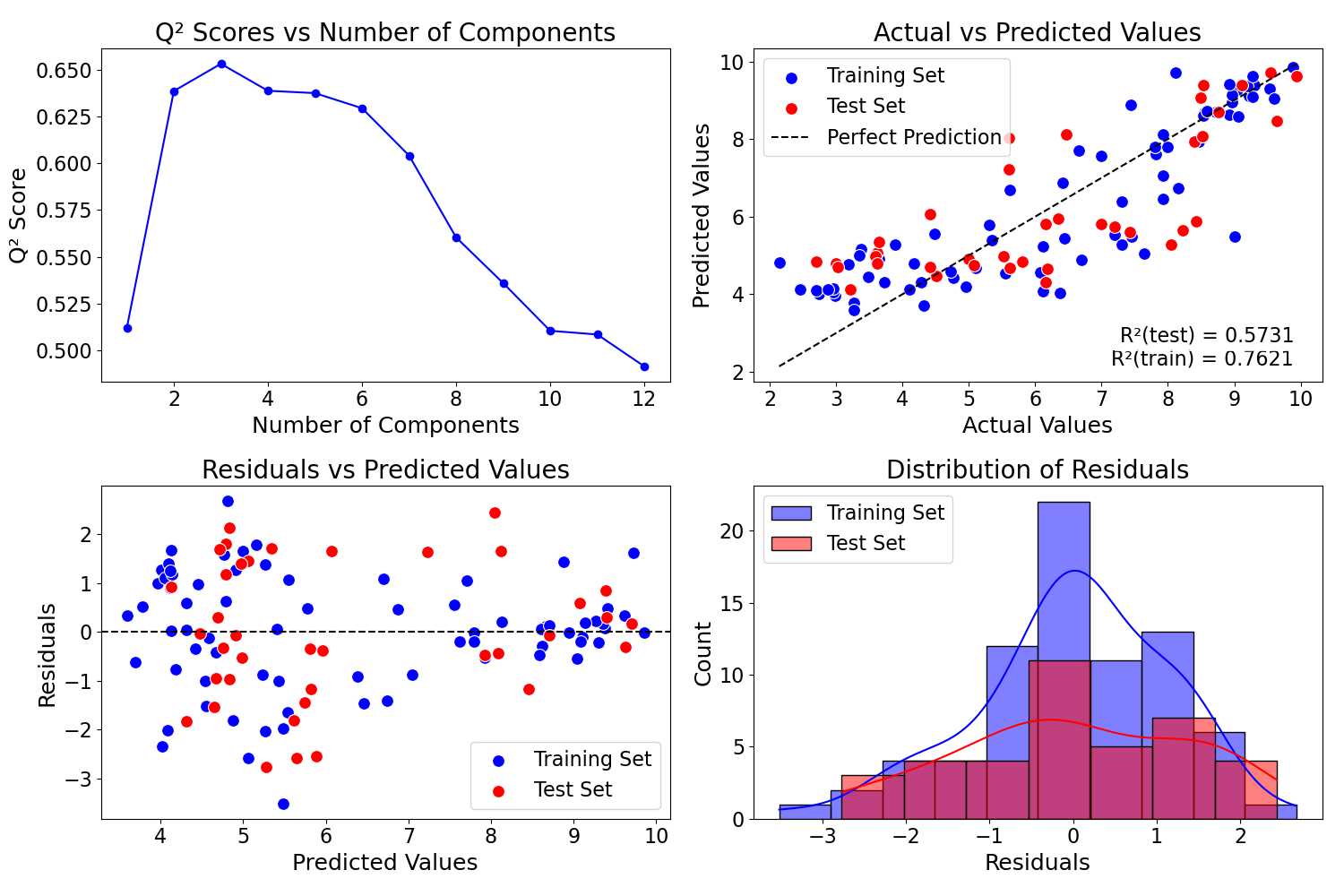

Supplement: Supplementary file 1 [file pharmaceuticals-18-00440-s001.zip › File S1/ACE_SEH_2025-02-21_11-36-26/PLS_Analysis/PLSplots.png]

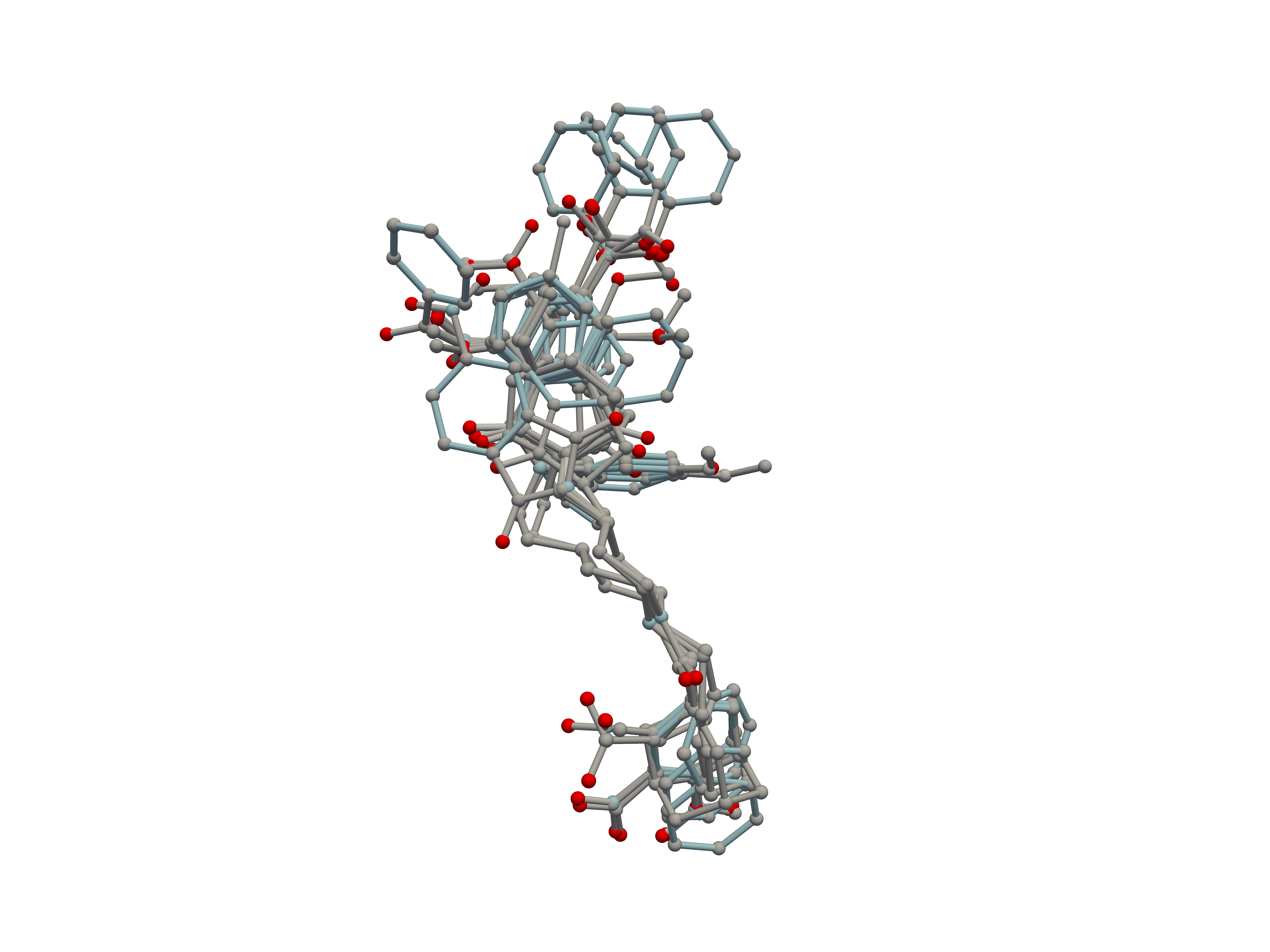

Supplement: Supplementary file 1 [file pharmaceuticals-18-00440-s001.zip › File S1/AChE_all_2025-02-21_11-40-41/Alignments/aligned_molecules.png]

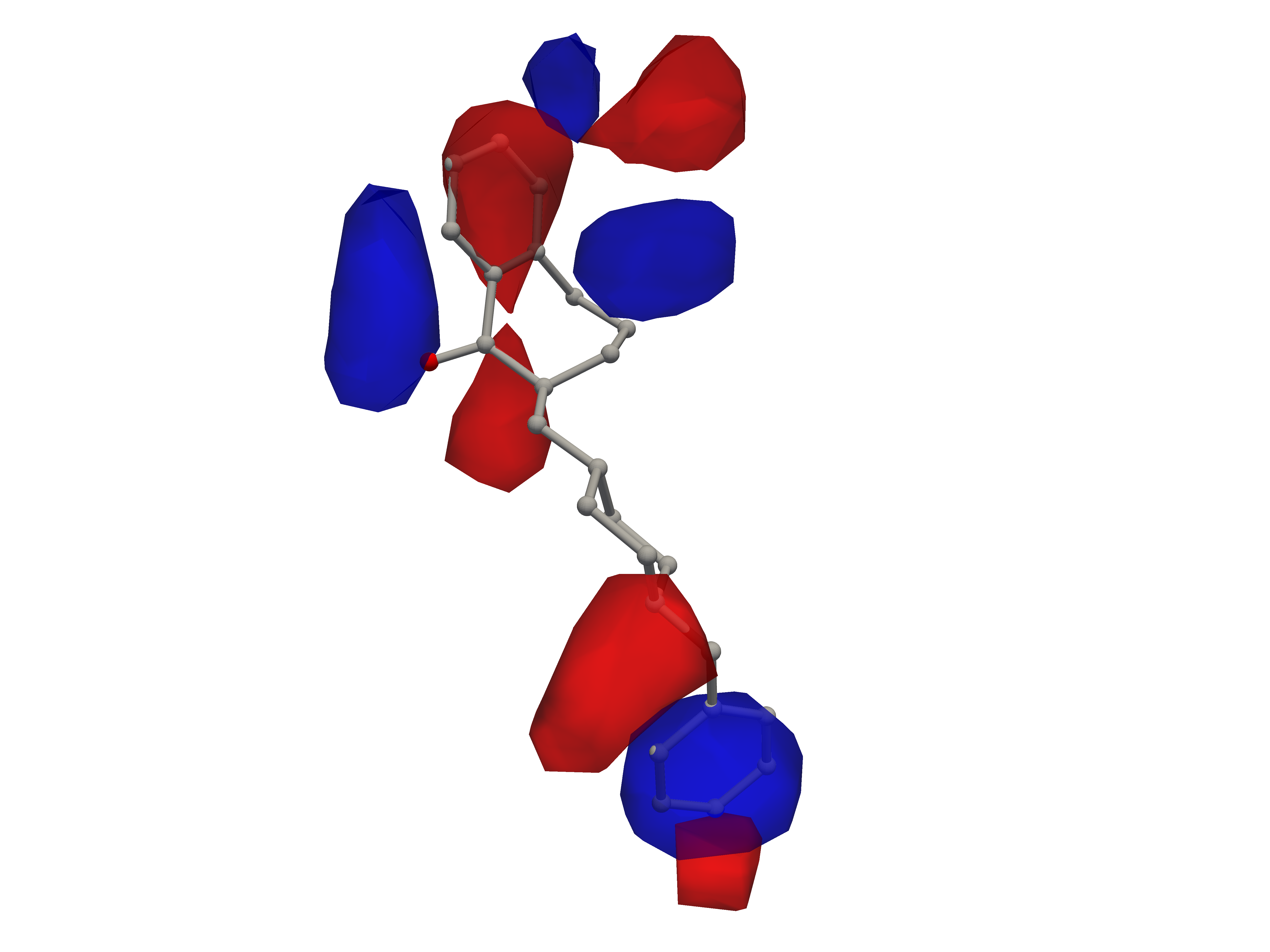

Supplement: Supplementary file 1 [file pharmaceuticals-18-00440-s001.zip › File S1/AChE_all_2025-02-21_11-40-41/Contour_Plots/electrostatic_field_contourplot.png]

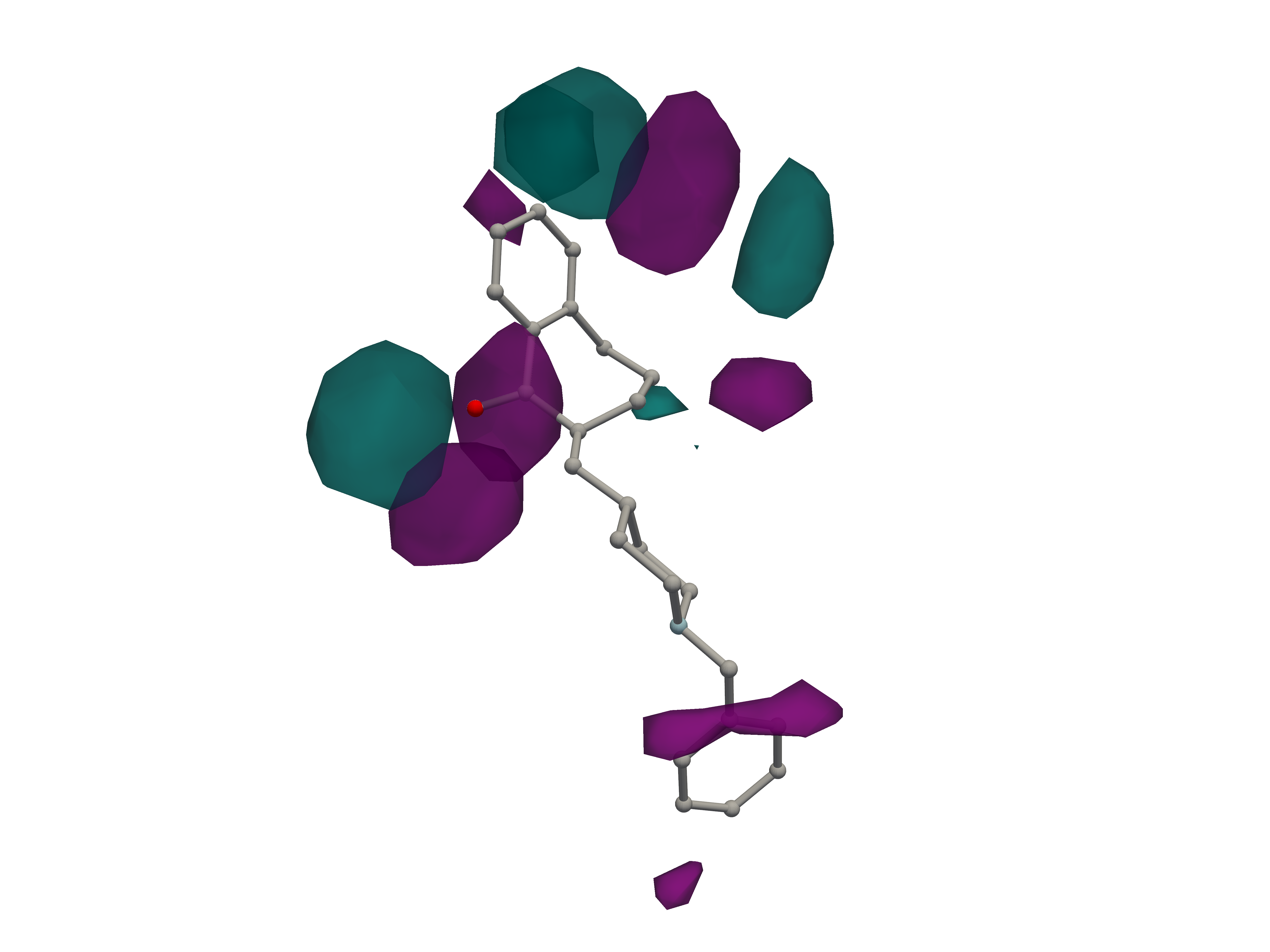

Supplement: Supplementary file 1 [file pharmaceuticals-18-00440-s001.zip › File S1/AChE_all_2025-02-21_11-40-41/Contour_Plots/hbond_acceptor_field_contourplot.png]

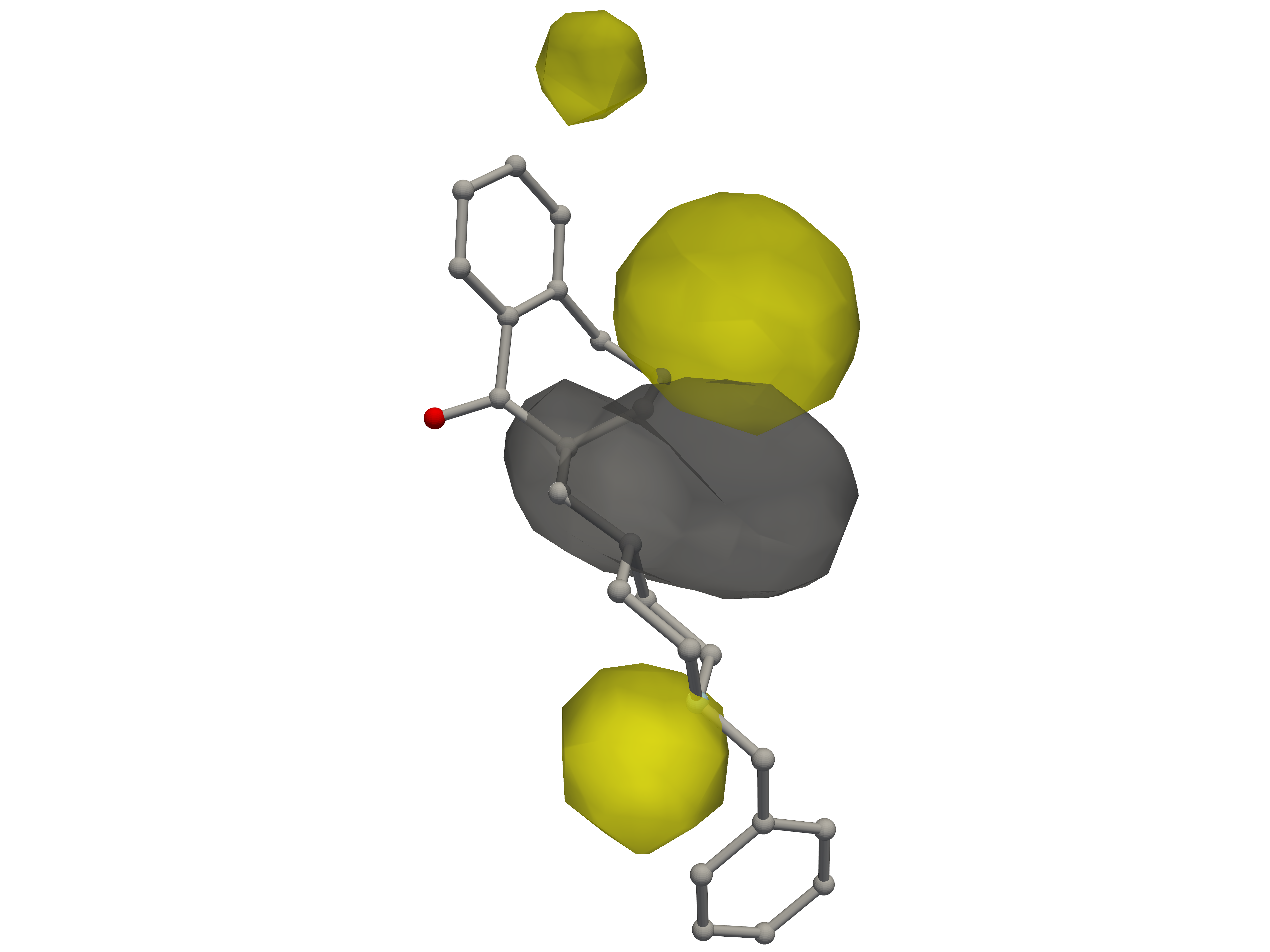

Supplement: Supplementary file 1 [file pharmaceuticals-18-00440-s001.zip › File S1/AChE_all_2025-02-21_11-40-41/Contour_Plots/hbond_donor_field_contourplot.png]

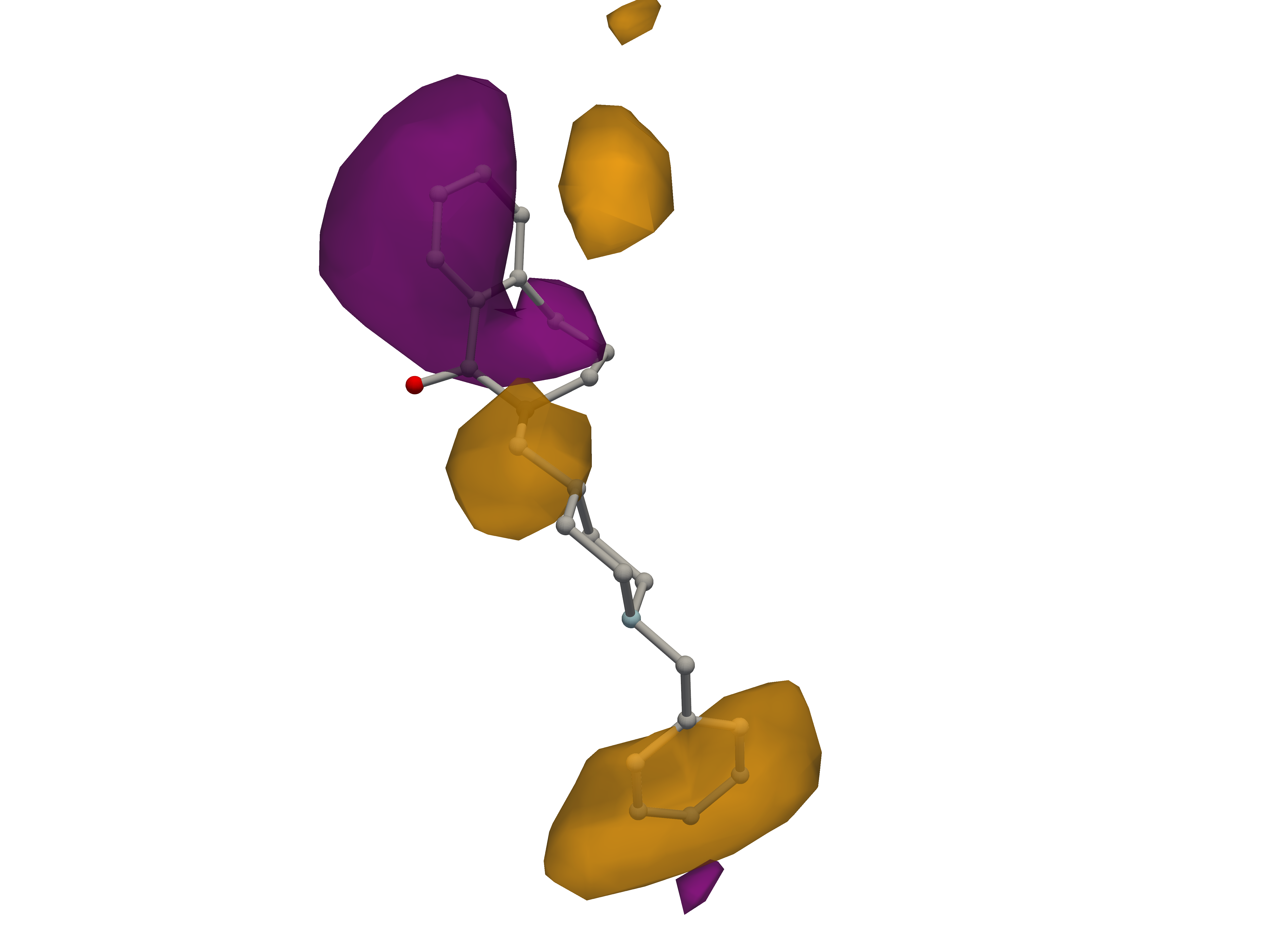

Supplement: Supplementary file 1 [file pharmaceuticals-18-00440-s001.zip › File S1/AChE_all_2025-02-21_11-40-41/Contour_Plots/hydrophobic_field_contourplot.png]

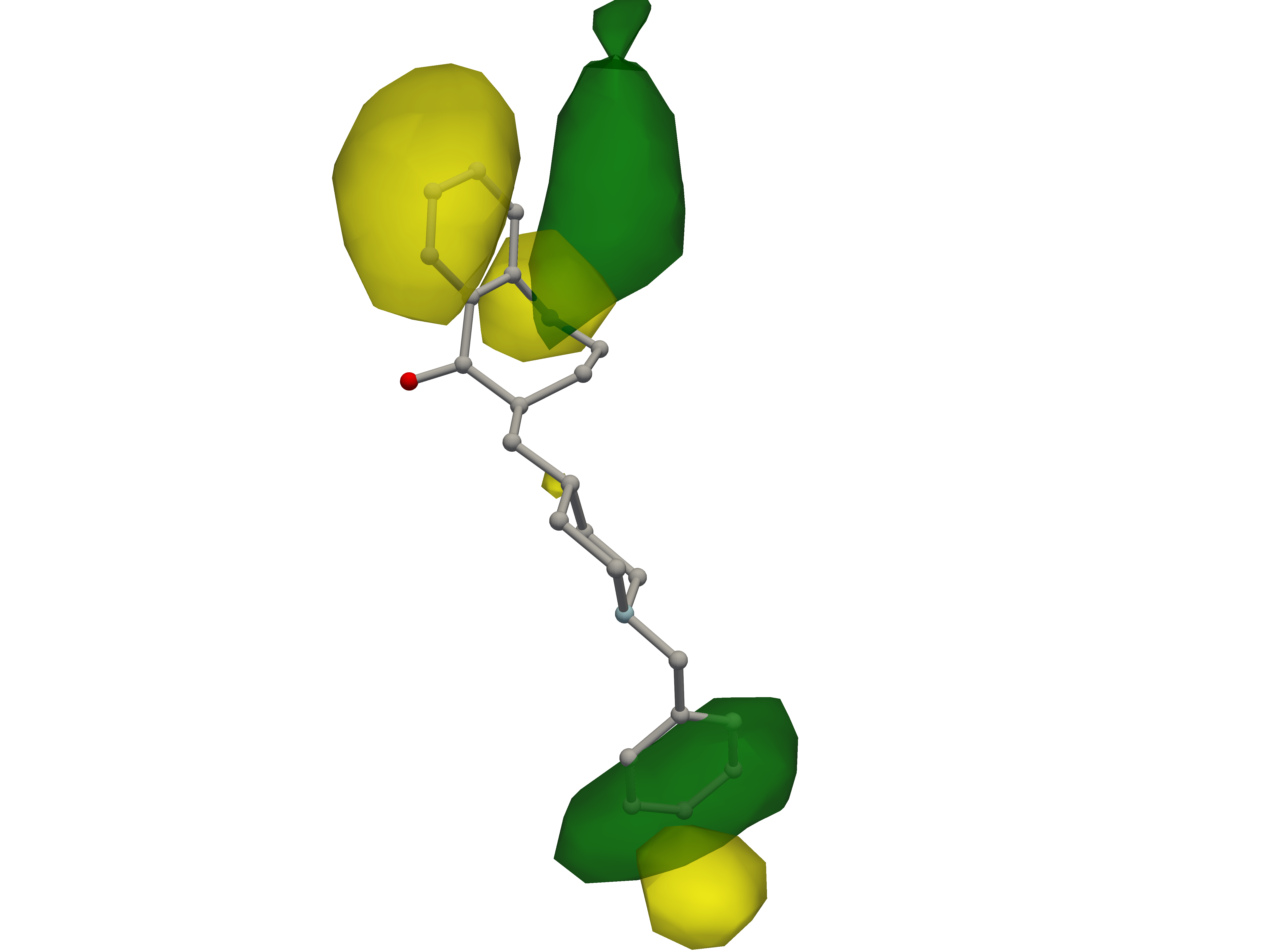

Supplement: Supplementary file 1 [file pharmaceuticals-18-00440-s001.zip › File S1/AChE_all_2025-02-21_11-40-41/Contour_Plots/steric_field_contourplot.png]

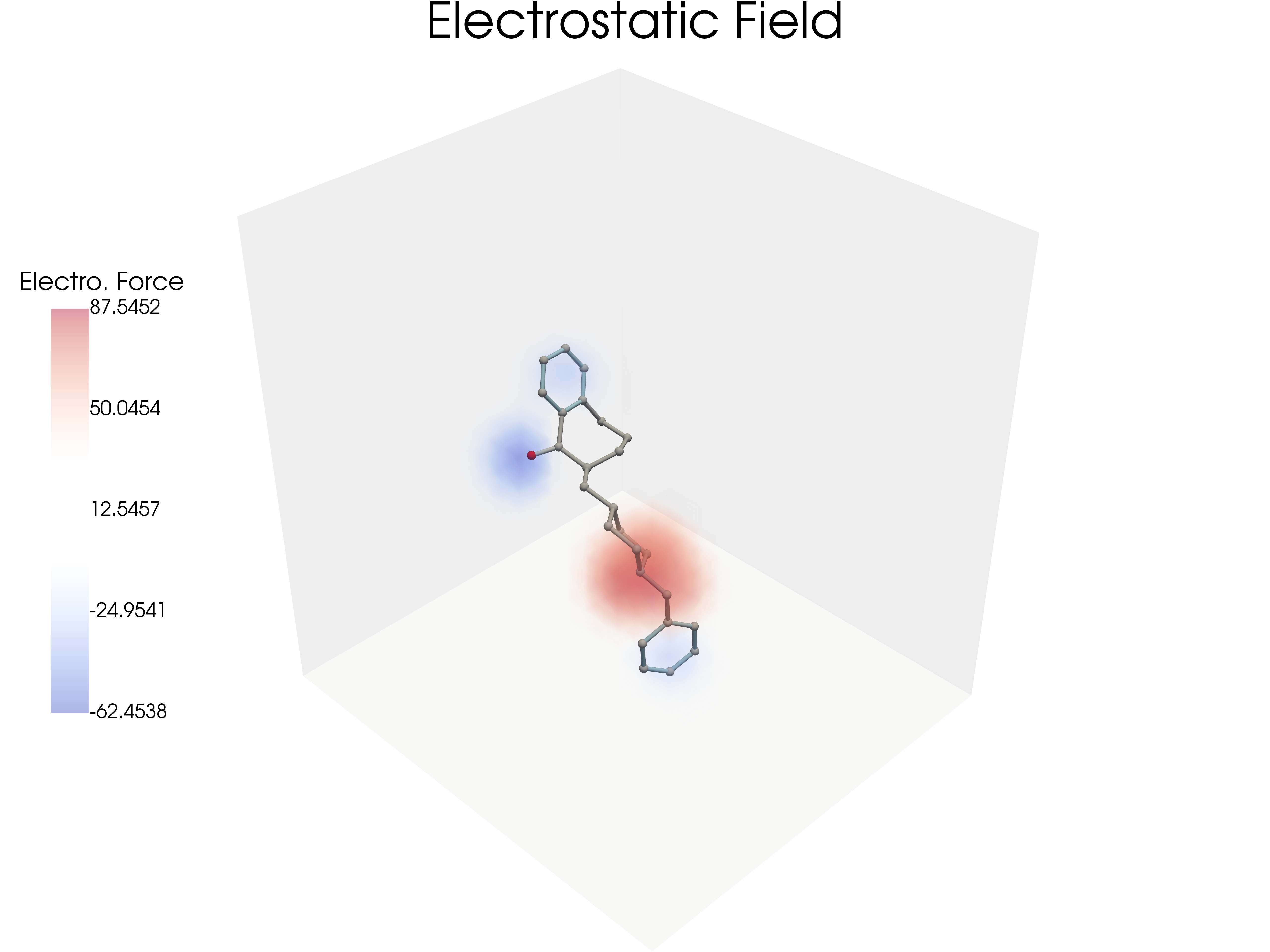

Supplement: Supplementary file 1 [file pharmaceuticals-18-00440-s001.zip › File S1/AChE_all_2025-02-21_11-40-41/Field_Plots/electrostatic.png]

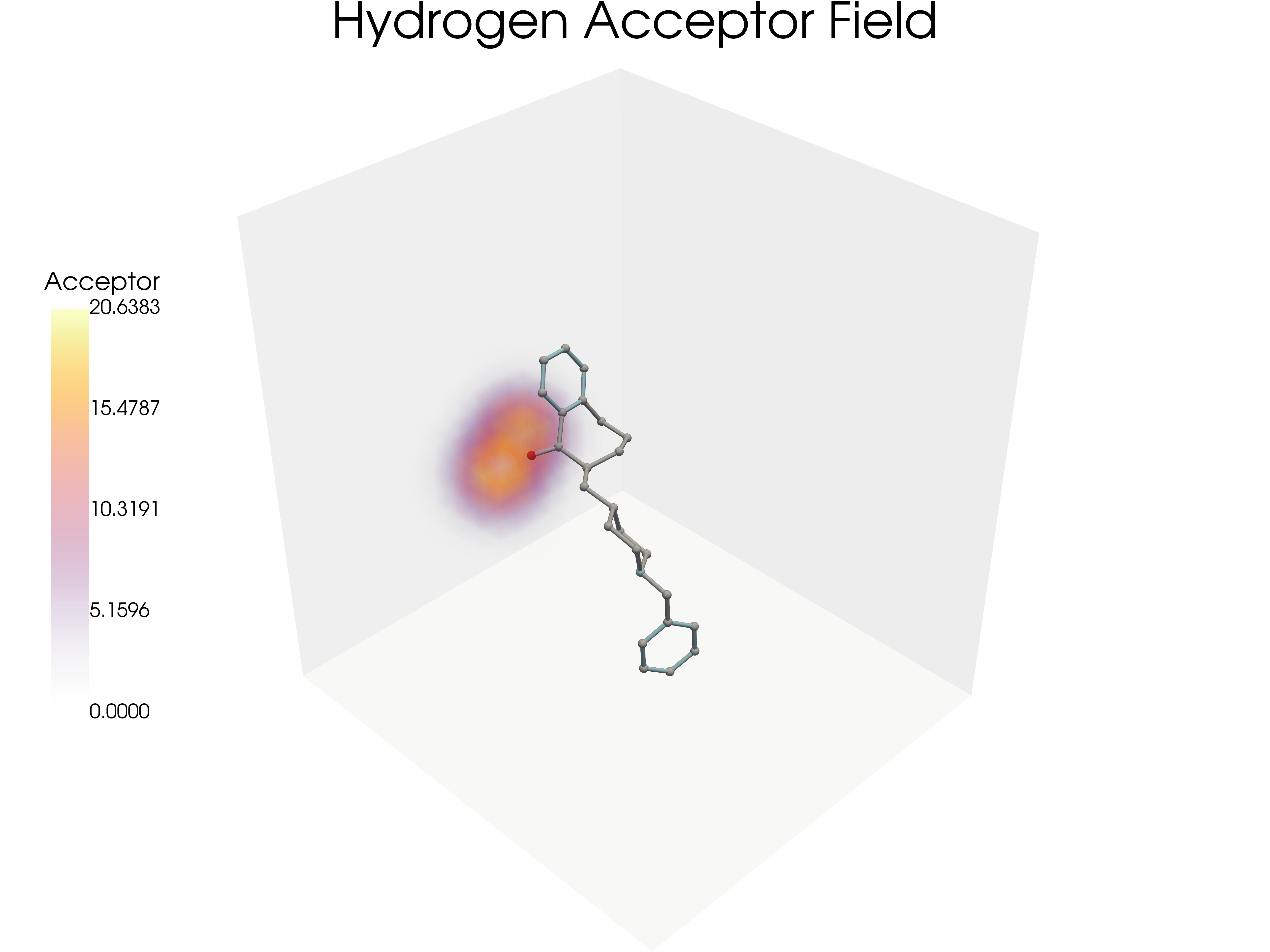

Supplement: Supplementary file 1 [file pharmaceuticals-18-00440-s001.zip › File S1/AChE_all_2025-02-21_11-40-41/Field_Plots/hbond_acceptor.png]

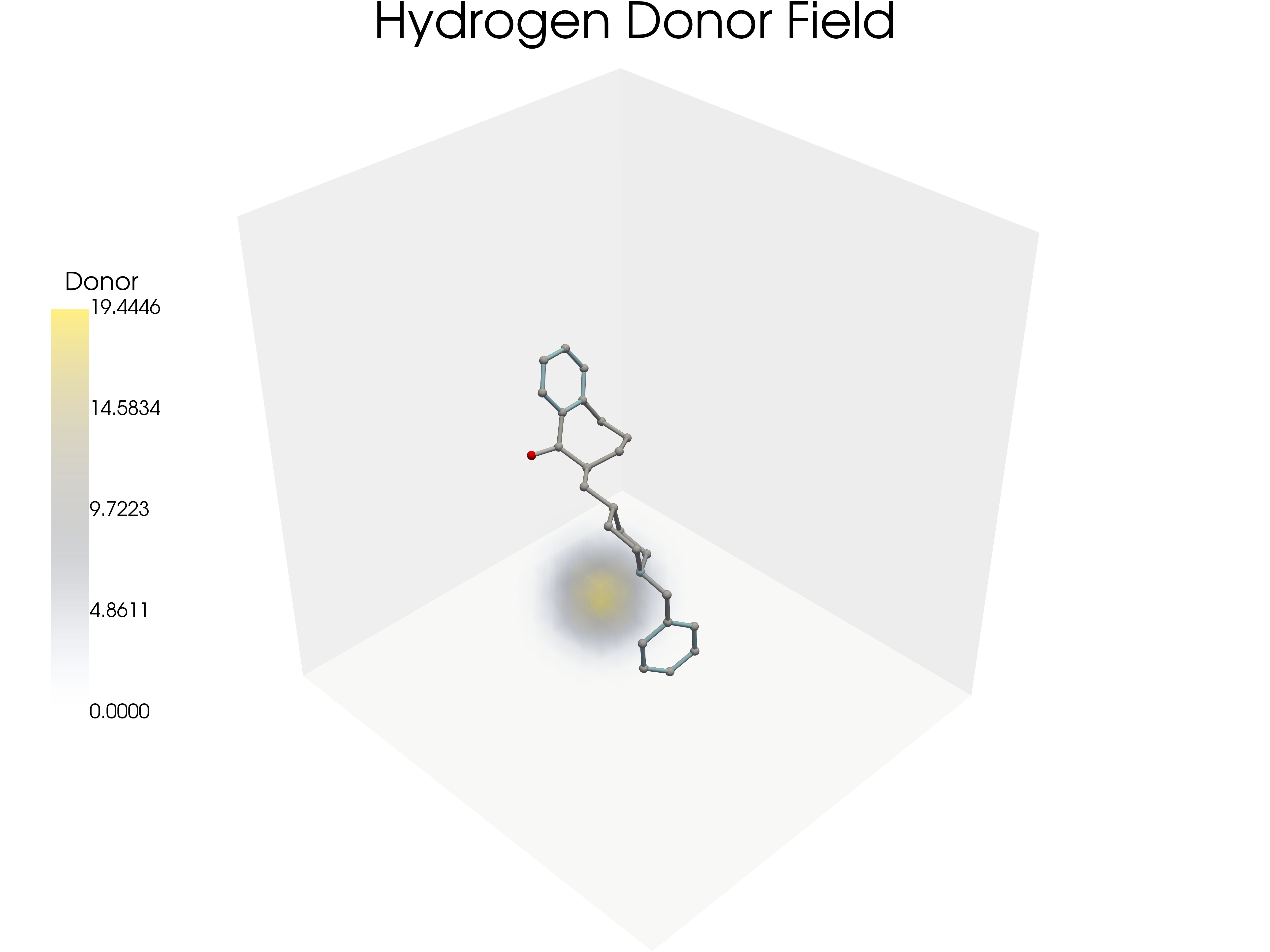

Supplement: Supplementary file 1 [file pharmaceuticals-18-00440-s001.zip › File S1/AChE_all_2025-02-21_11-40-41/Field_Plots/hbond_donor.png]

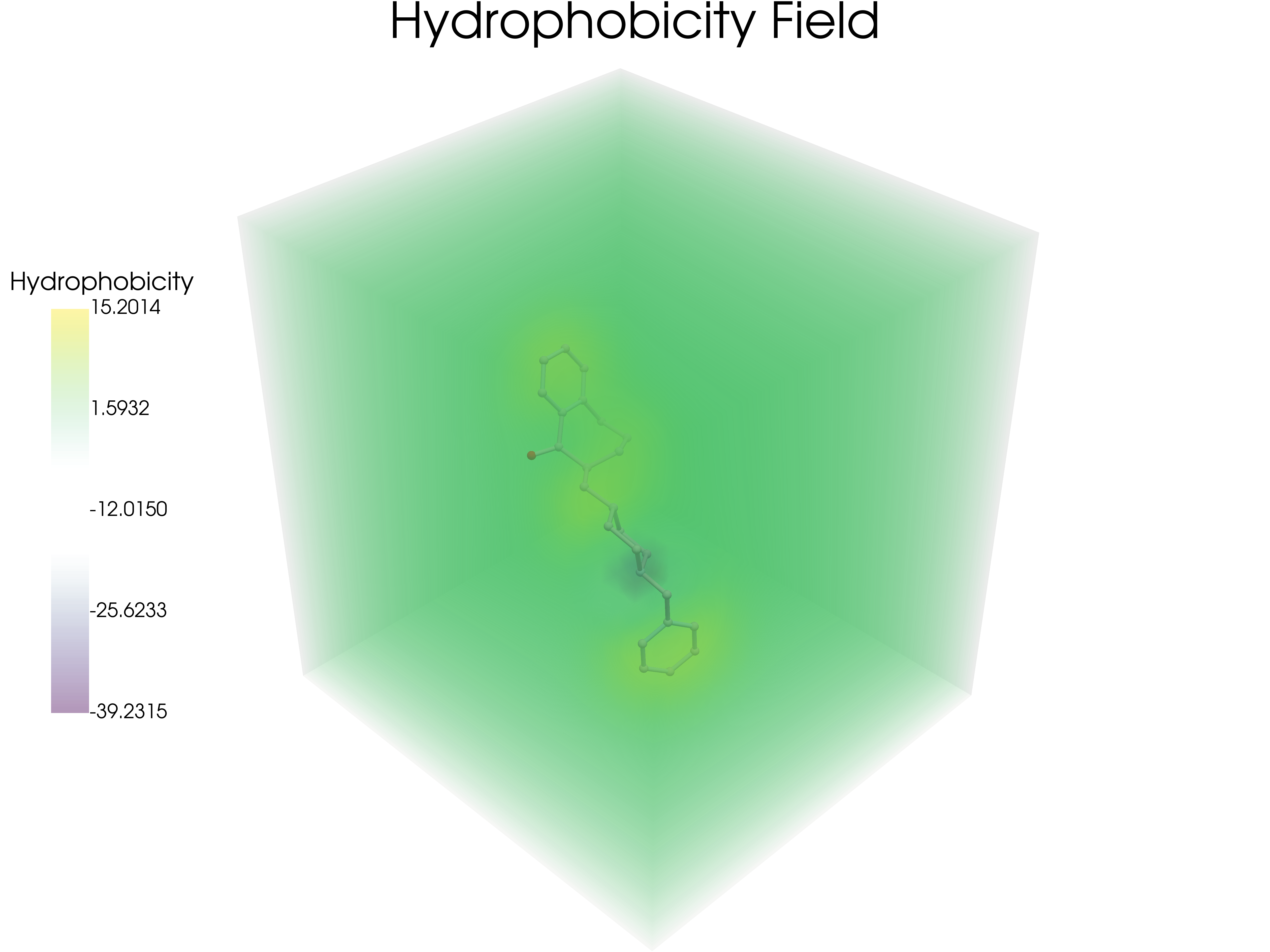

Supplement: Supplementary file 1 [file pharmaceuticals-18-00440-s001.zip › File S1/AChE_all_2025-02-21_11-40-41/Field_Plots/hydrophobic.png]

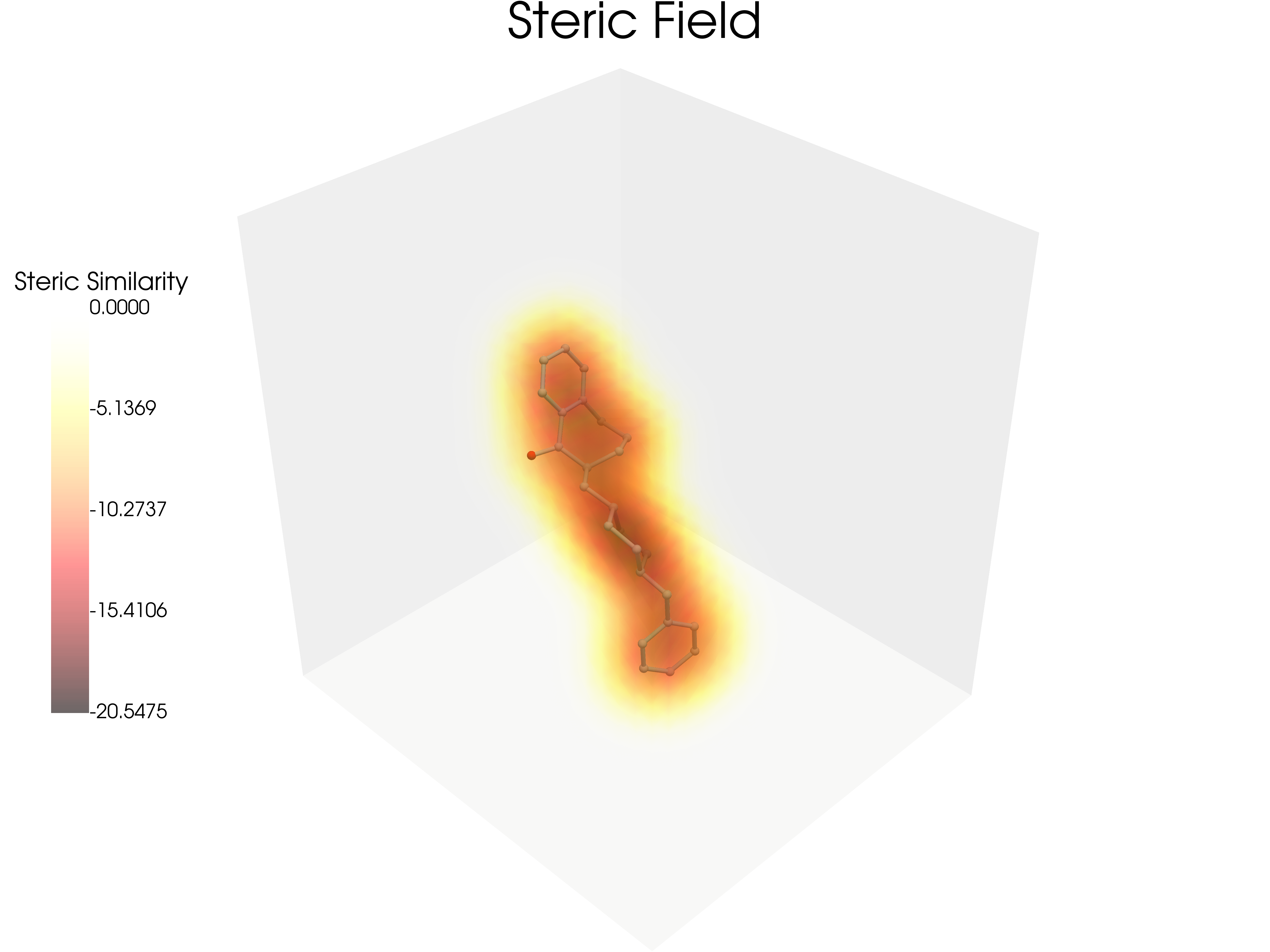

Supplement: Supplementary file 1 [file pharmaceuticals-18-00440-s001.zip › File S1/AChE_all_2025-02-21_11-40-41/Field_Plots/steric.png]

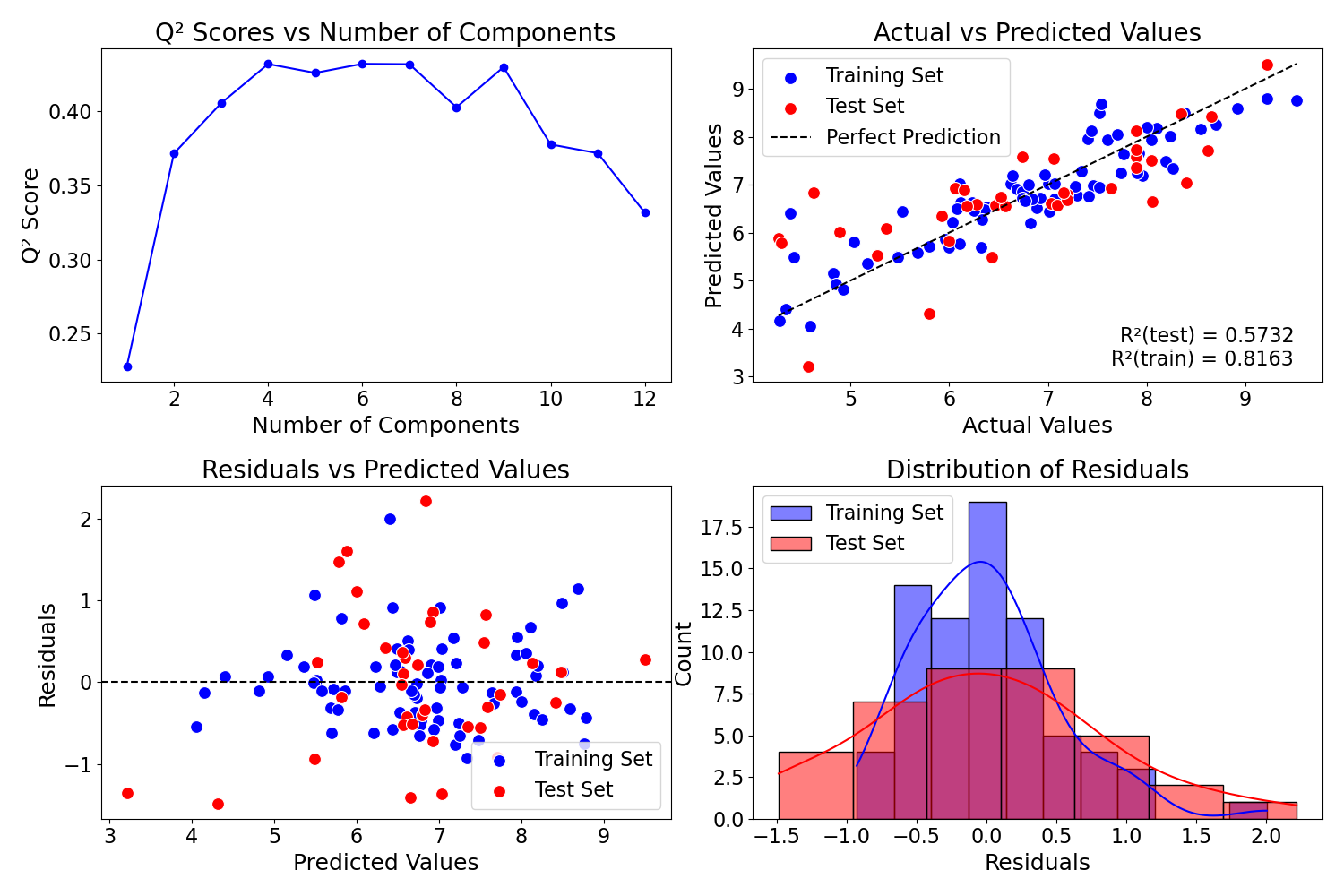

Supplement: Supplementary file 1 [file pharmaceuticals-18-00440-s001.zip › File S1/AChE_all_2025-02-21_11-40-41/PLS_Analysis/PLSplots.png]

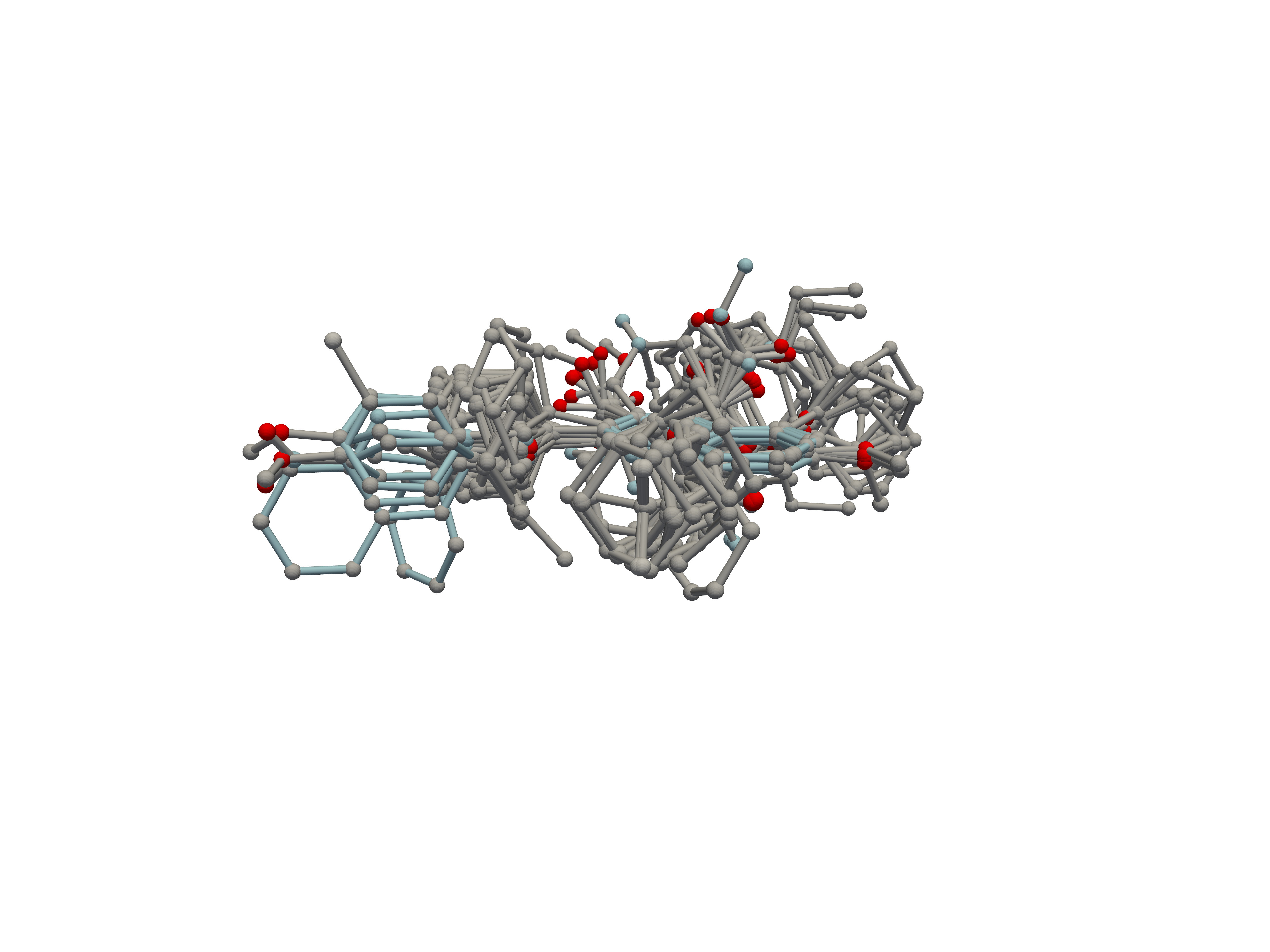

Supplement: Supplementary file 1 [file pharmaceuticals-18-00440-s001.zip › File S1/ATA_all_2025-02-21_11-51-35/Alignments/aligned_molecules.png]

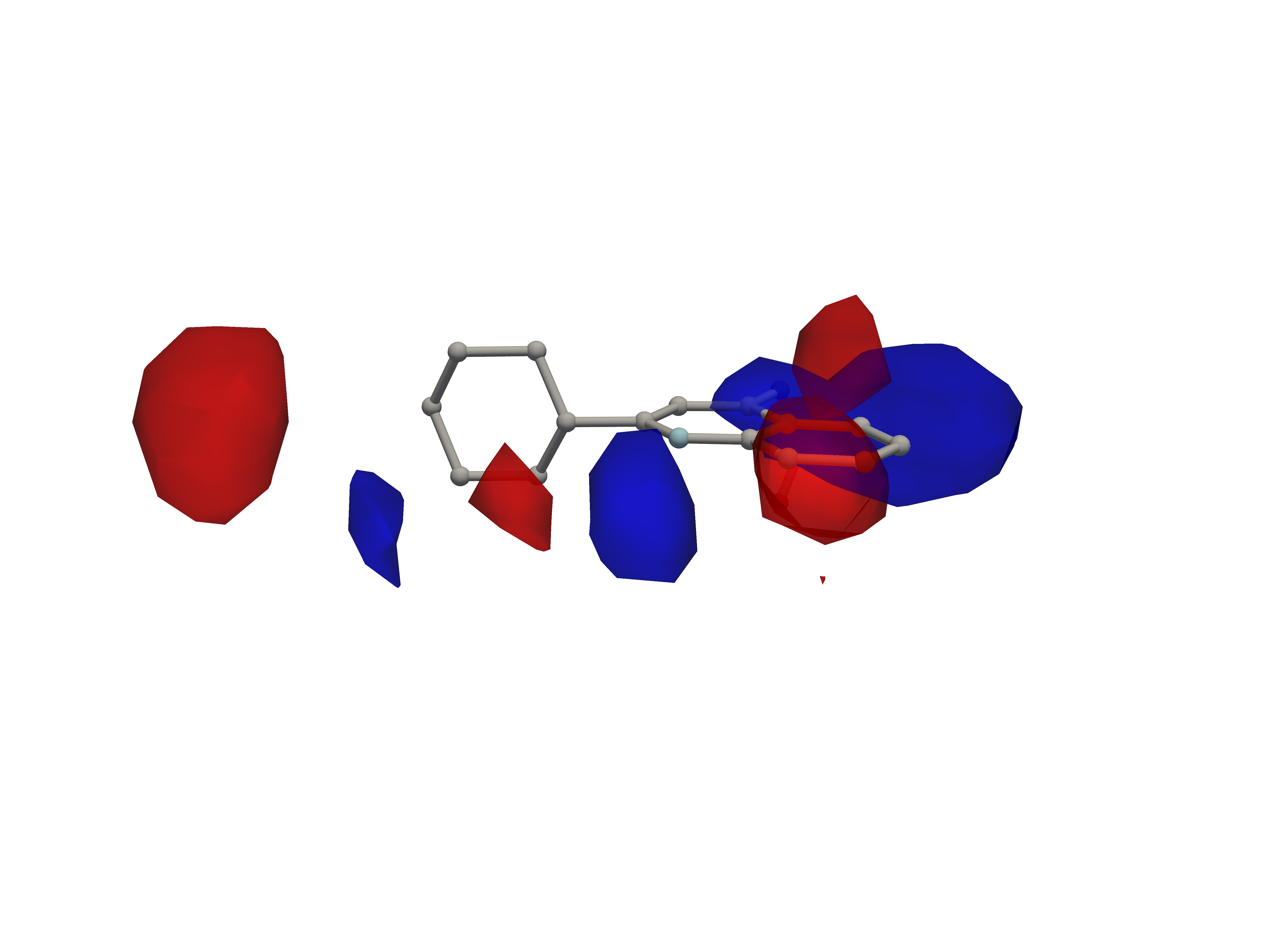

Supplement: Supplementary file 1 [file pharmaceuticals-18-00440-s001.zip › File S1/ATA_all_2025-02-21_11-51-35/Contour_Plots/electrostatic_field_contourplot.png]

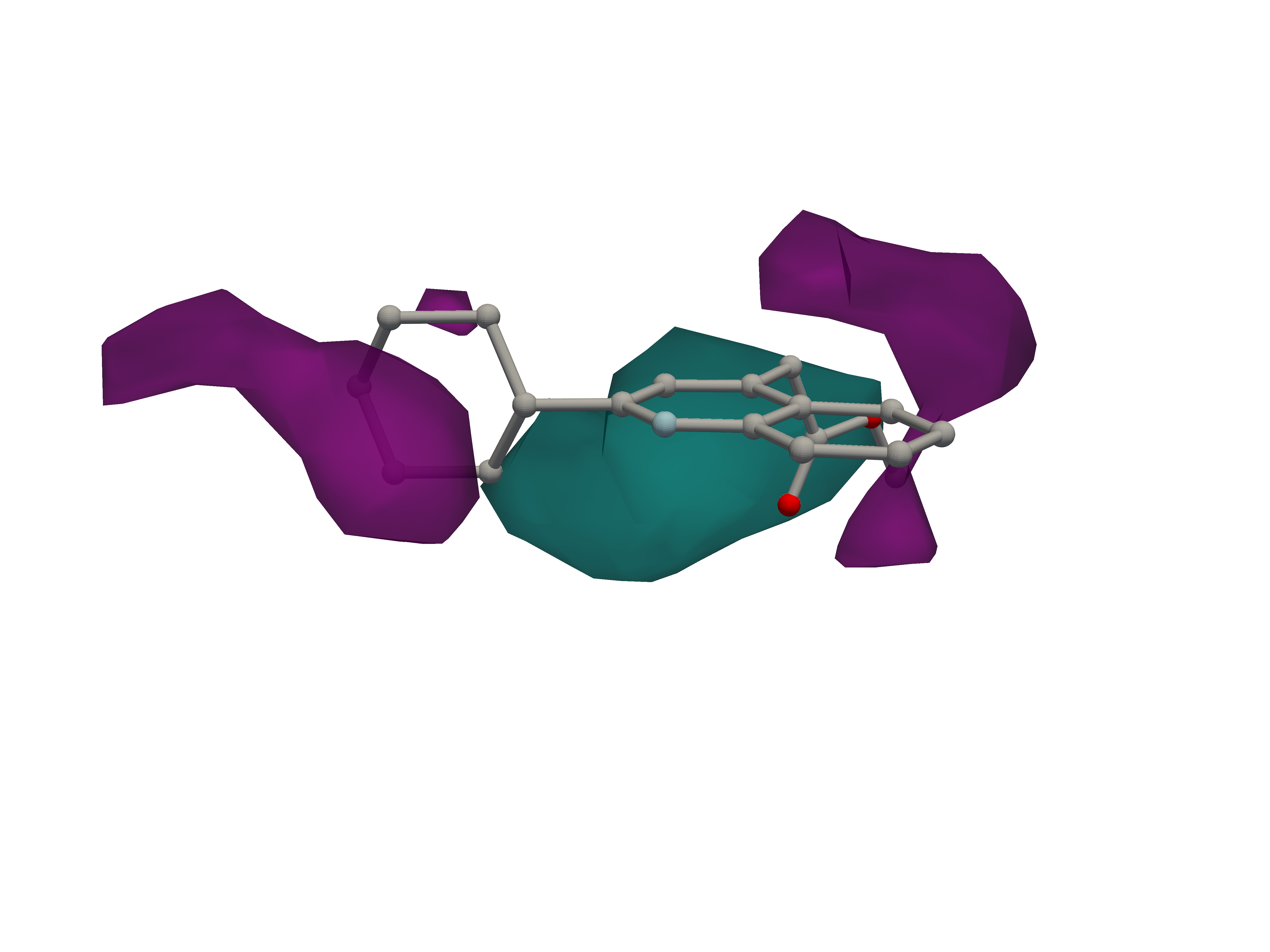

Supplement: Supplementary file 1 [file pharmaceuticals-18-00440-s001.zip › File S1/ATA_all_2025-02-21_11-51-35/Contour_Plots/hbond_acceptor_field_contourplot.png]

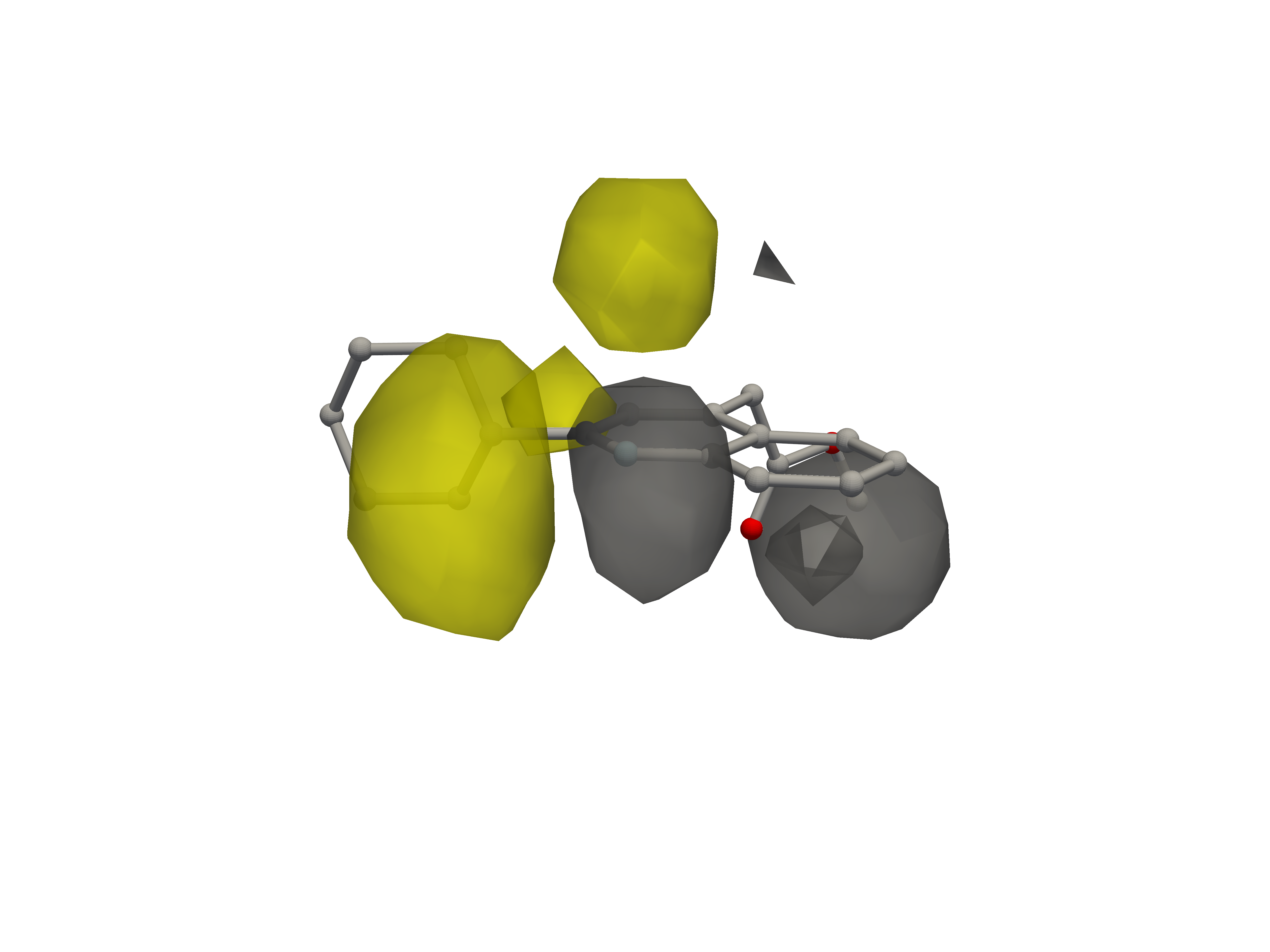

Supplement: Supplementary file 1 [file pharmaceuticals-18-00440-s001.zip › File S1/ATA_all_2025-02-21_11-51-35/Contour_Plots/hbond_donor_field_contourplot.png]

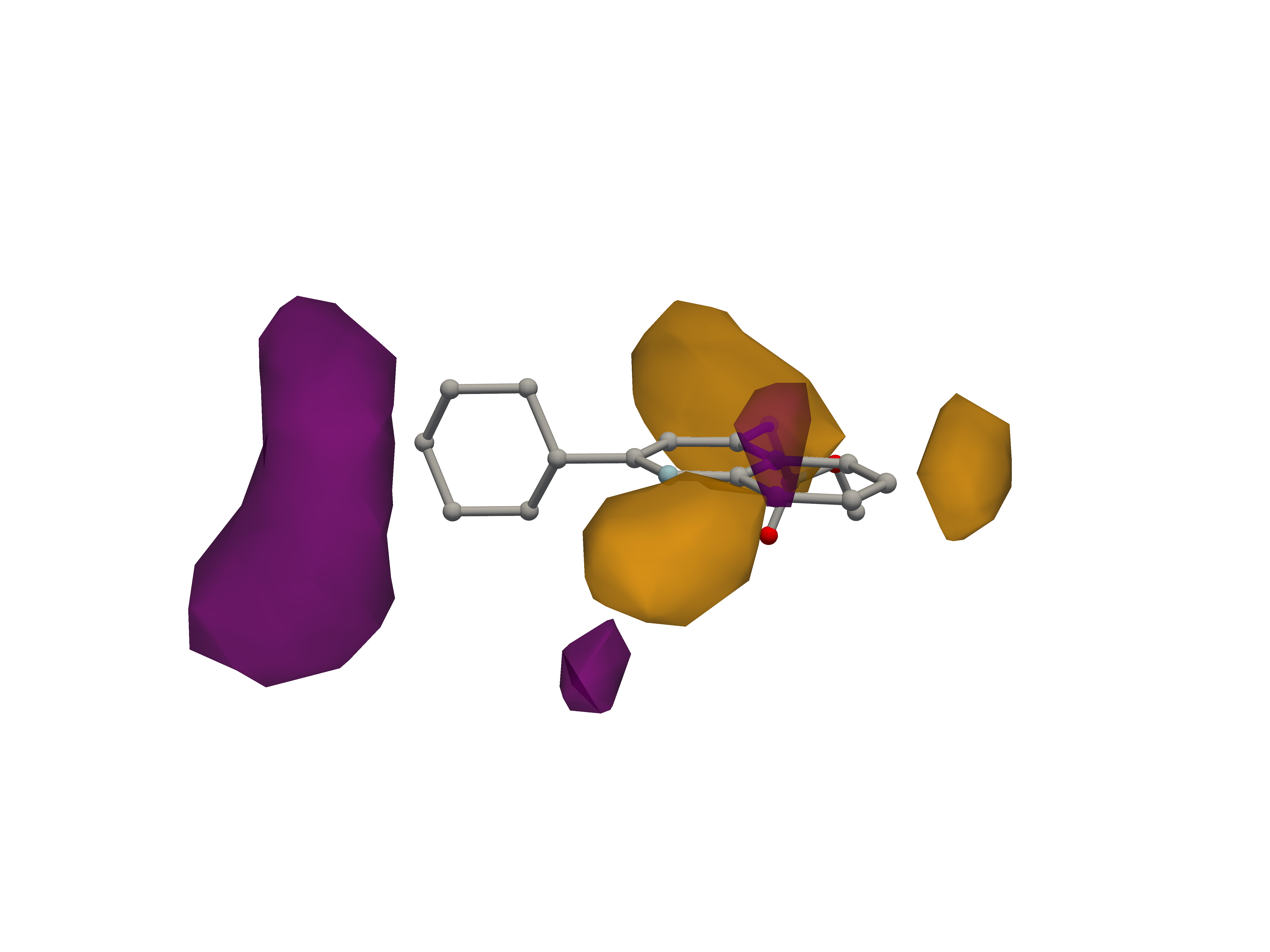

Supplement: Supplementary file 1 [file pharmaceuticals-18-00440-s001.zip › File S1/ATA_all_2025-02-21_11-51-35/Contour_Plots/hydrophobic_field_contourplot.png]

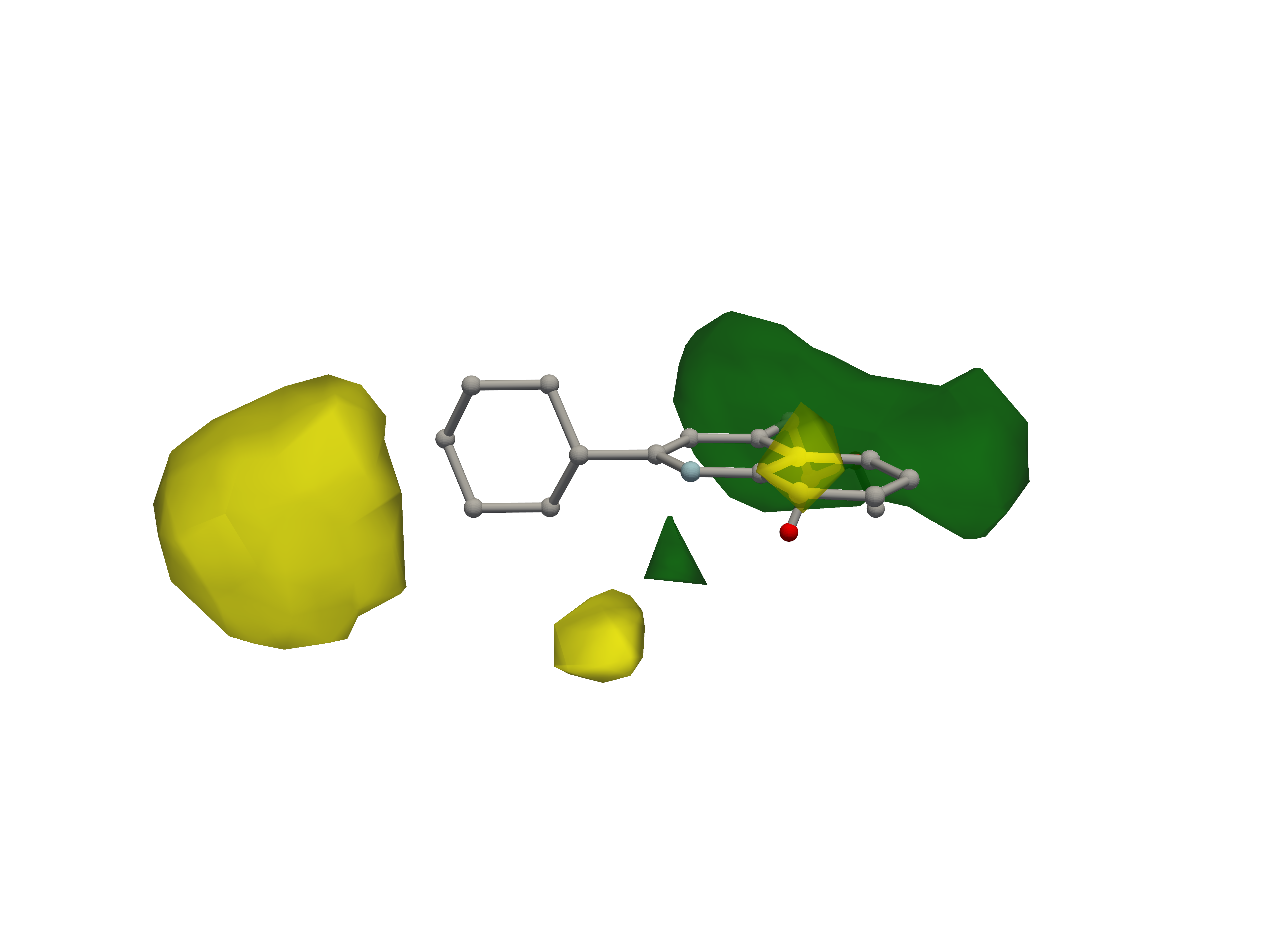

Supplement: Supplementary file 1 [file pharmaceuticals-18-00440-s001.zip › File S1/ATA_all_2025-02-21_11-51-35/Contour_Plots/steric_field_contourplot.png]

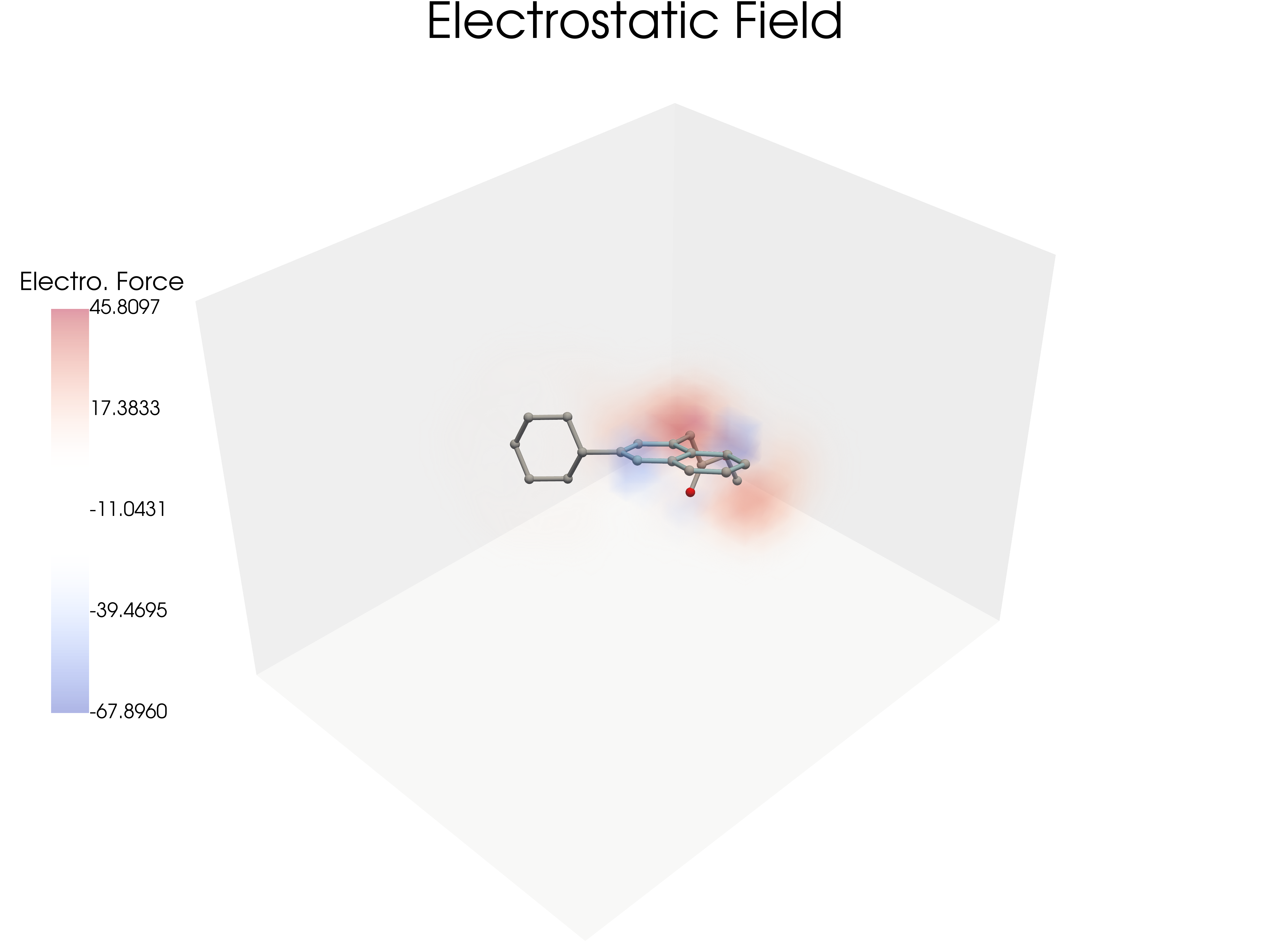

Supplement: Supplementary file 1 [file pharmaceuticals-18-00440-s001.zip › File S1/ATA_all_2025-02-21_11-51-35/Field_Plots/electrostatic.png]

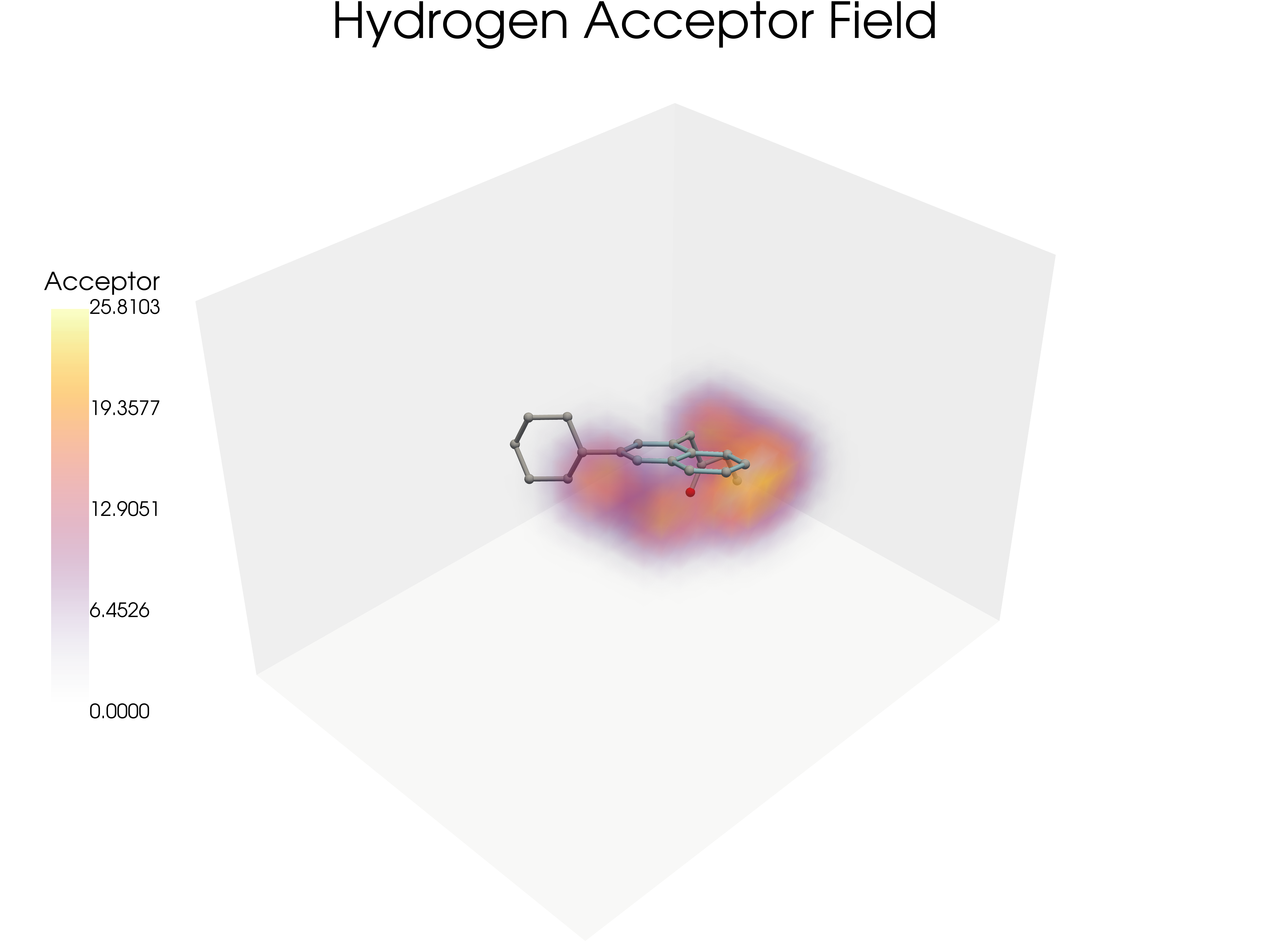

Supplement: Supplementary file 1 [file pharmaceuticals-18-00440-s001.zip › File S1/ATA_all_2025-02-21_11-51-35/Field_Plots/hbond_acceptor.png]

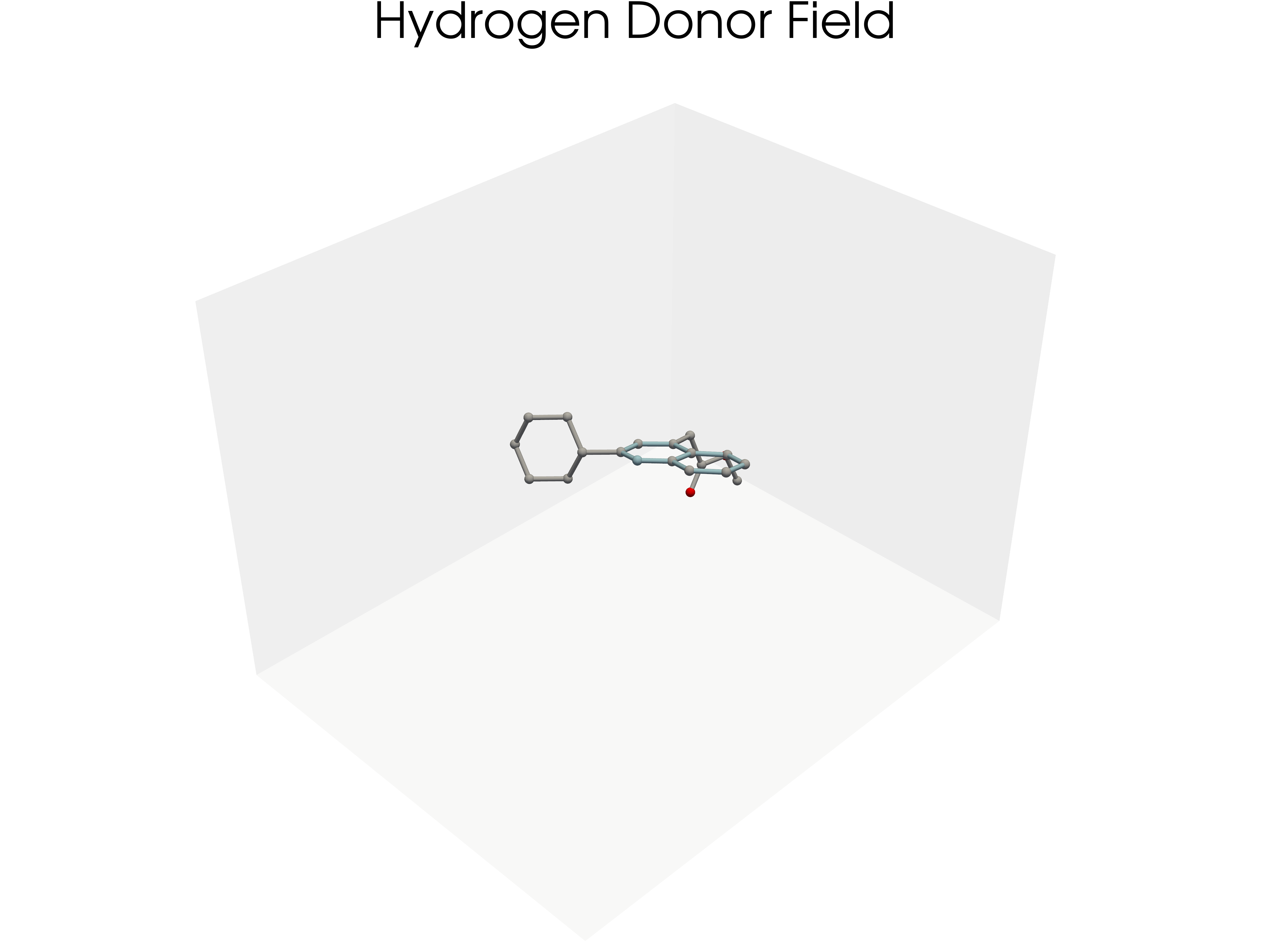

Supplement: Supplementary file 1 [file pharmaceuticals-18-00440-s001.zip › File S1/ATA_all_2025-02-21_11-51-35/Field_Plots/hbond_donor.png]

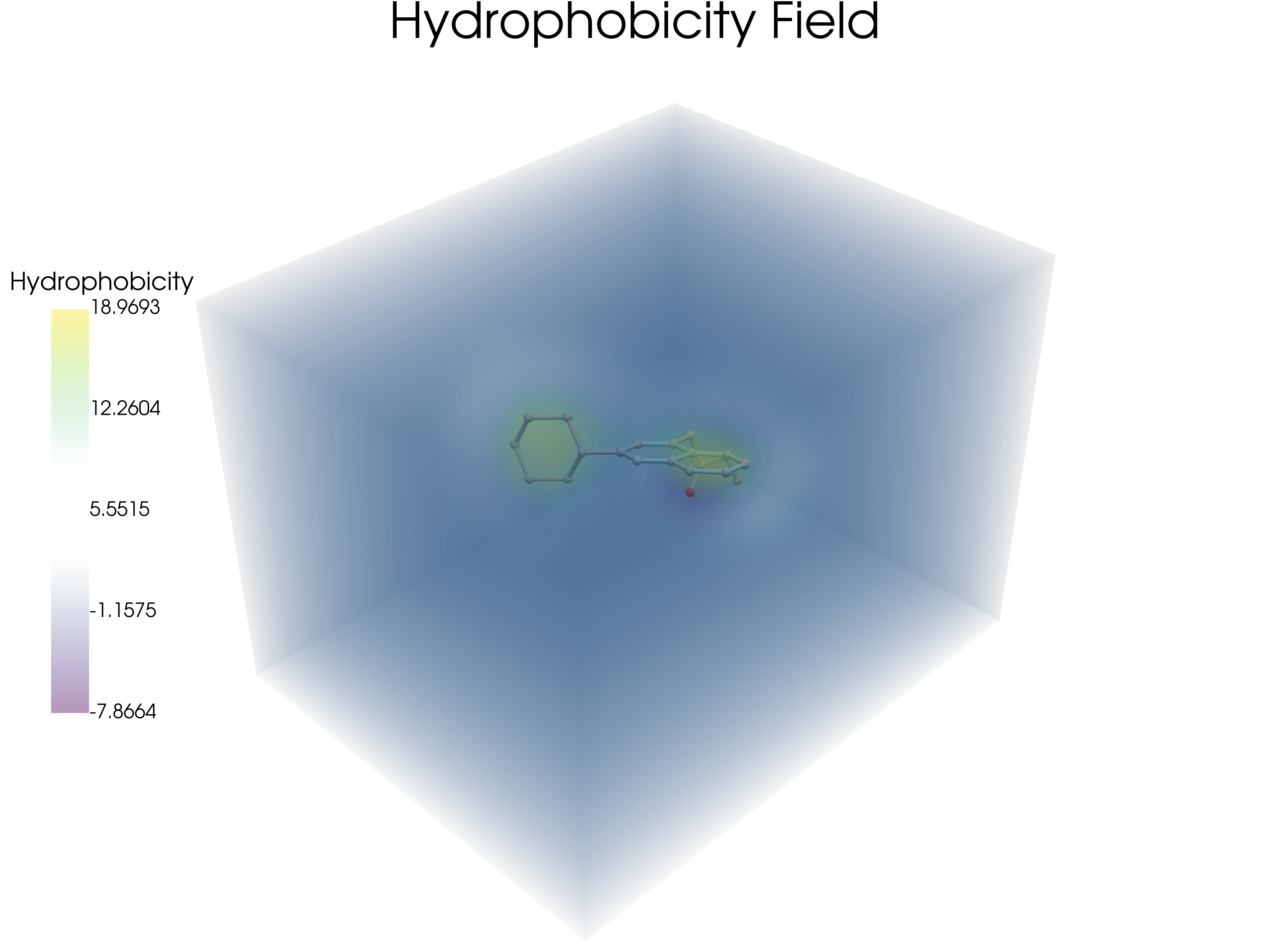

Supplement: Supplementary file 1 [file pharmaceuticals-18-00440-s001.zip › File S1/ATA_all_2025-02-21_11-51-35/Field_Plots/hydrophobic.png]

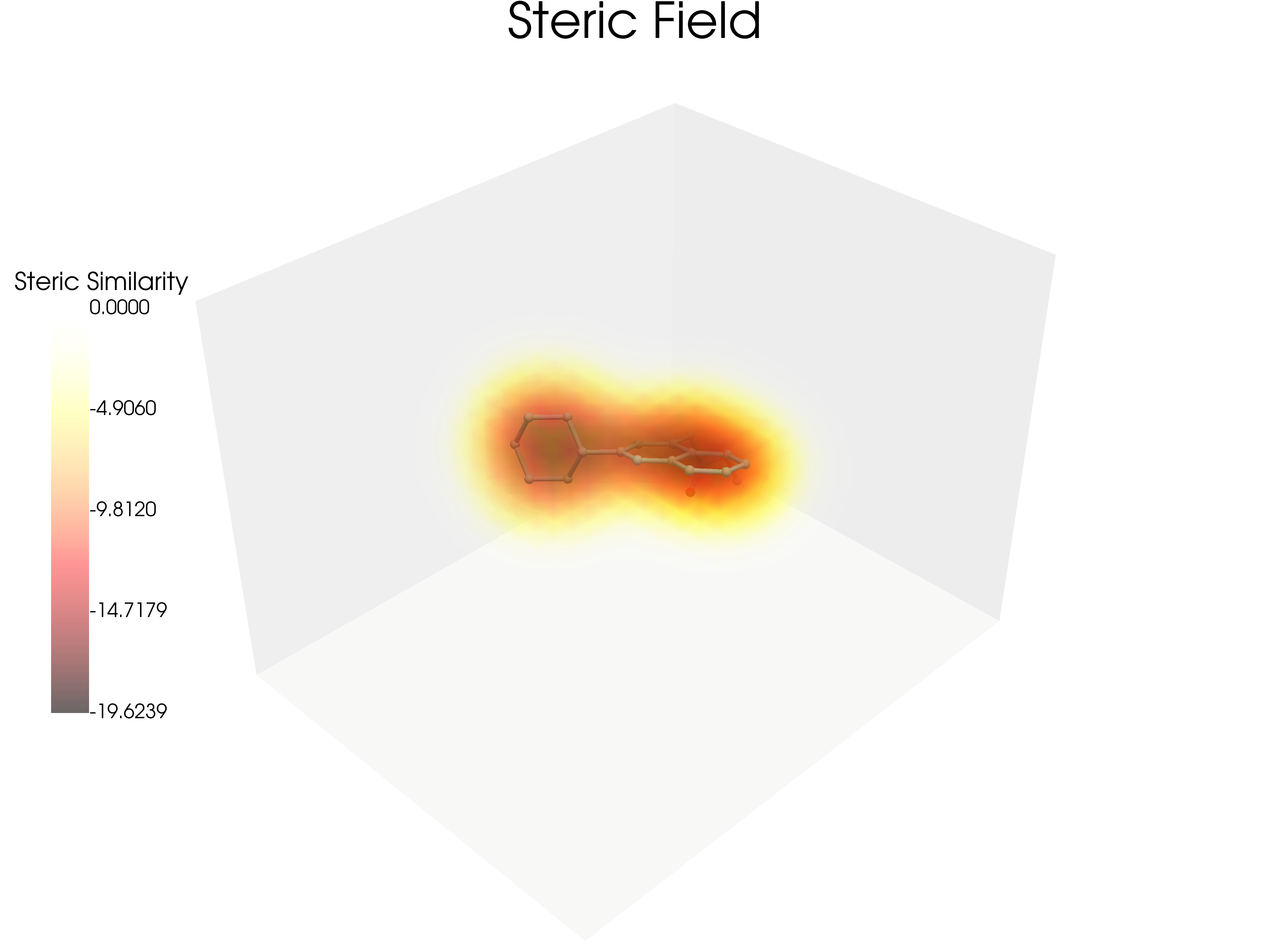

Supplement: Supplementary file 1 [file pharmaceuticals-18-00440-s001.zip › File S1/ATA_all_2025-02-21_11-51-35/Field_Plots/steric.png]

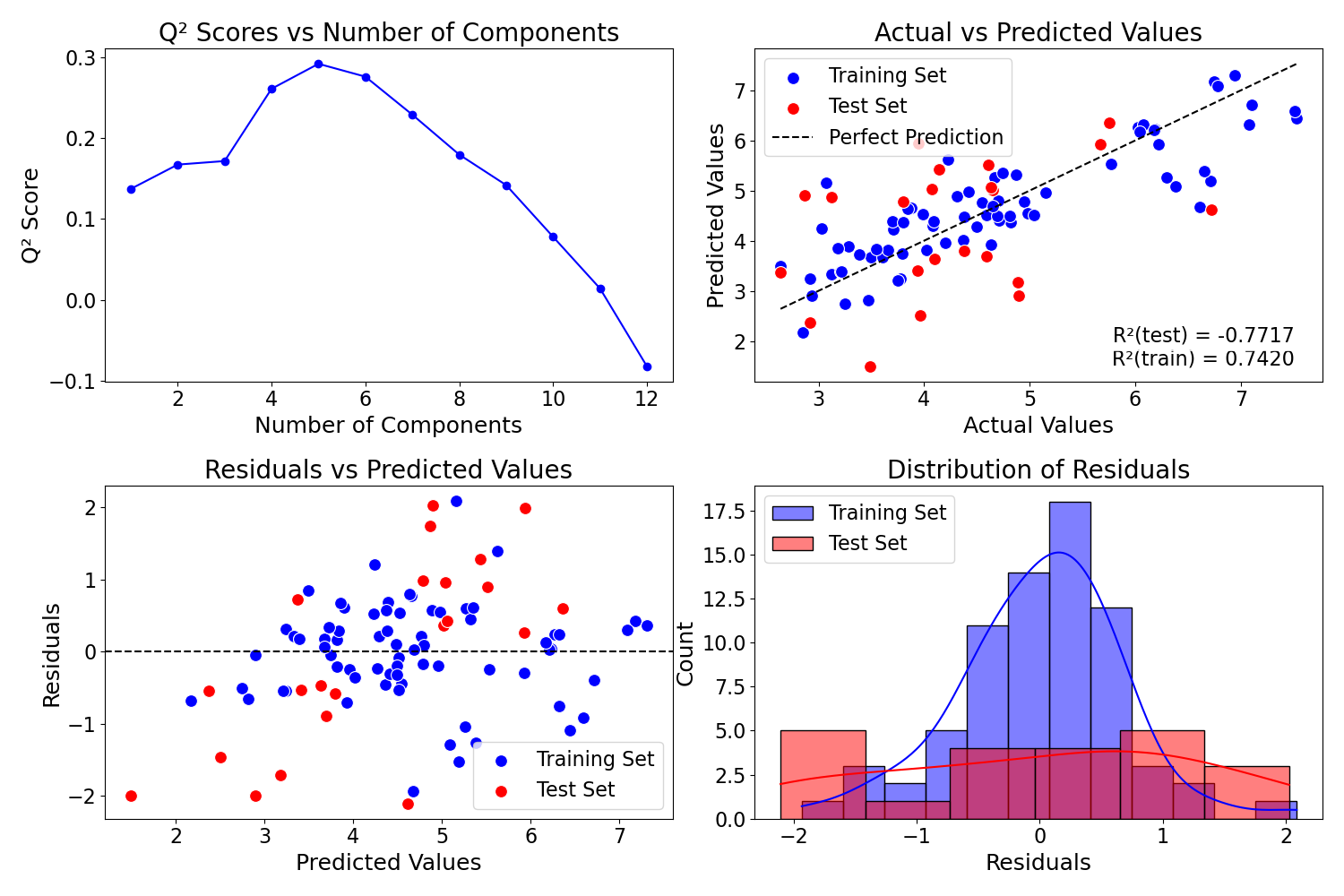

Supplement: Supplementary file 1 [file pharmaceuticals-18-00440-s001.zip › File S1/ATA_all_2025-02-21_11-51-35/PLS_Analysis/PLSplots.png]

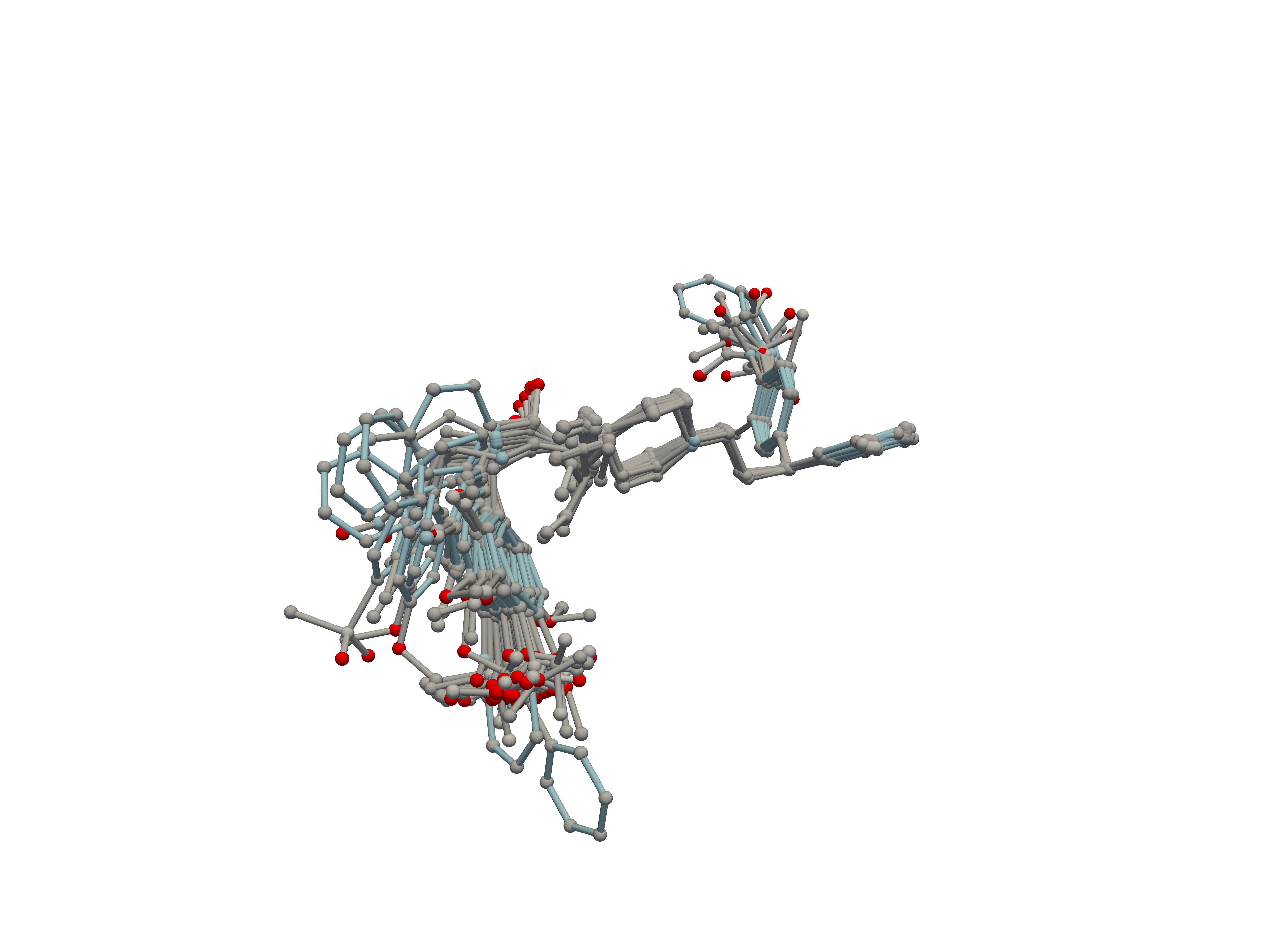

Supplement: Supplementary file 1 [file pharmaceuticals-18-00440-s001.zip › File S1/CCR5_all_2025-02-21_11-29-56/Alignments/aligned_molecules.png]

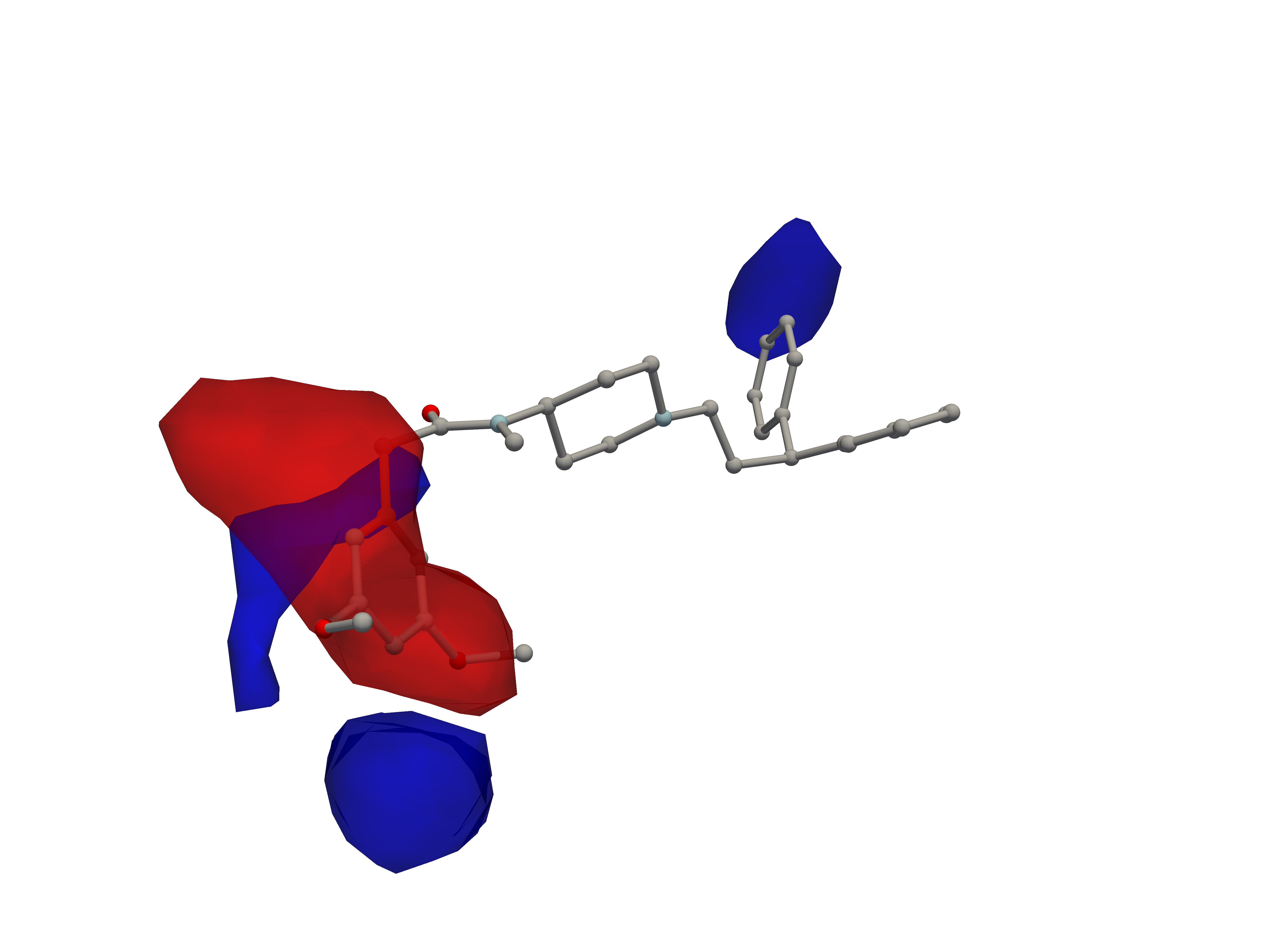

Supplement: Supplementary file 1 [file pharmaceuticals-18-00440-s001.zip › File S1/CCR5_all_2025-02-21_11-29-56/Contour_Plots/electrostatic_field_contourplot.png]

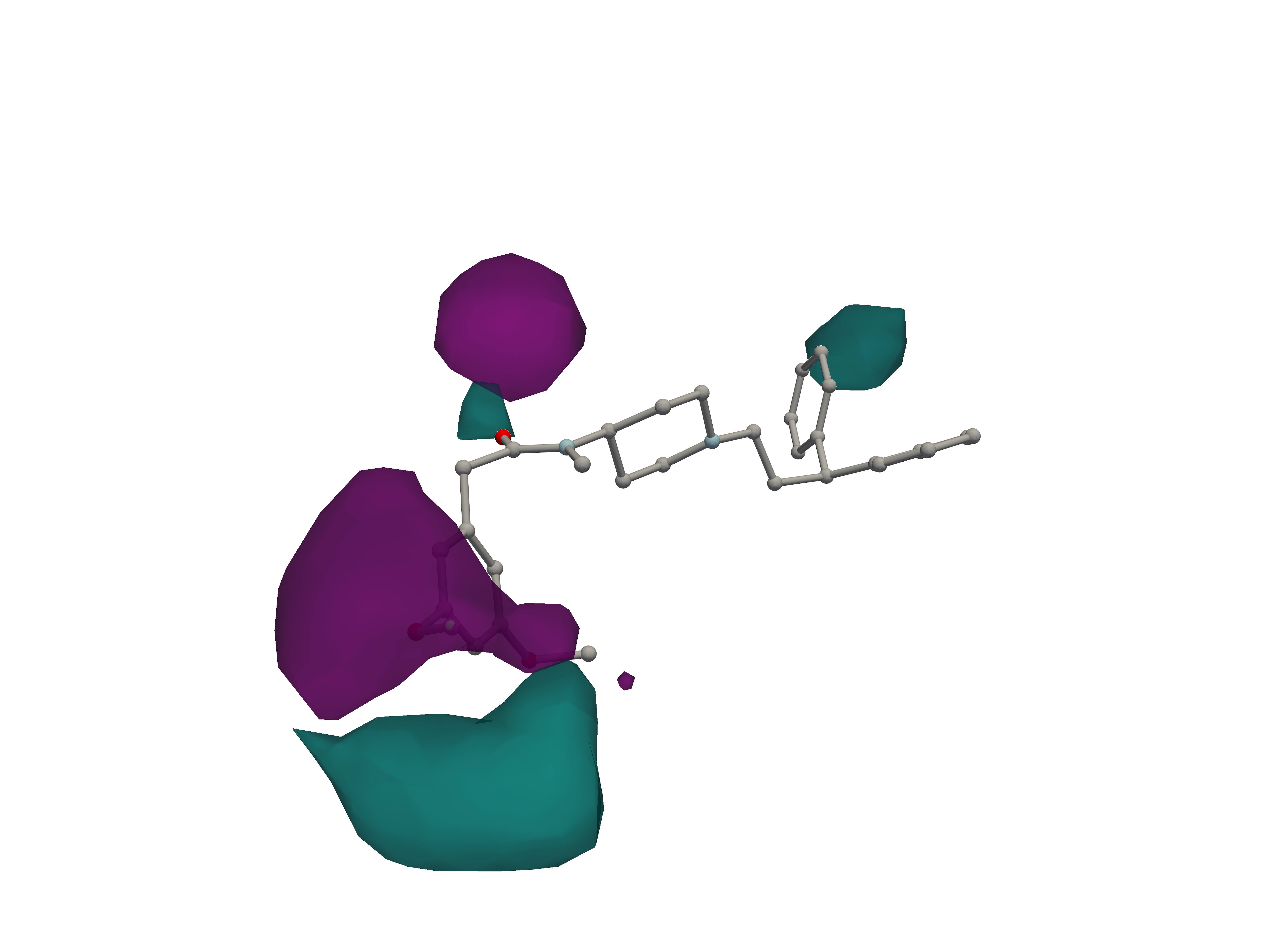

Supplement: Supplementary file 1 [file pharmaceuticals-18-00440-s001.zip › File S1/CCR5_all_2025-02-21_11-29-56/Contour_Plots/hbond_acceptor_field_contourplot.png]

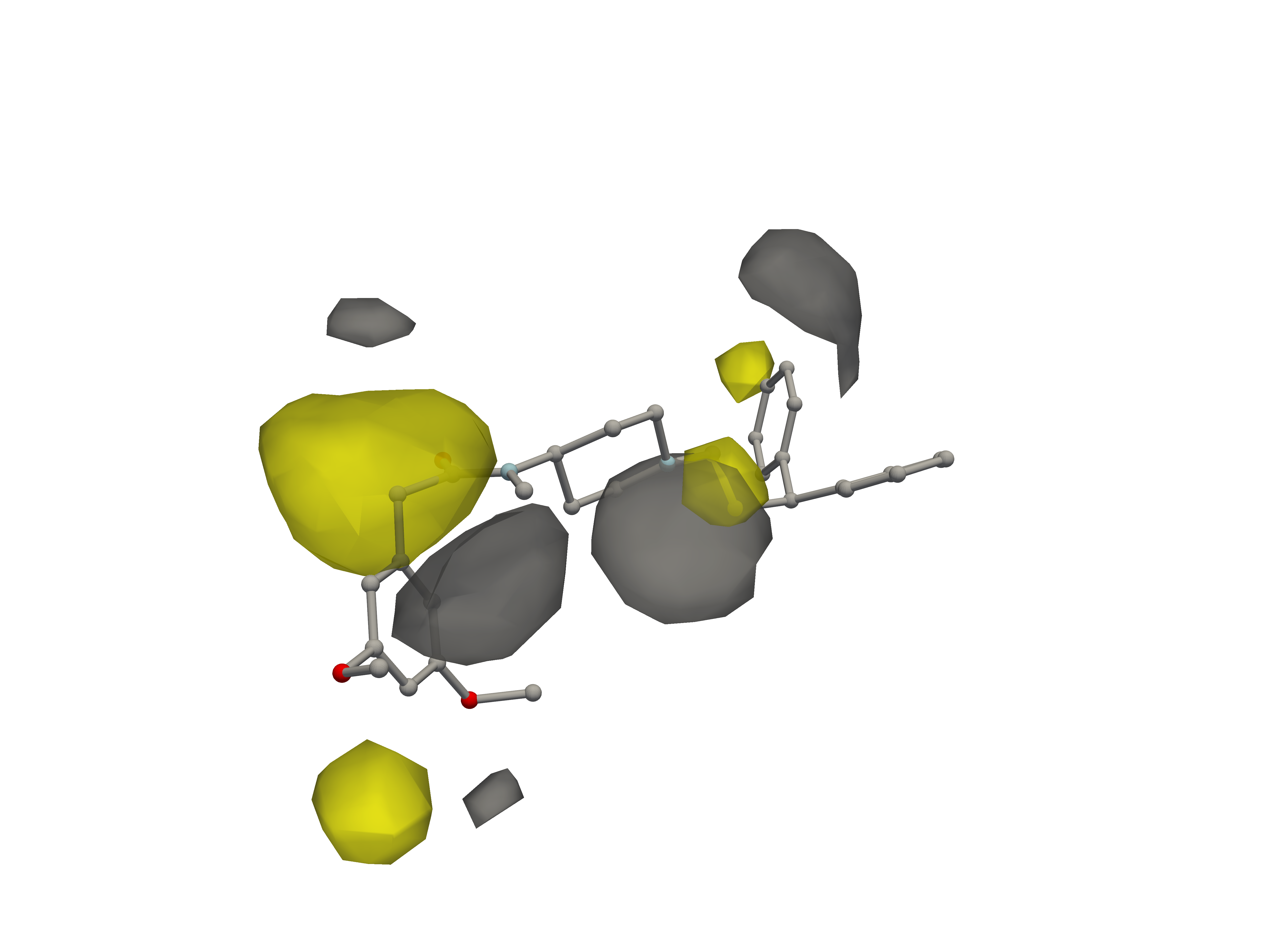

Supplement: Supplementary file 1 [file pharmaceuticals-18-00440-s001.zip › File S1/CCR5_all_2025-02-21_11-29-56/Contour_Plots/hbond_donor_field_contourplot.png]

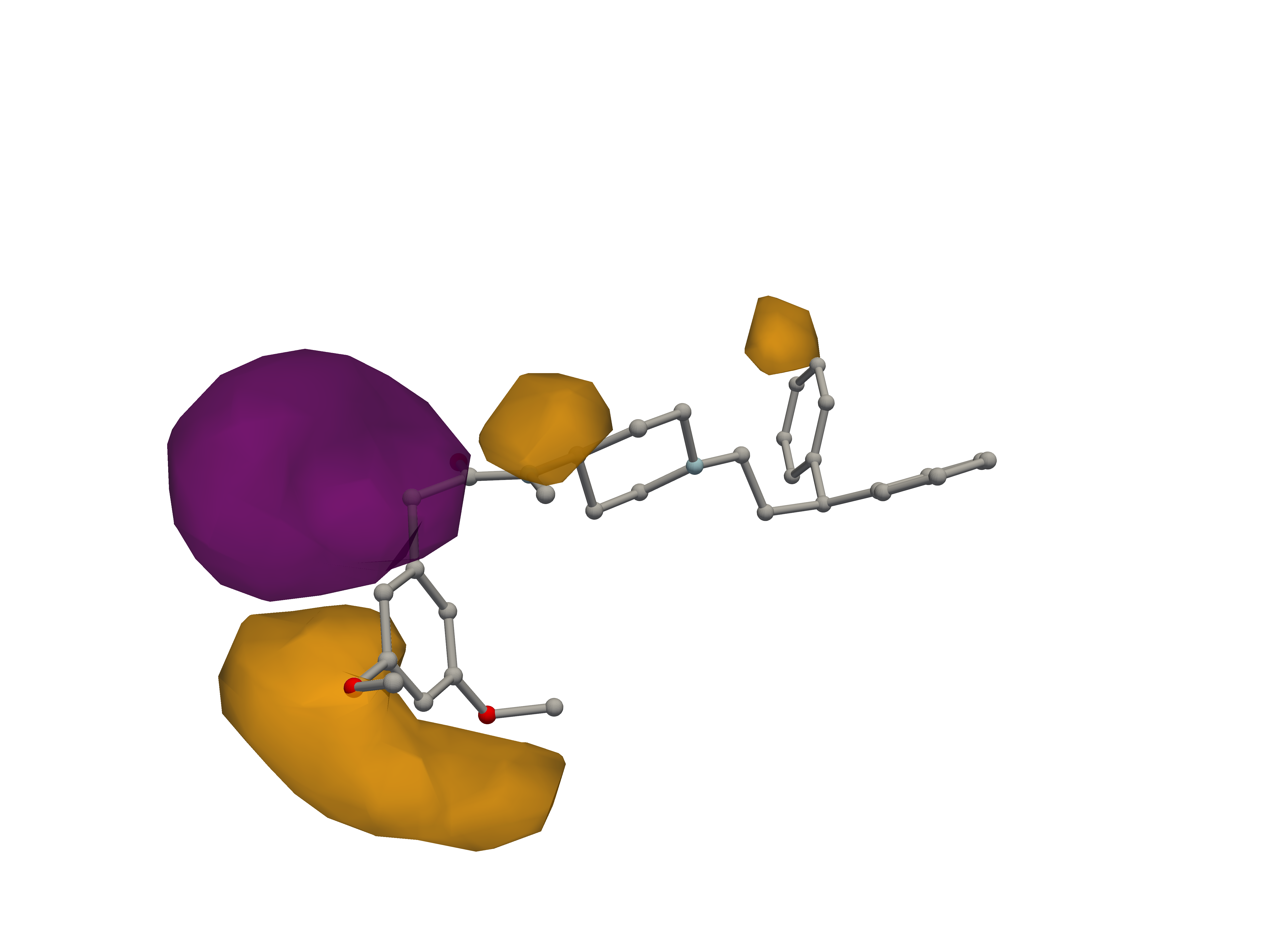

Supplement: Supplementary file 1 [file pharmaceuticals-18-00440-s001.zip › File S1/CCR5_all_2025-02-21_11-29-56/Contour_Plots/hydrophobic_field_contourplot.png]

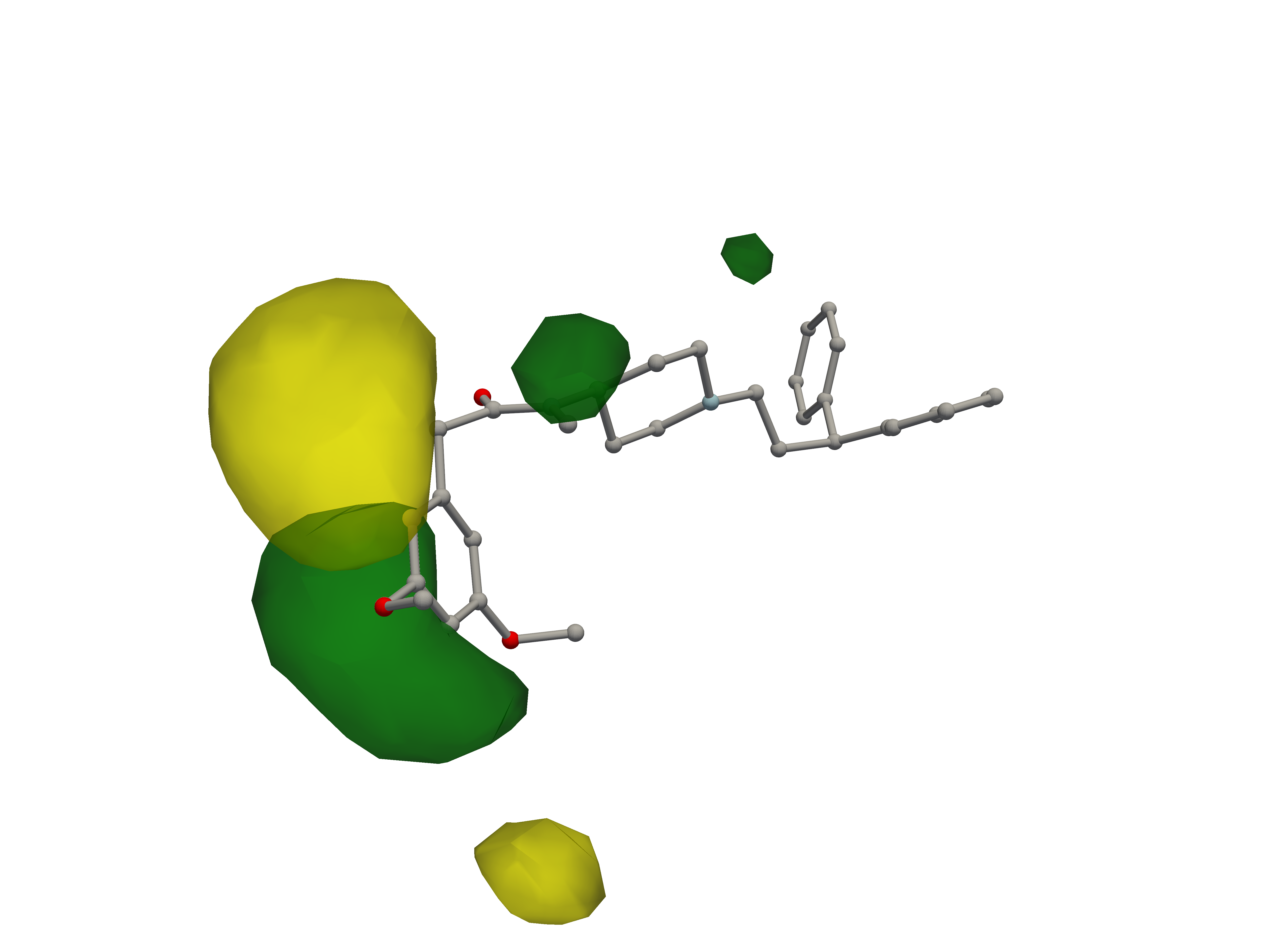

Supplement: Supplementary file 1 [file pharmaceuticals-18-00440-s001.zip › File S1/CCR5_all_2025-02-21_11-29-56/Contour_Plots/steric_field_contourplot.png]

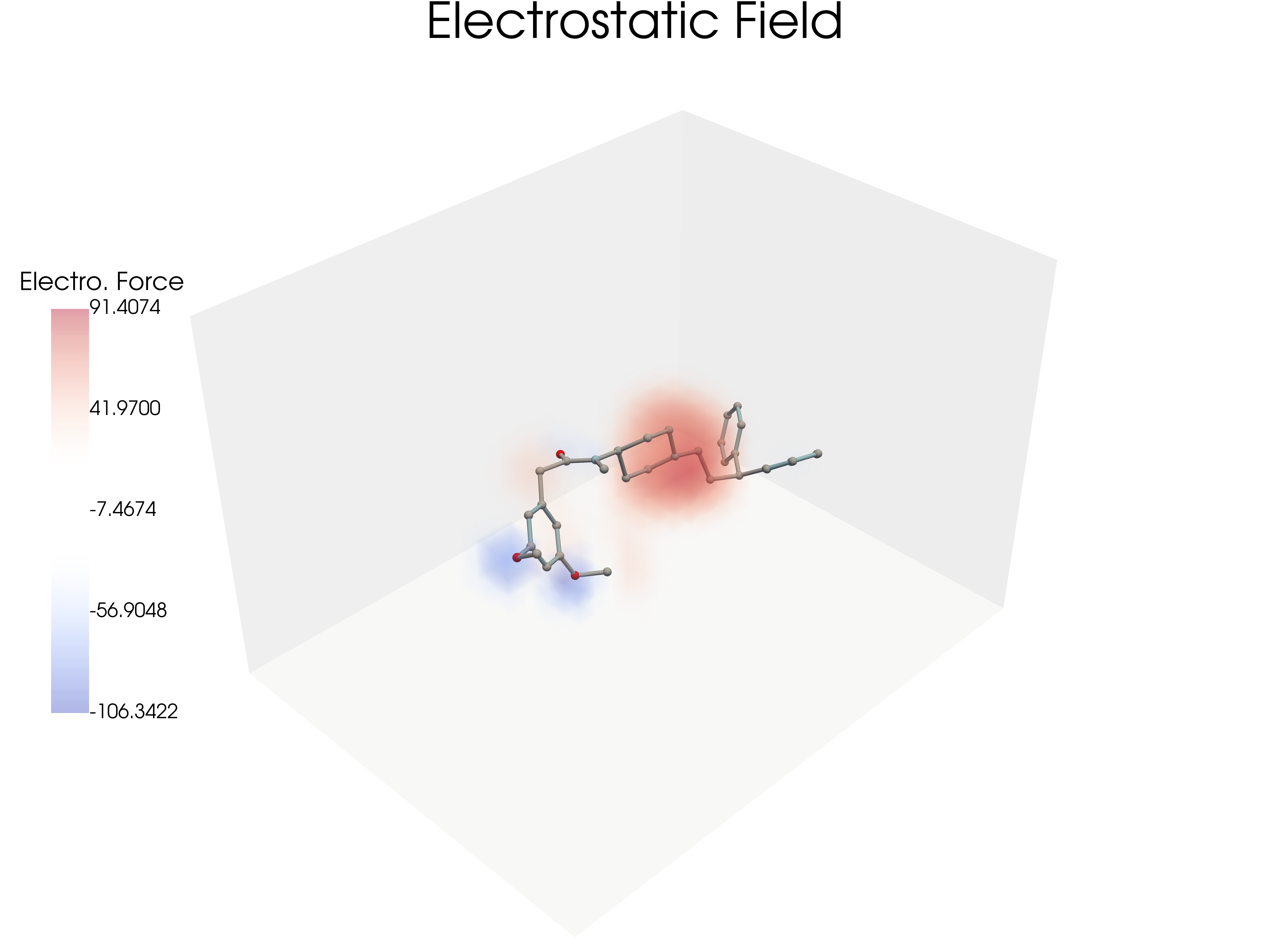

Supplement: Supplementary file 1 [file pharmaceuticals-18-00440-s001.zip › File S1/CCR5_all_2025-02-21_11-29-56/Field_Plots/electrostatic.png]

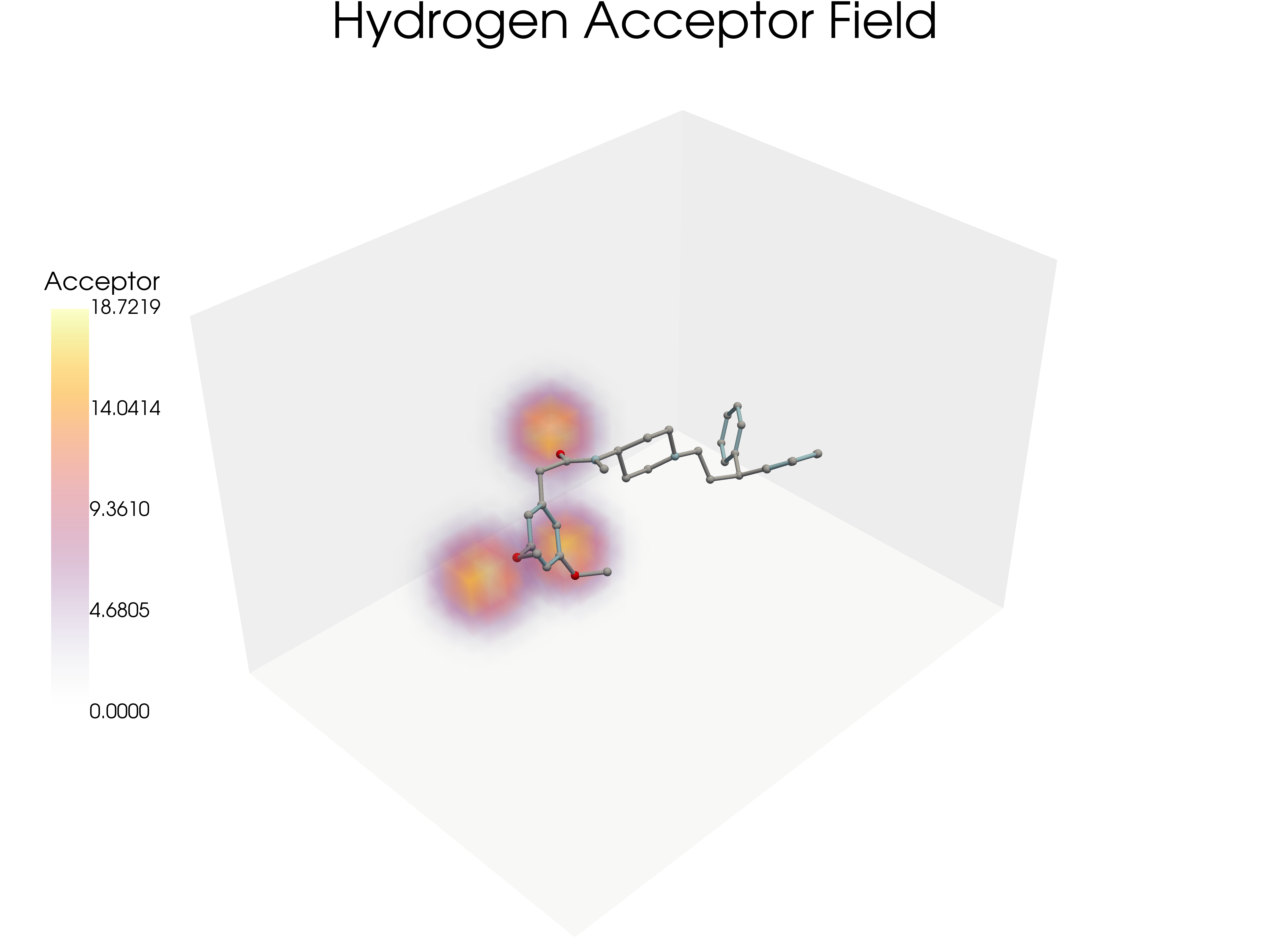

Supplement: Supplementary file 1 [file pharmaceuticals-18-00440-s001.zip › File S1/CCR5_all_2025-02-21_11-29-56/Field_Plots/hbond_acceptor.png]

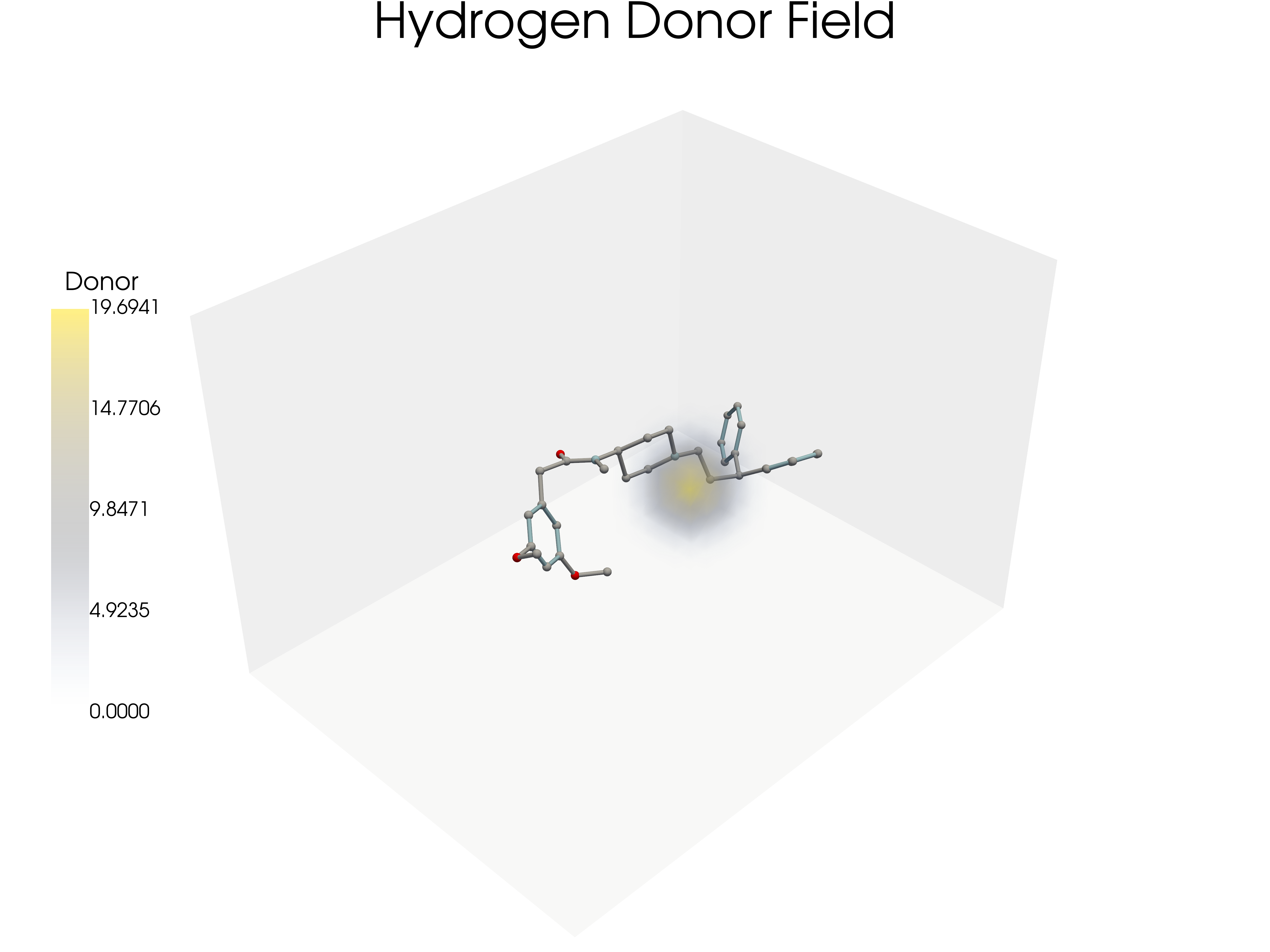

Supplement: Supplementary file 1 [file pharmaceuticals-18-00440-s001.zip › File S1/CCR5_all_2025-02-21_11-29-56/Field_Plots/hbond_donor.png]

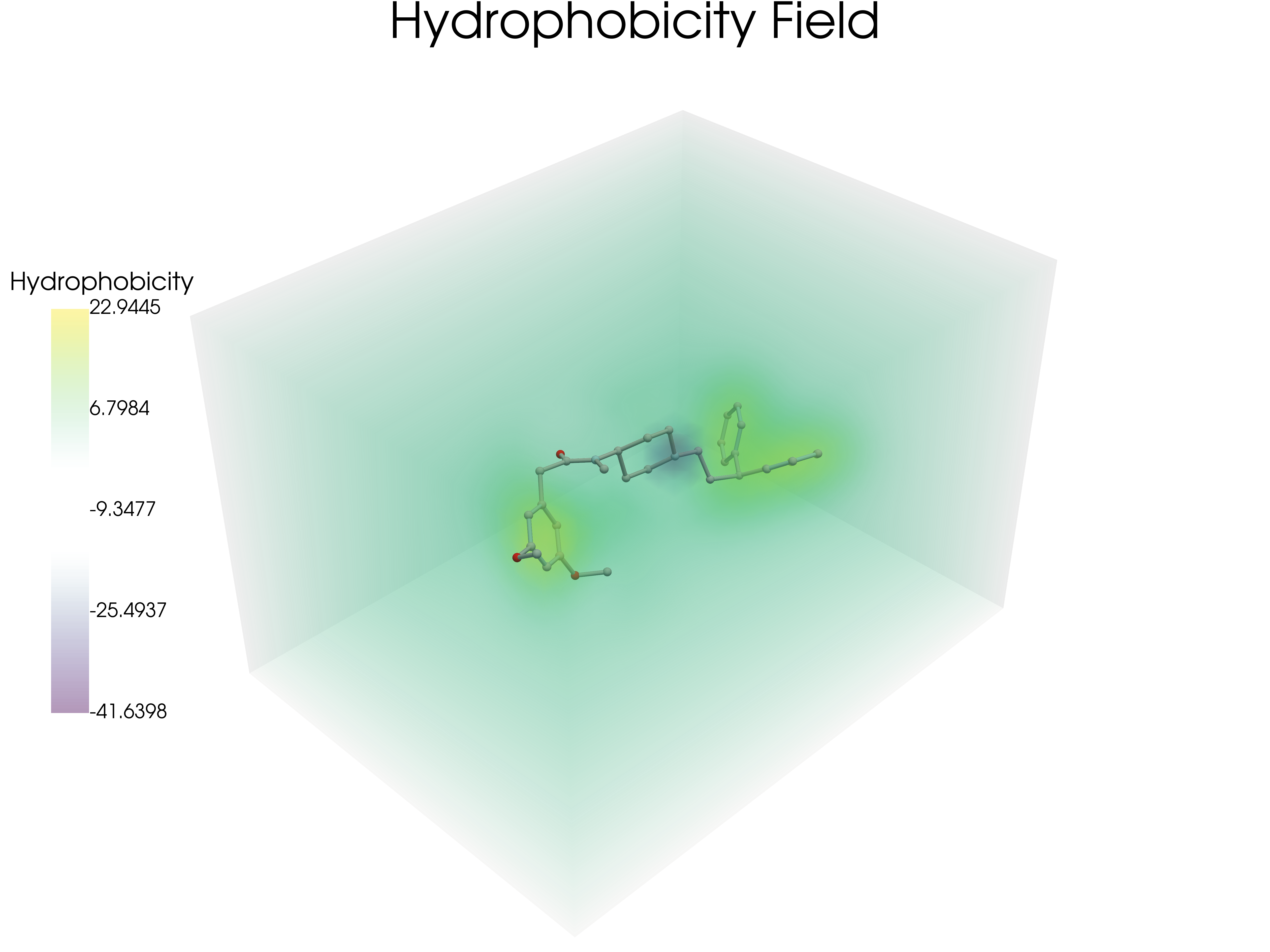

Supplement: Supplementary file 1 [file pharmaceuticals-18-00440-s001.zip › File S1/CCR5_all_2025-02-21_11-29-56/Field_Plots/hydrophobic.png]

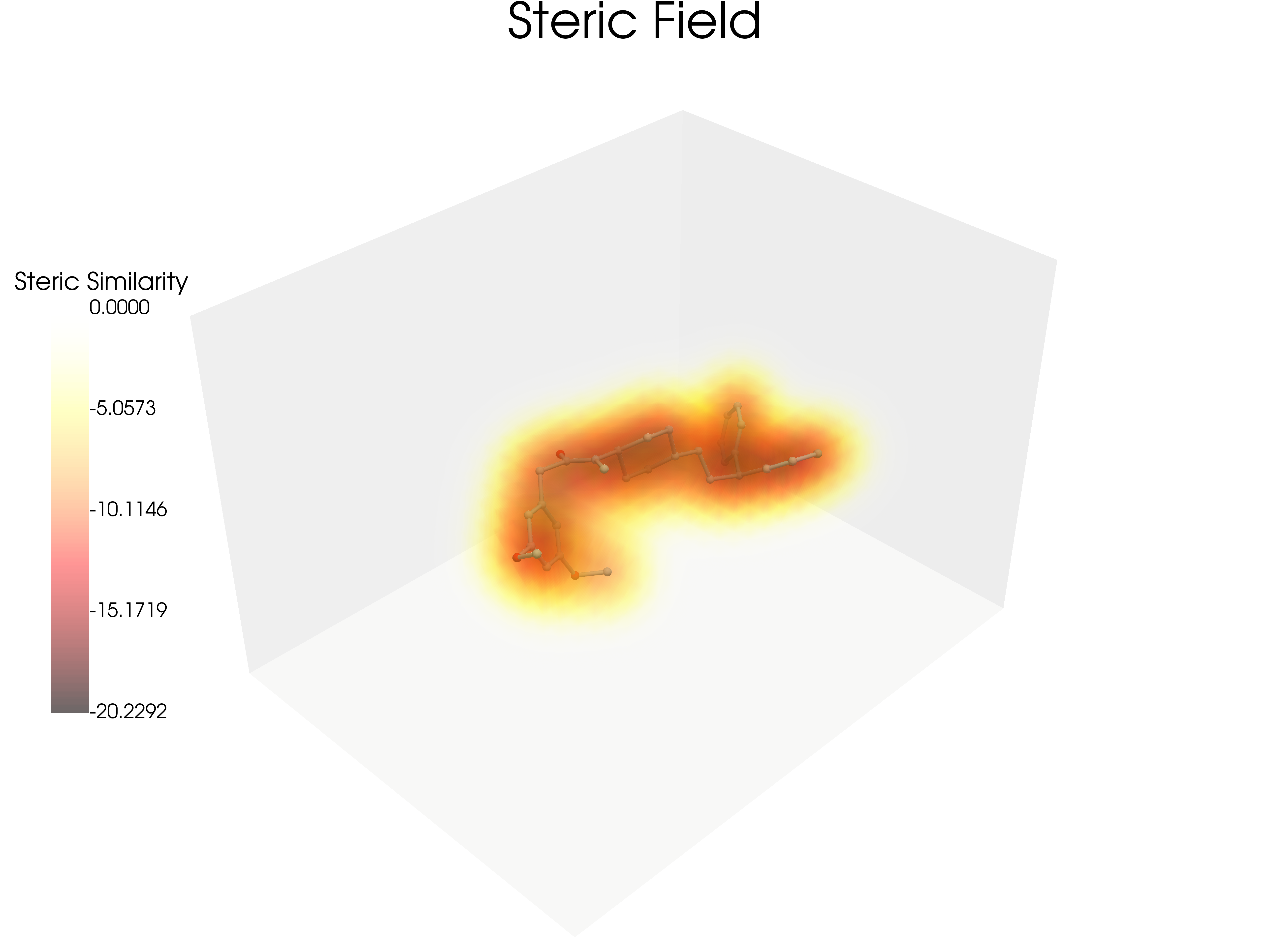

Supplement: Supplementary file 1 [file pharmaceuticals-18-00440-s001.zip › File S1/CCR5_all_2025-02-21_11-29-56/Field_Plots/steric.png]

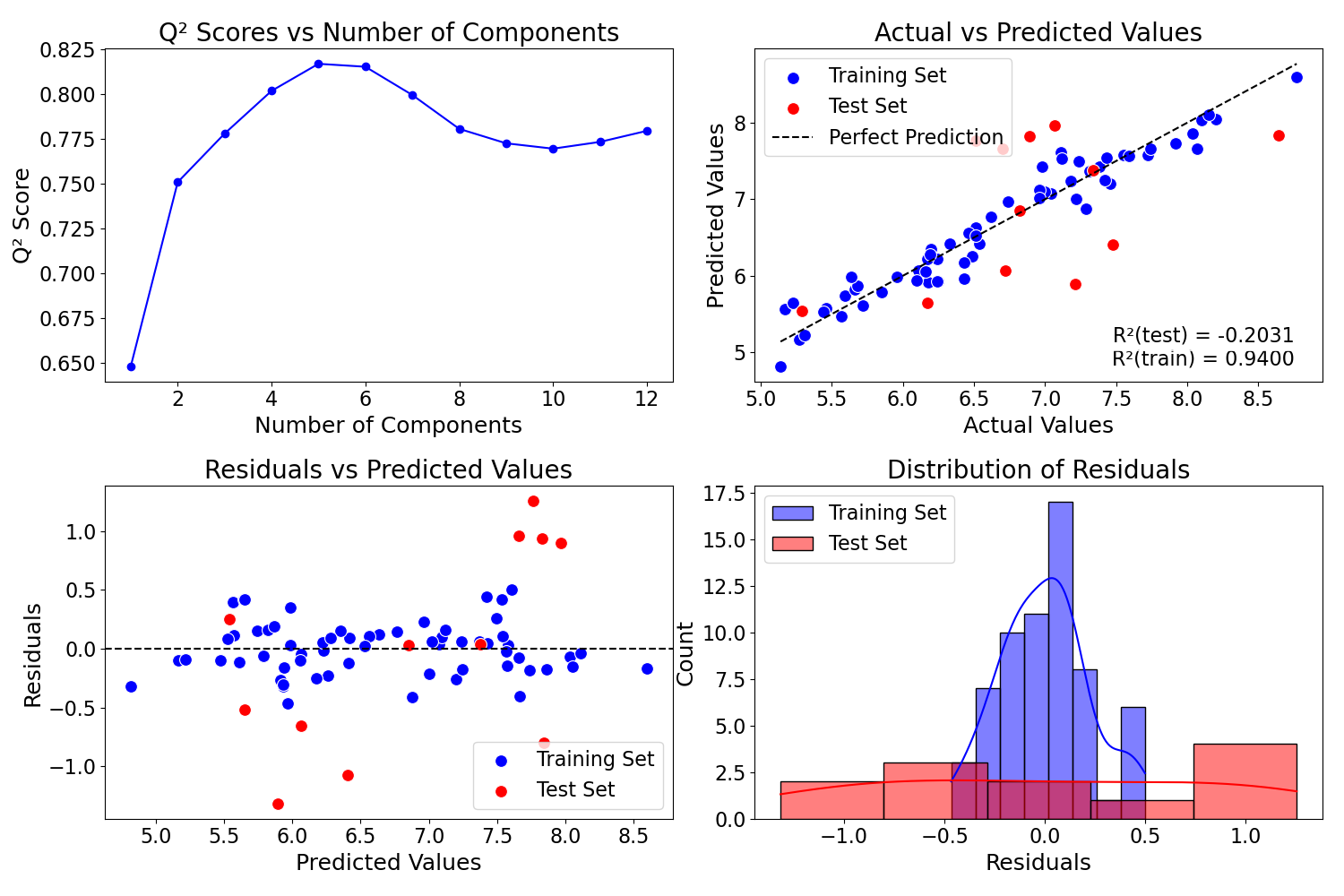

Supplement: Supplementary file 1 [file pharmaceuticals-18-00440-s001.zip › File S1/CCR5_all_2025-02-21_11-29-56/PLS_Analysis/PLSplots.png]

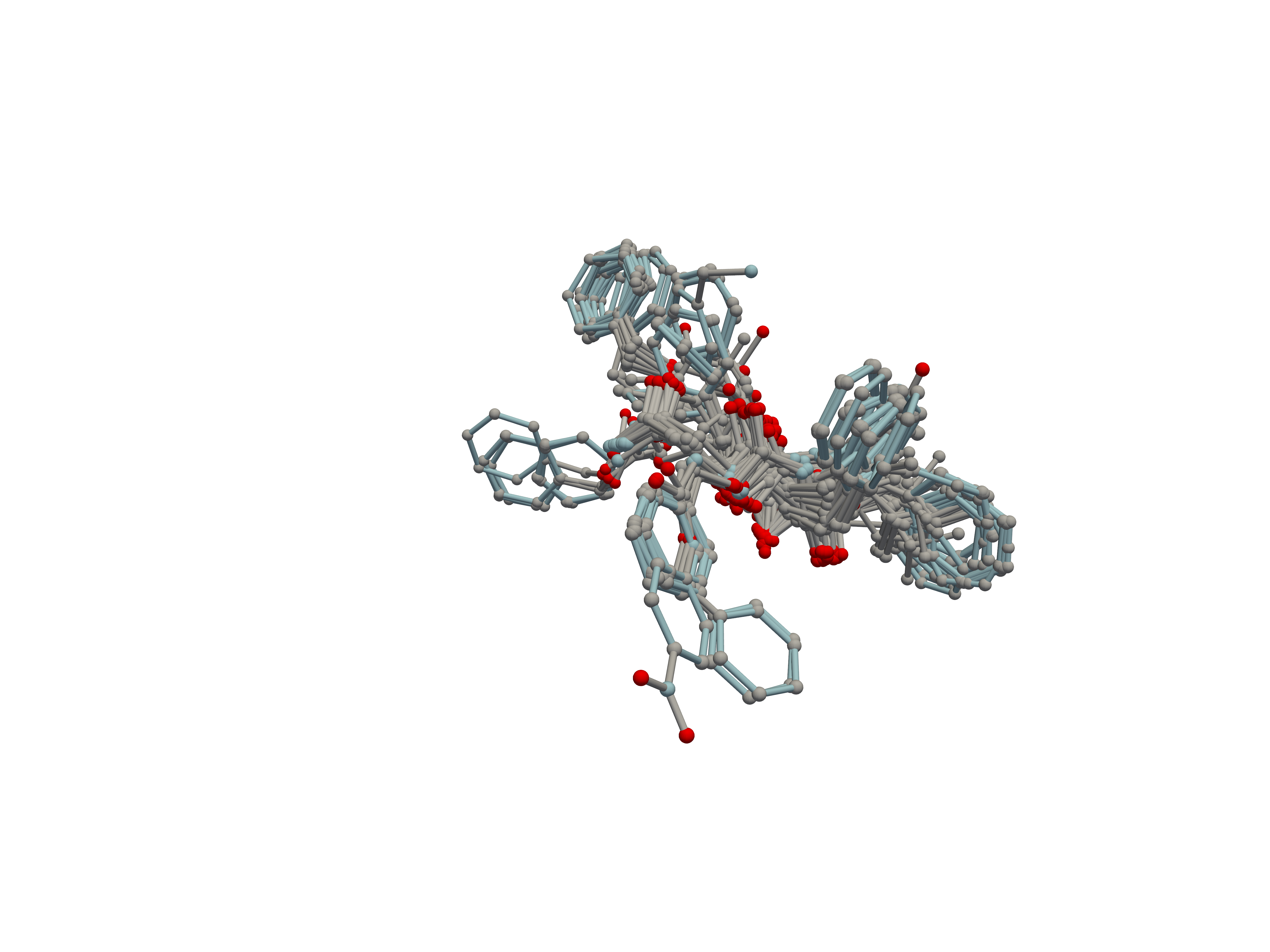

Supplement: Supplementary file 1 [file pharmaceuticals-18-00440-s001.zip › File S1/THERM_SEAD_2025-02-21_12-03-41/Alignments/aligned_molecules.png]

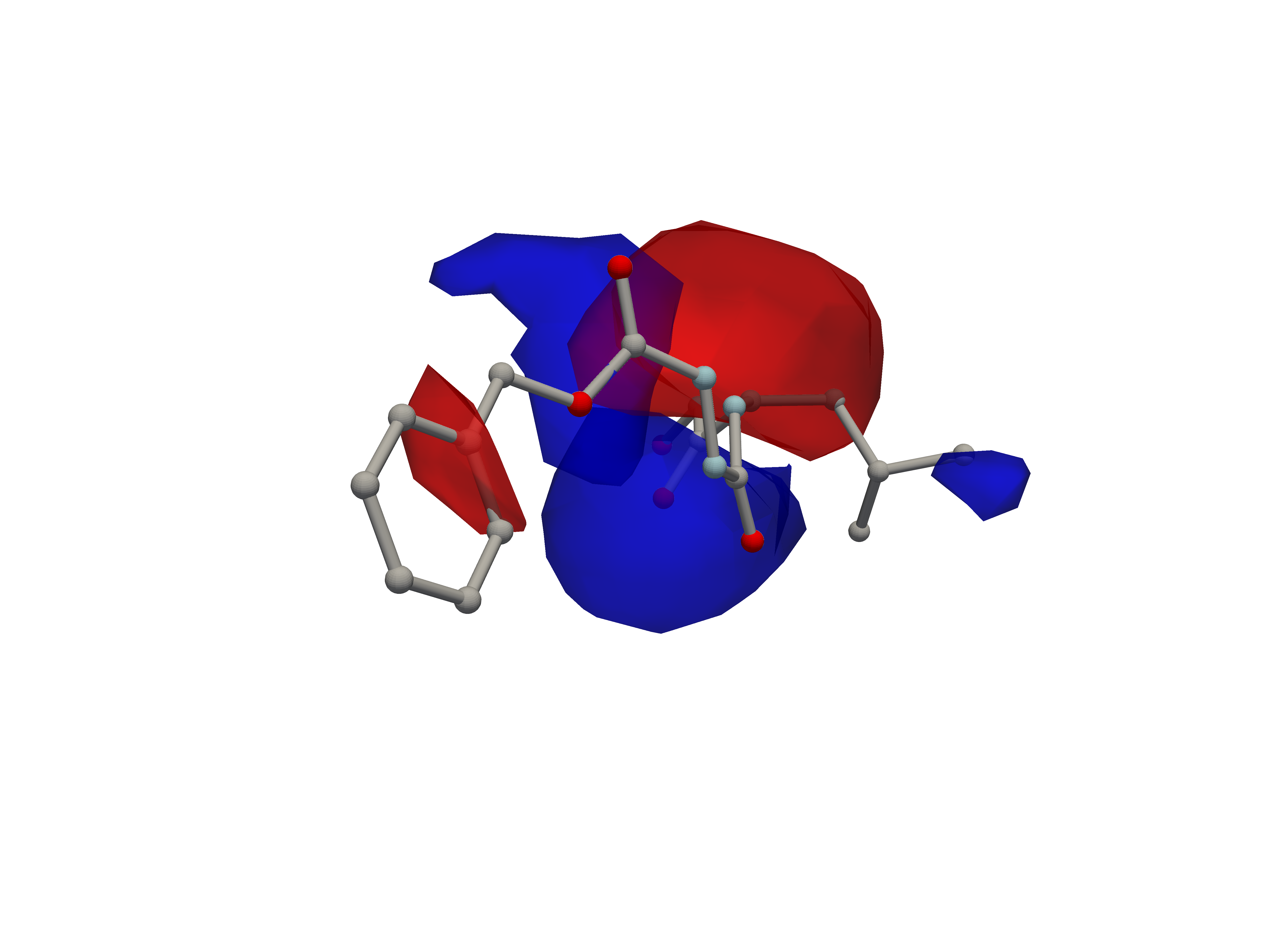

Supplement: Supplementary file 1 [file pharmaceuticals-18-00440-s001.zip › File S1/THERM_SEAD_2025-02-21_12-03-41/Contour_Plots/electrostatic_field_contourplot.png]

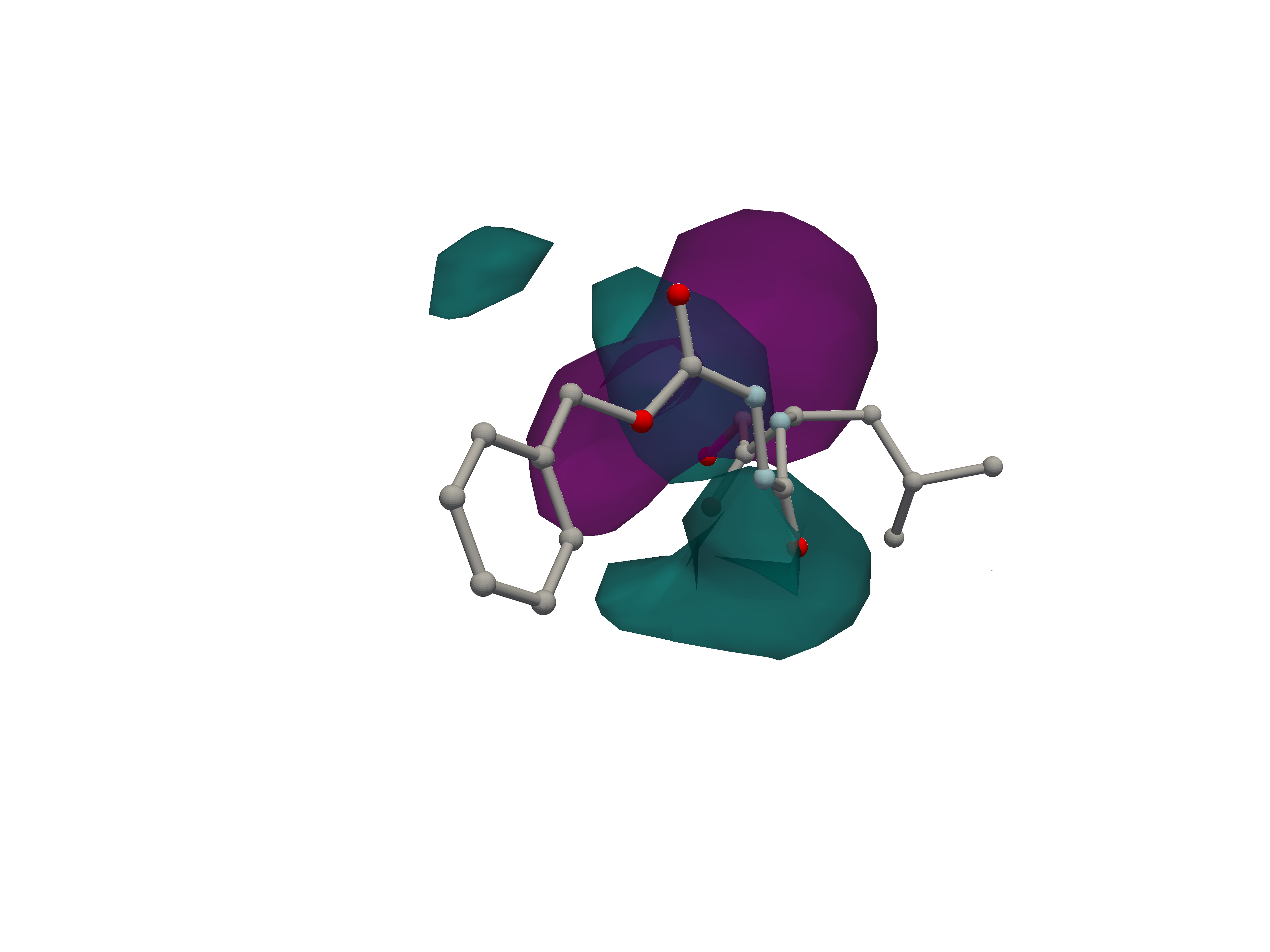

Supplement: Supplementary file 1 [file pharmaceuticals-18-00440-s001.zip › File S1/THERM_SEAD_2025-02-21_12-03-41/Contour_Plots/hbond_acceptor_field_contourplot.png]

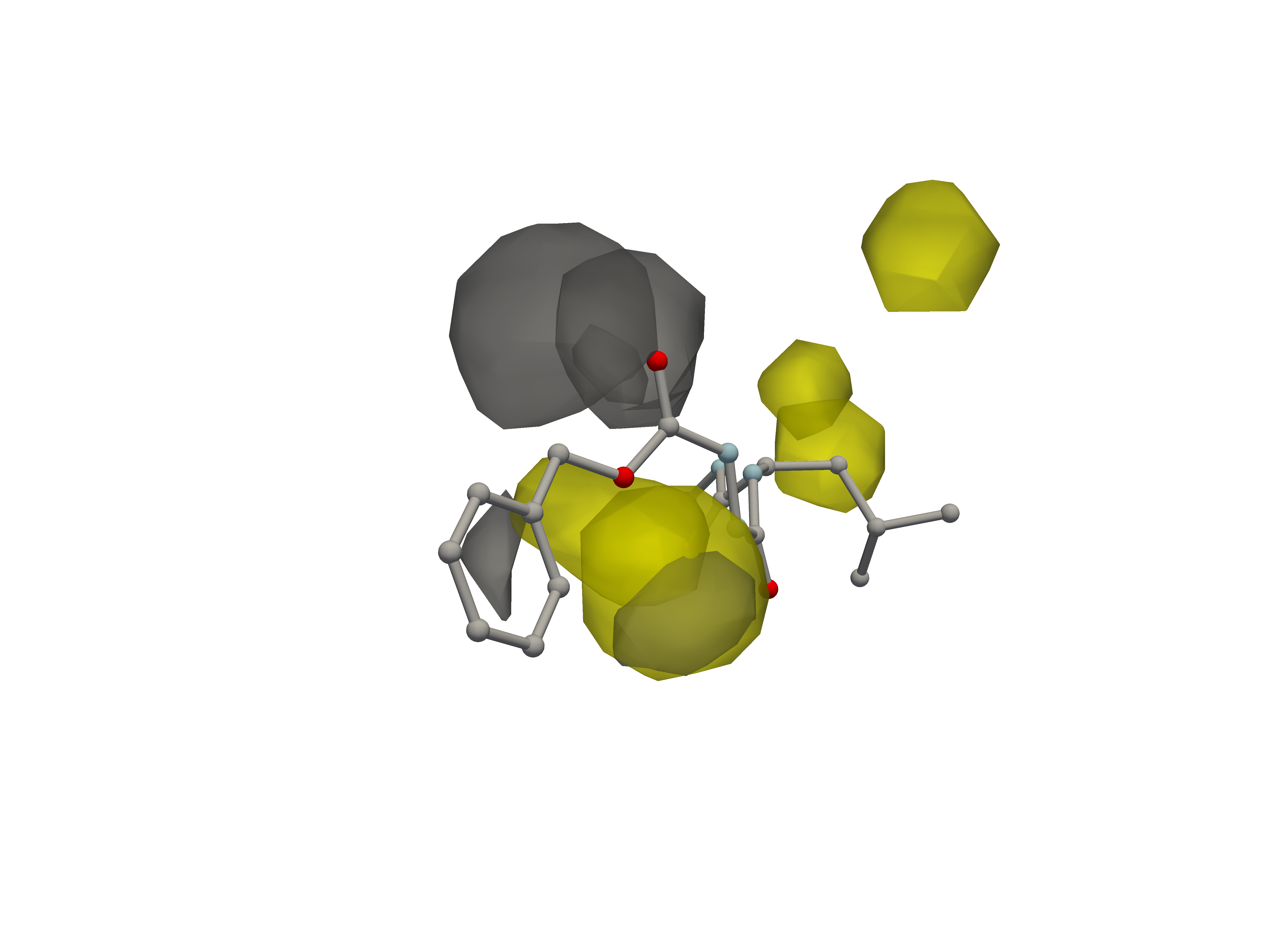

Supplement: Supplementary file 1 [file pharmaceuticals-18-00440-s001.zip › File S1/THERM_SEAD_2025-02-21_12-03-41/Contour_Plots/hbond_donor_field_contourplot.png]

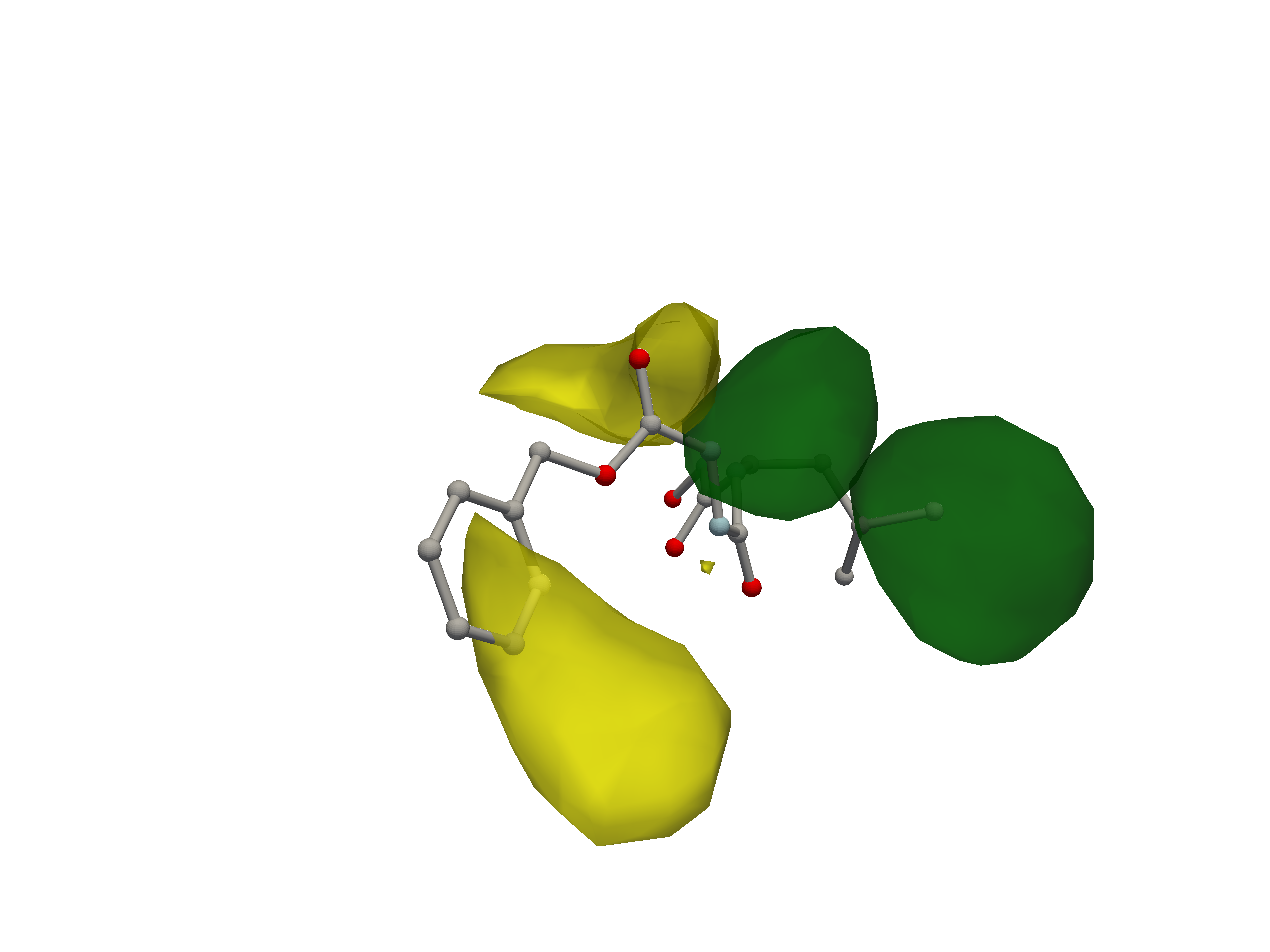

Supplement: Supplementary file 1 [file pharmaceuticals-18-00440-s001.zip › File S1/THERM_SEAD_2025-02-21_12-03-41/Contour_Plots/steric_field_contourplot.png]

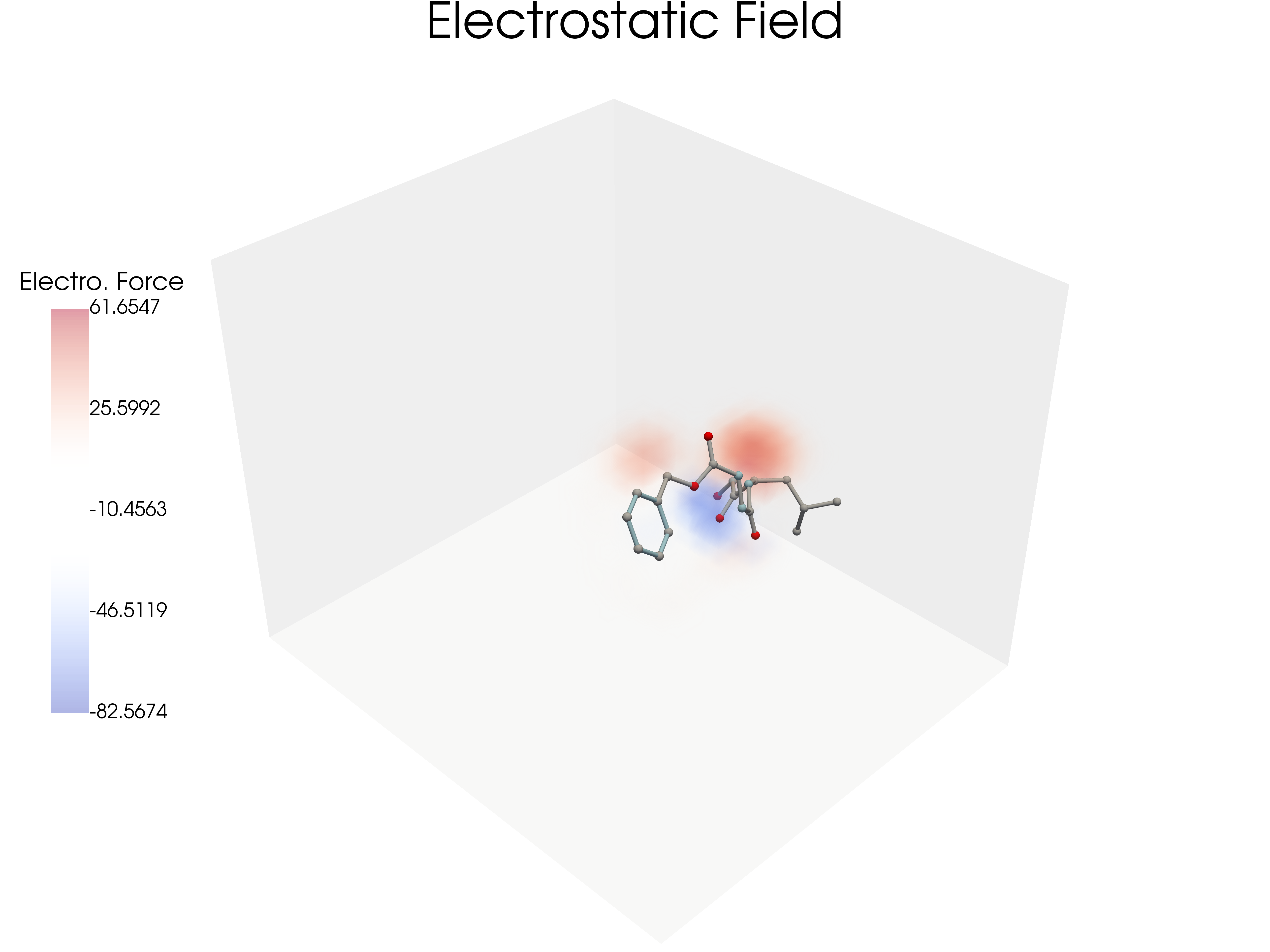

Supplement: Supplementary file 1 [file pharmaceuticals-18-00440-s001.zip › File S1/THERM_SEAD_2025-02-21_12-03-41/Field_Plots/electrostatic.png]

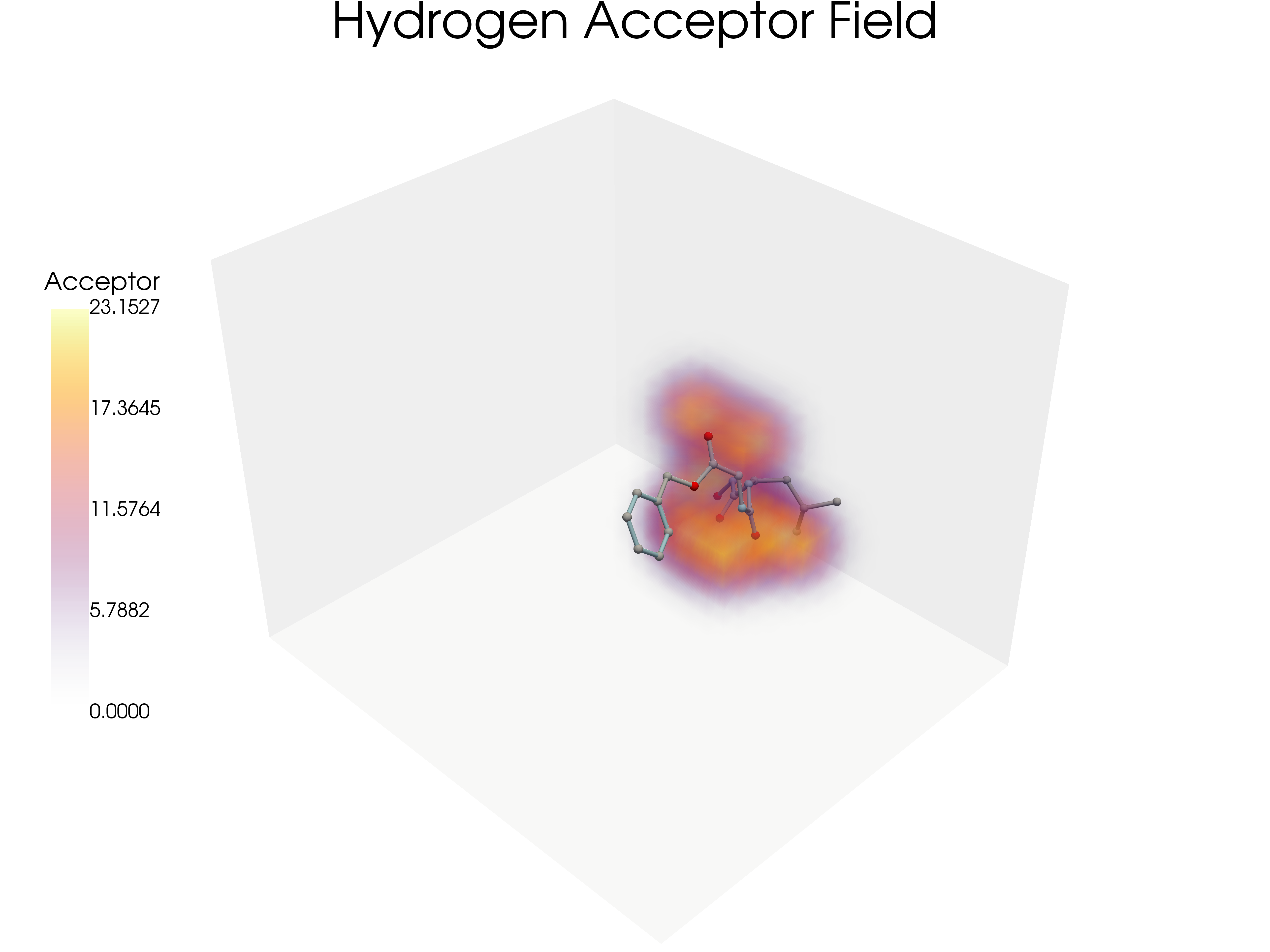

Supplement: Supplementary file 1 [file pharmaceuticals-18-00440-s001.zip › File S1/THERM_SEAD_2025-02-21_12-03-41/Field_Plots/hbond_acceptor.png]

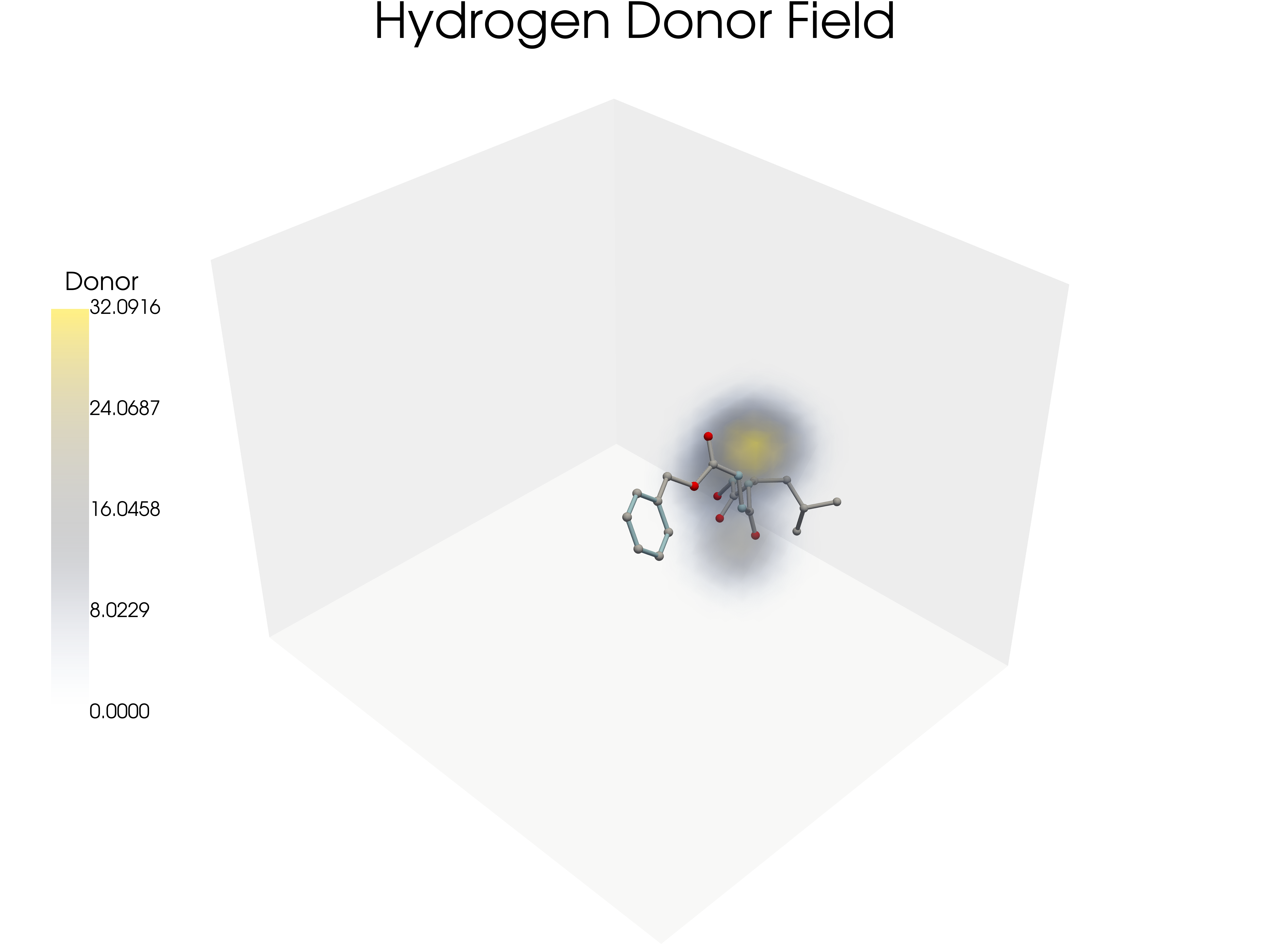

Supplement: Supplementary file 1 [file pharmaceuticals-18-00440-s001.zip › File S1/THERM_SEAD_2025-02-21_12-03-41/Field_Plots/hbond_donor.png]

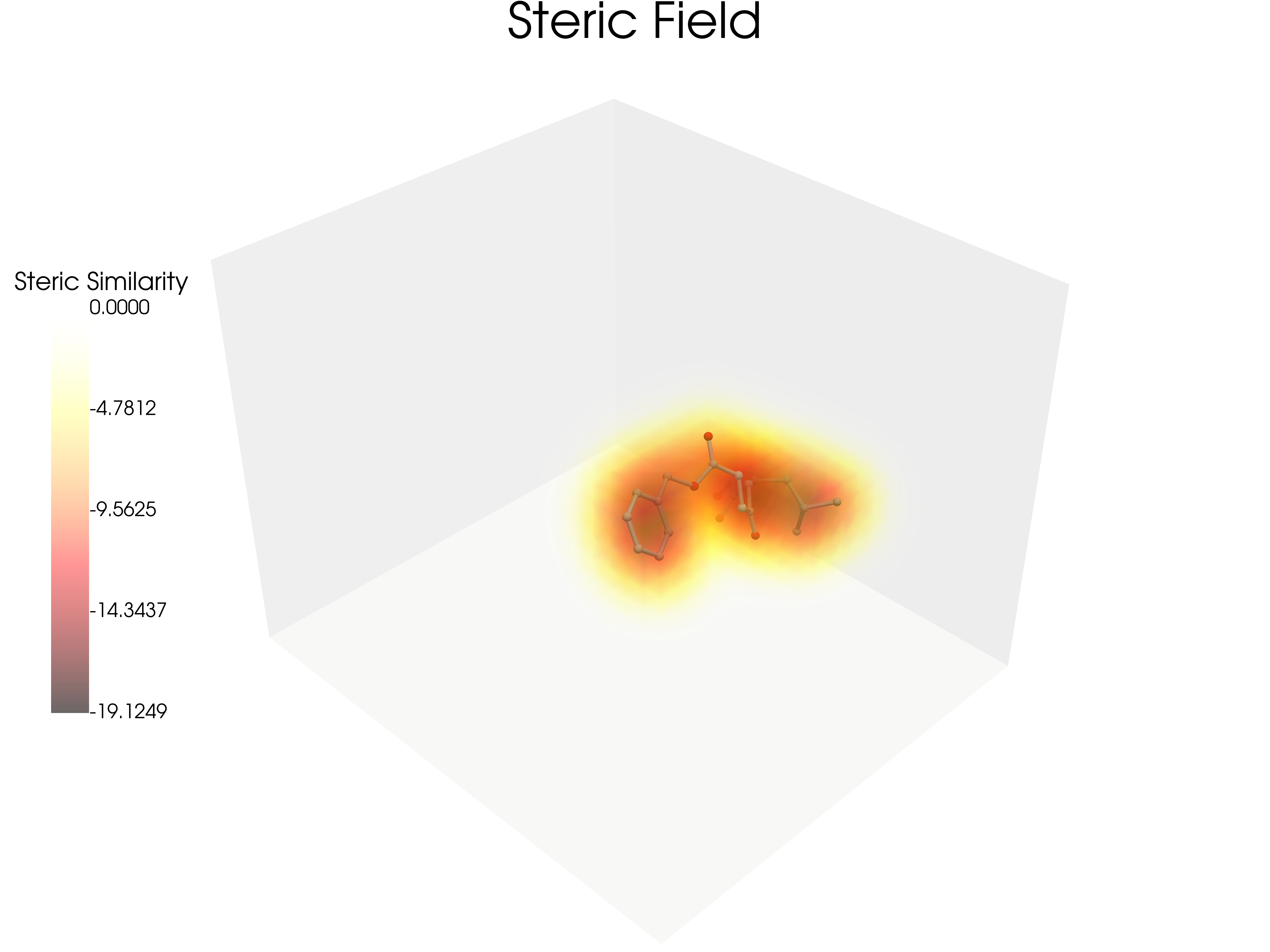

Supplement: Supplementary file 1 [file pharmaceuticals-18-00440-s001.zip › File S1/THERM_SEAD_2025-02-21_12-03-41/Field_Plots/steric.png]

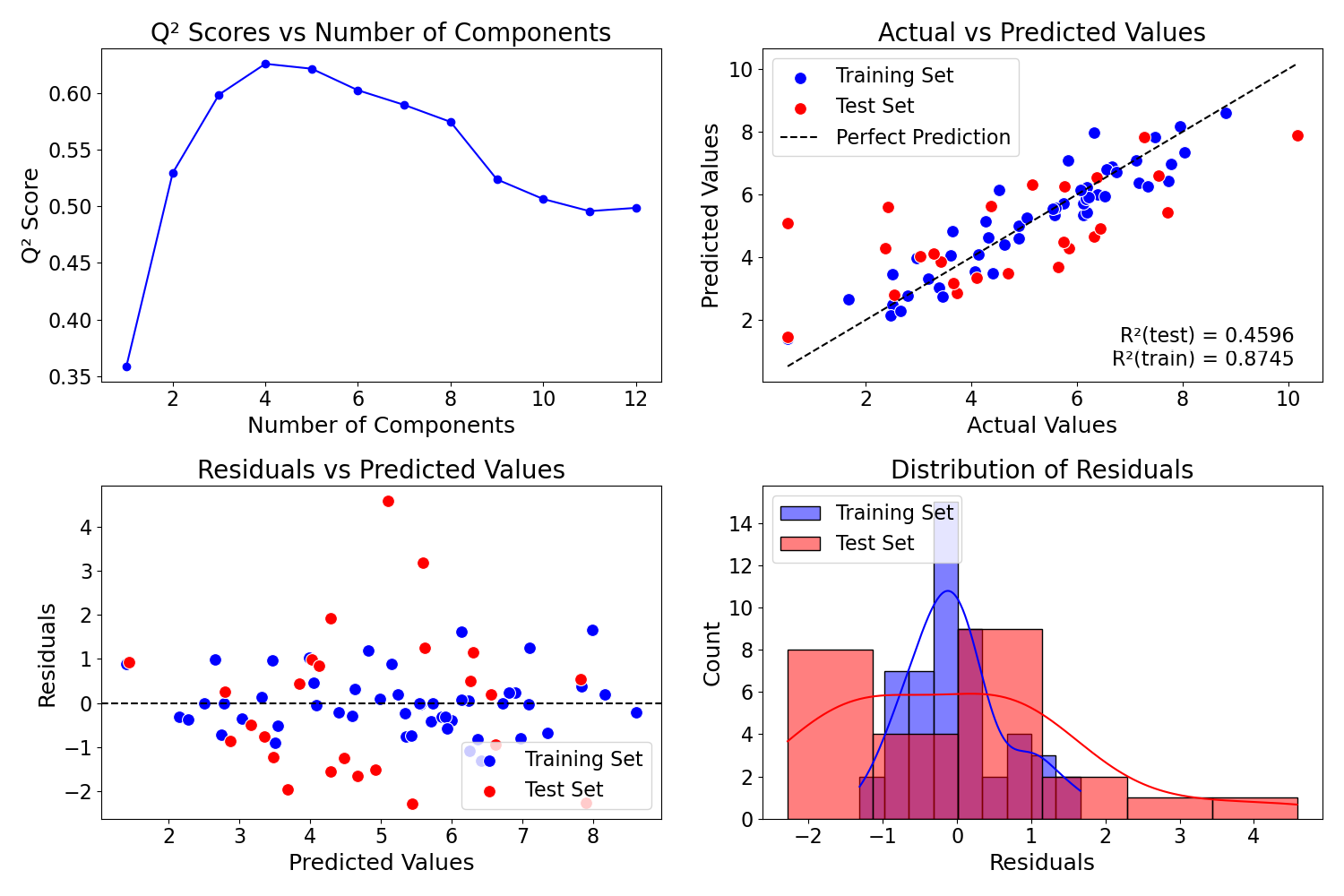

Supplement: Supplementary file 1 [file pharmaceuticals-18-00440-s001.zip › File S1/THERM_SEAD_2025-02-21_12-03-41/PLS_Analysis/PLSplots.png]

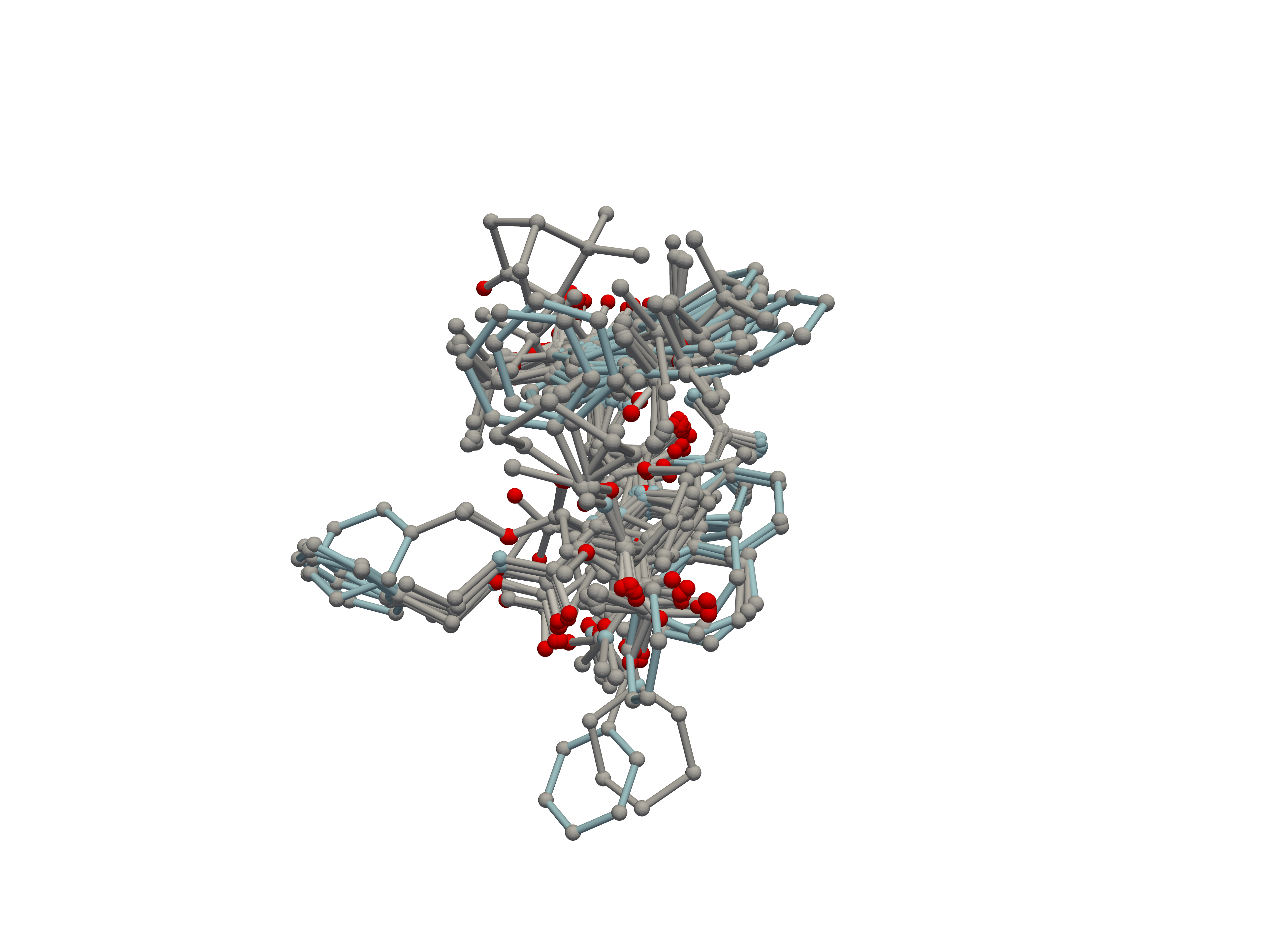

Supplement: Supplementary file 1 [file pharmaceuticals-18-00440-s001.zip › File S1/THR_all_2025-02-21_11-46-58/Alignments/aligned_molecules.png]

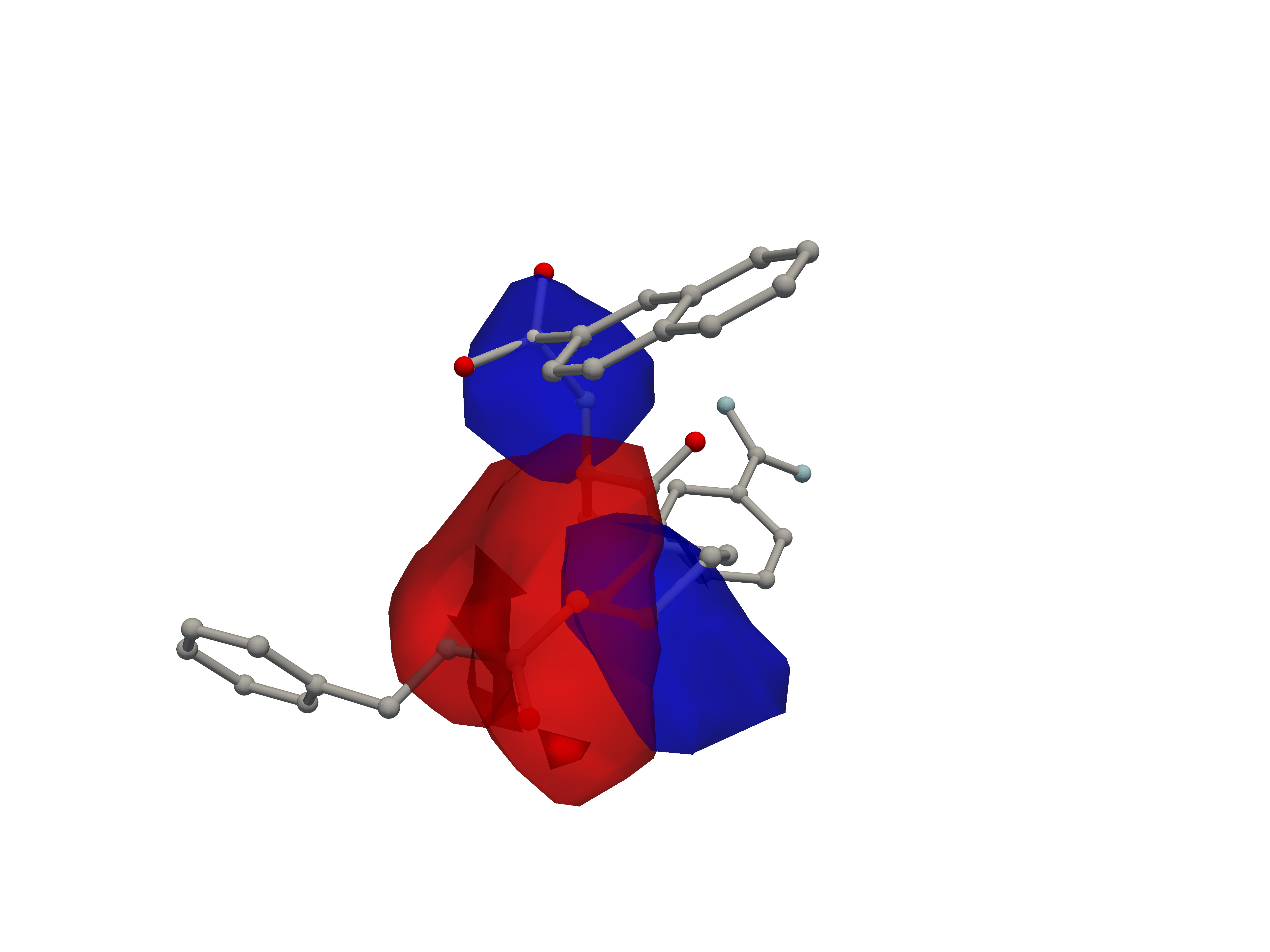

Supplement: Supplementary file 1 [file pharmaceuticals-18-00440-s001.zip › File S1/THR_all_2025-02-21_11-46-58/Contour_Plots/electrostatic_field_contourplot.png]

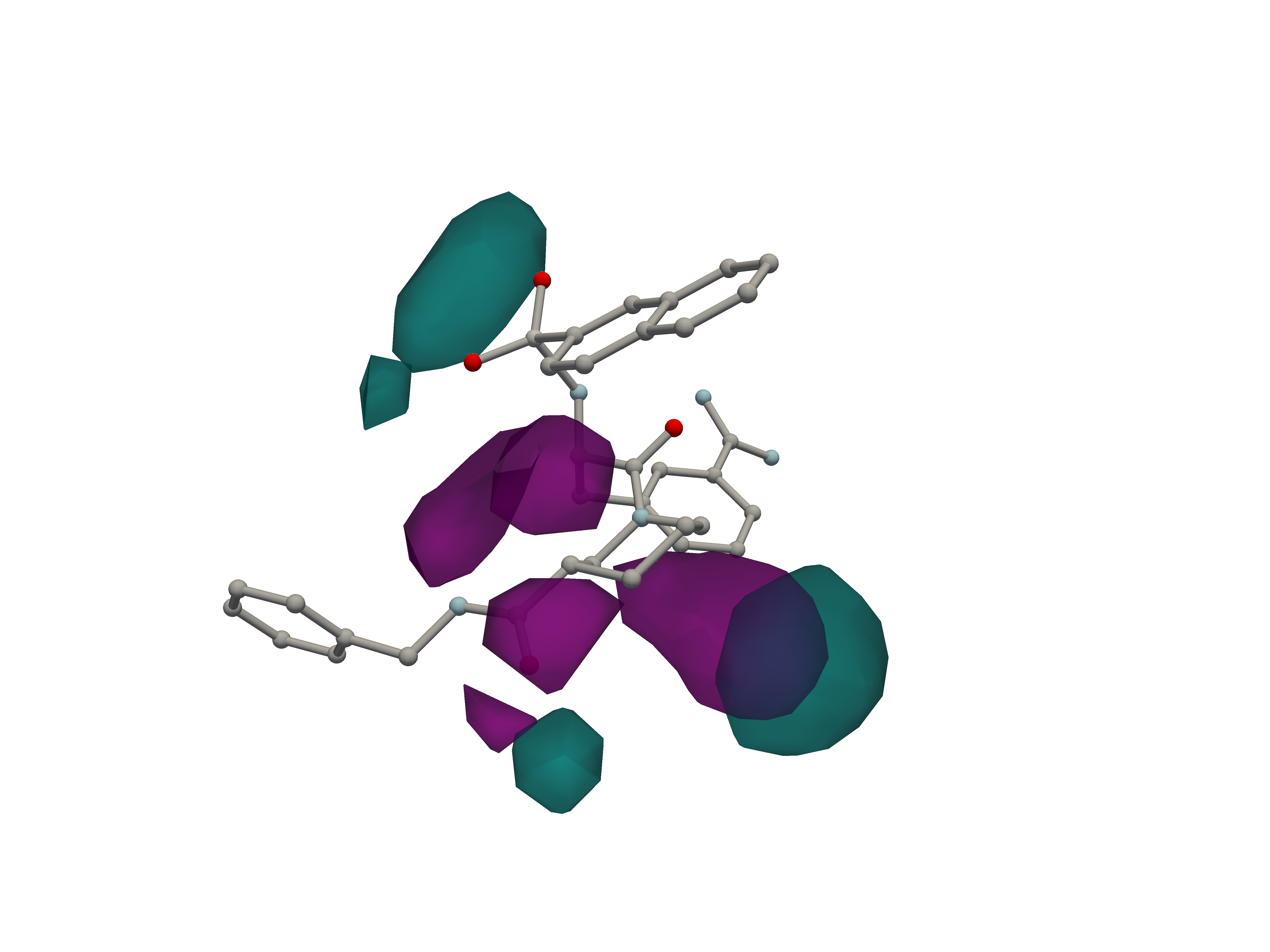

Supplement: Supplementary file 1 [file pharmaceuticals-18-00440-s001.zip › File S1/THR_all_2025-02-21_11-46-58/Contour_Plots/hbond_acceptor_field_contourplot.png]

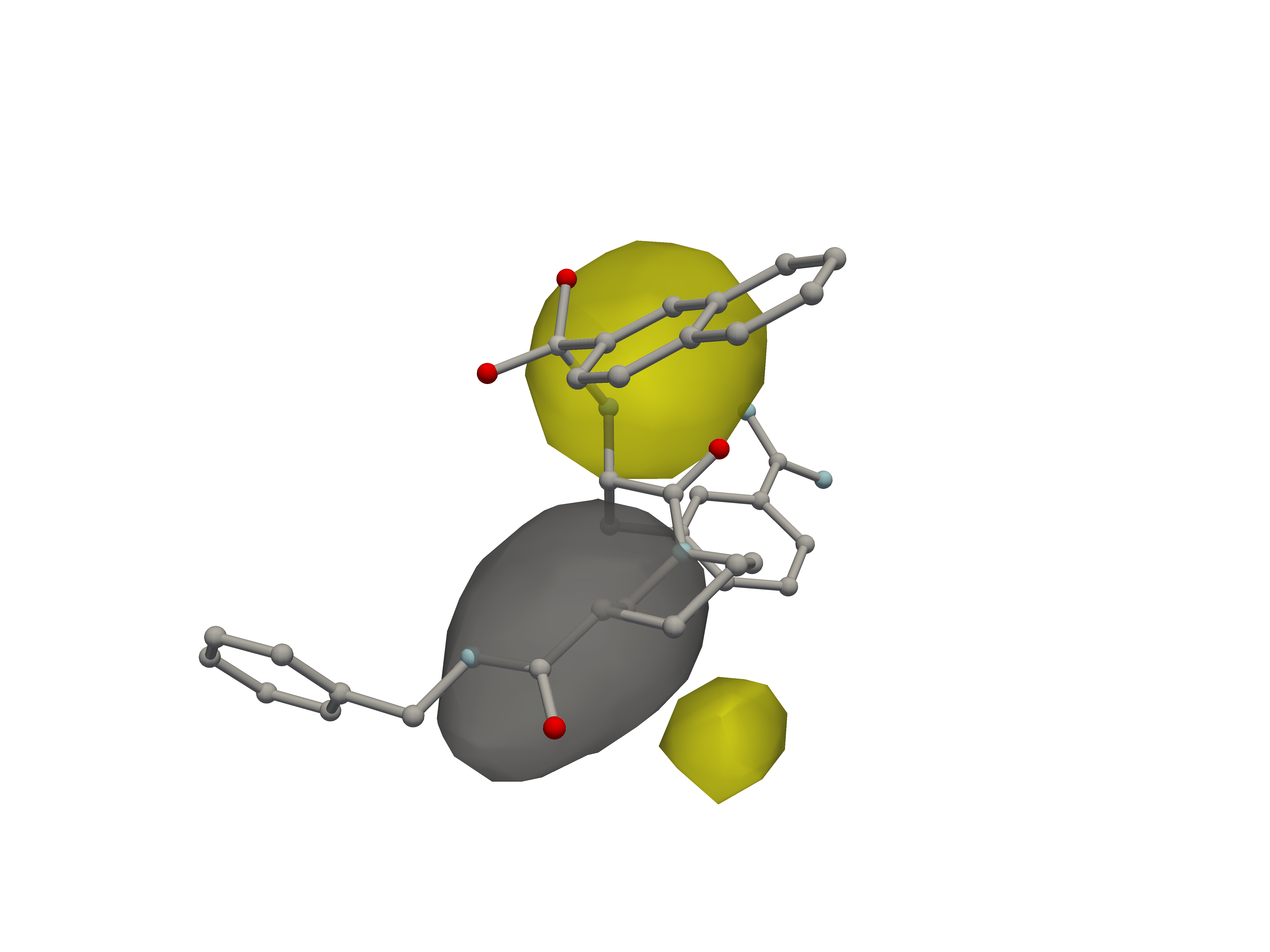

Supplement: Supplementary file 1 [file pharmaceuticals-18-00440-s001.zip › File S1/THR_all_2025-02-21_11-46-58/Contour_Plots/hbond_donor_field_contourplot.png]

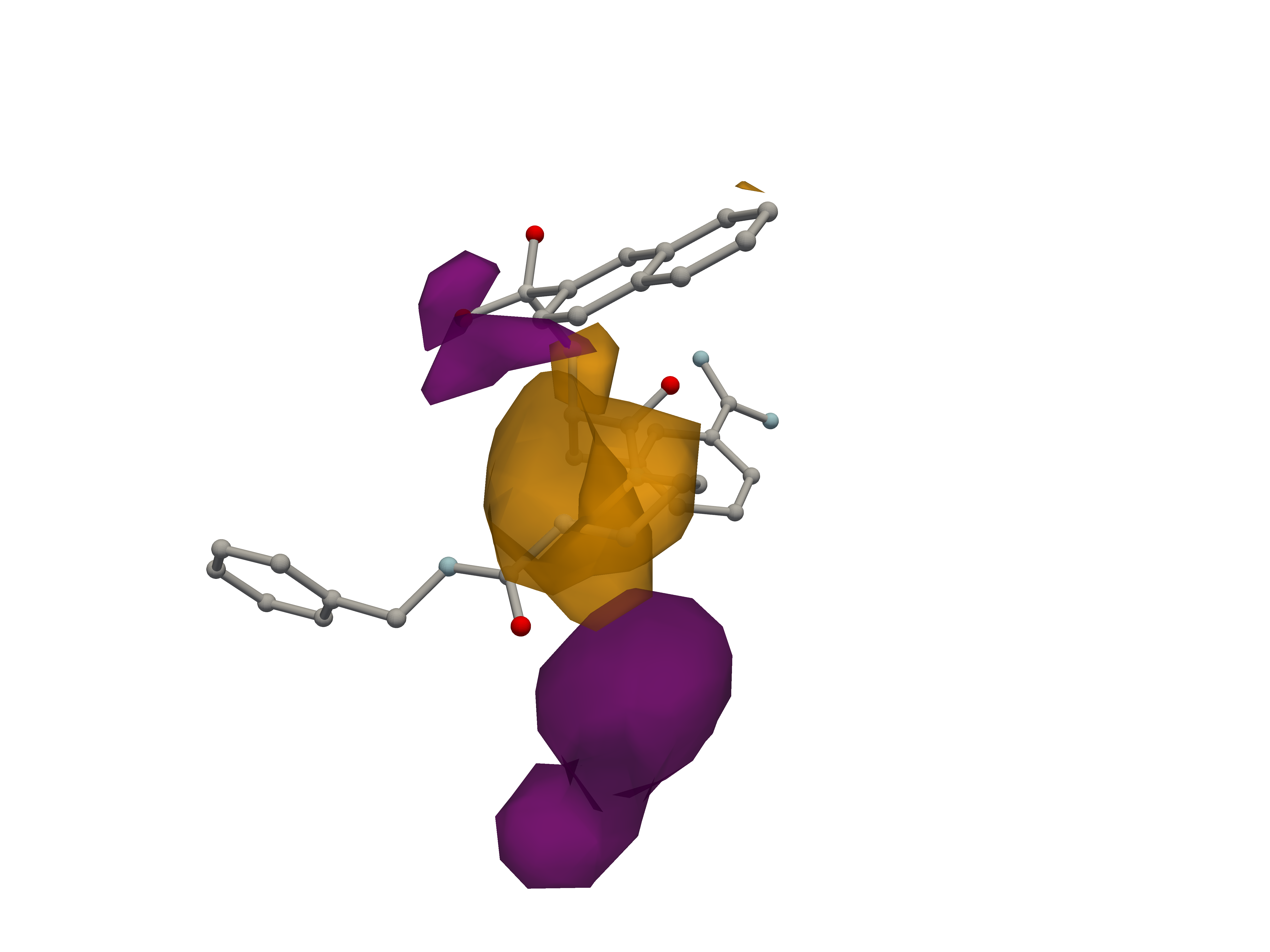

Supplement: Supplementary file 1 [file pharmaceuticals-18-00440-s001.zip › File S1/THR_all_2025-02-21_11-46-58/Contour_Plots/hydrophobic_field_contourplot.png]

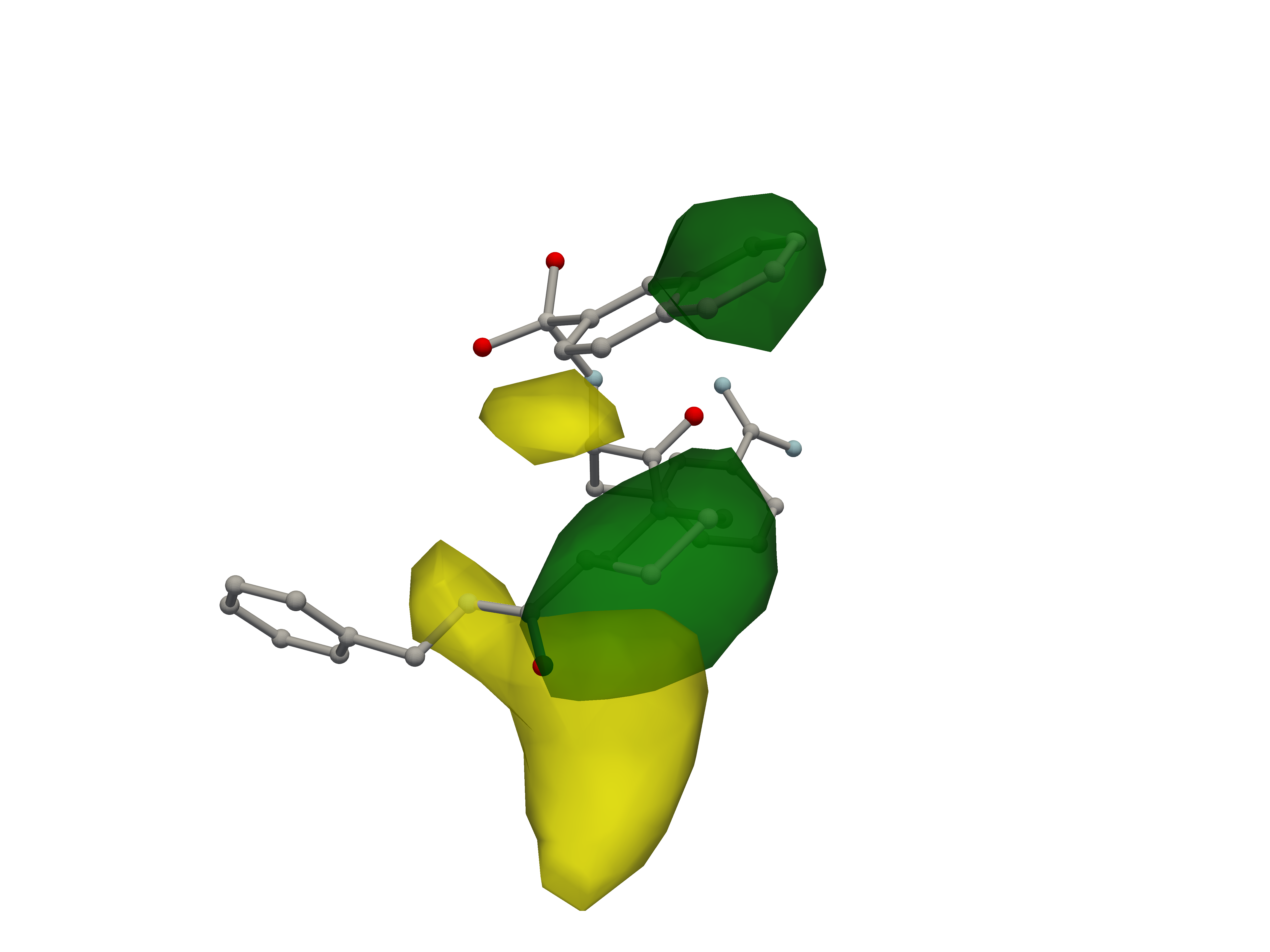

Supplement: Supplementary file 1 [file pharmaceuticals-18-00440-s001.zip › File S1/THR_all_2025-02-21_11-46-58/Contour_Plots/steric_field_contourplot.png]

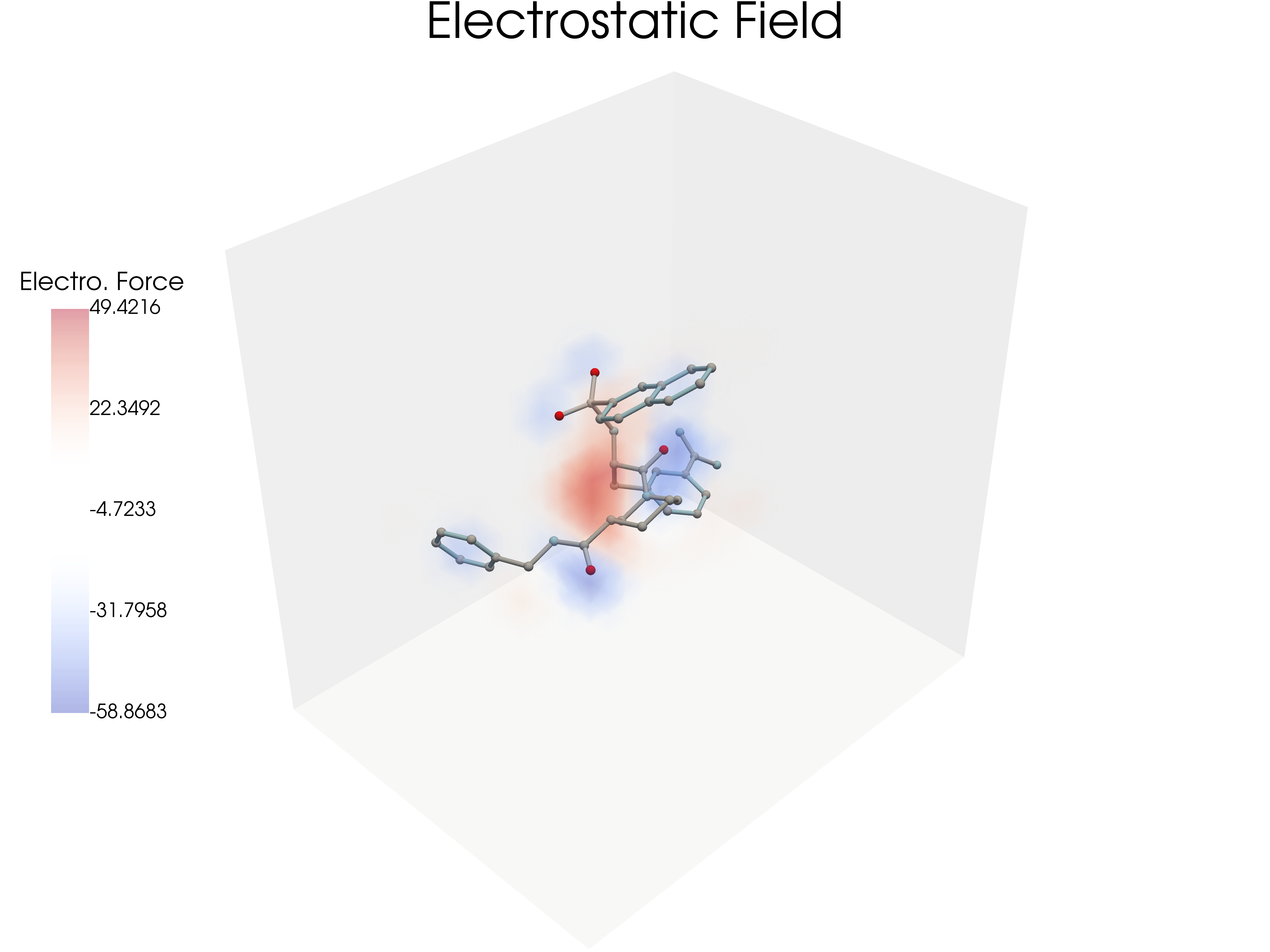

Supplement: Supplementary file 1 [file pharmaceuticals-18-00440-s001.zip › File S1/THR_all_2025-02-21_11-46-58/Field_Plots/electrostatic.png]

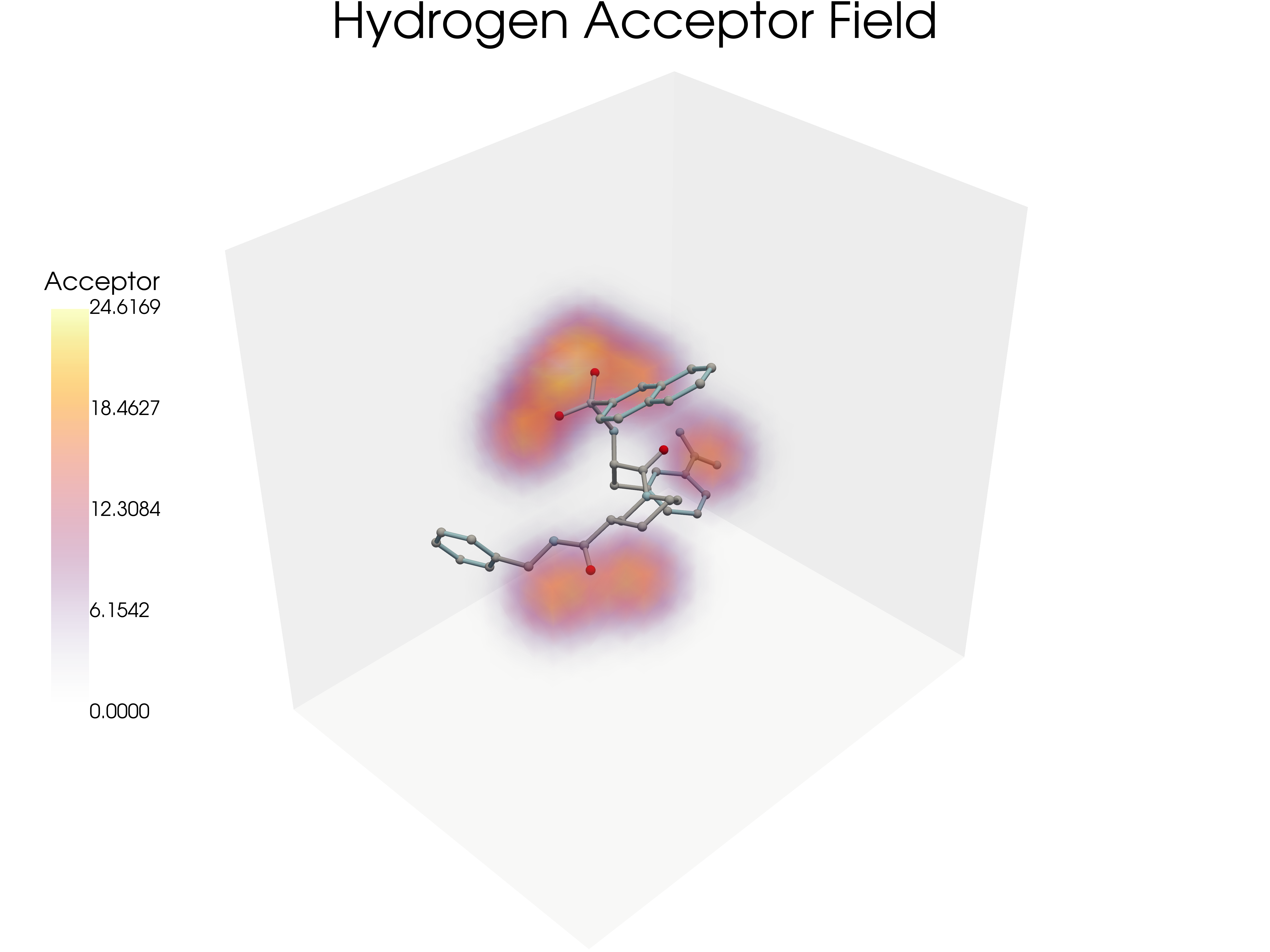

Supplement: Supplementary file 1 [file pharmaceuticals-18-00440-s001.zip › File S1/THR_all_2025-02-21_11-46-58/Field_Plots/hbond_acceptor.png]

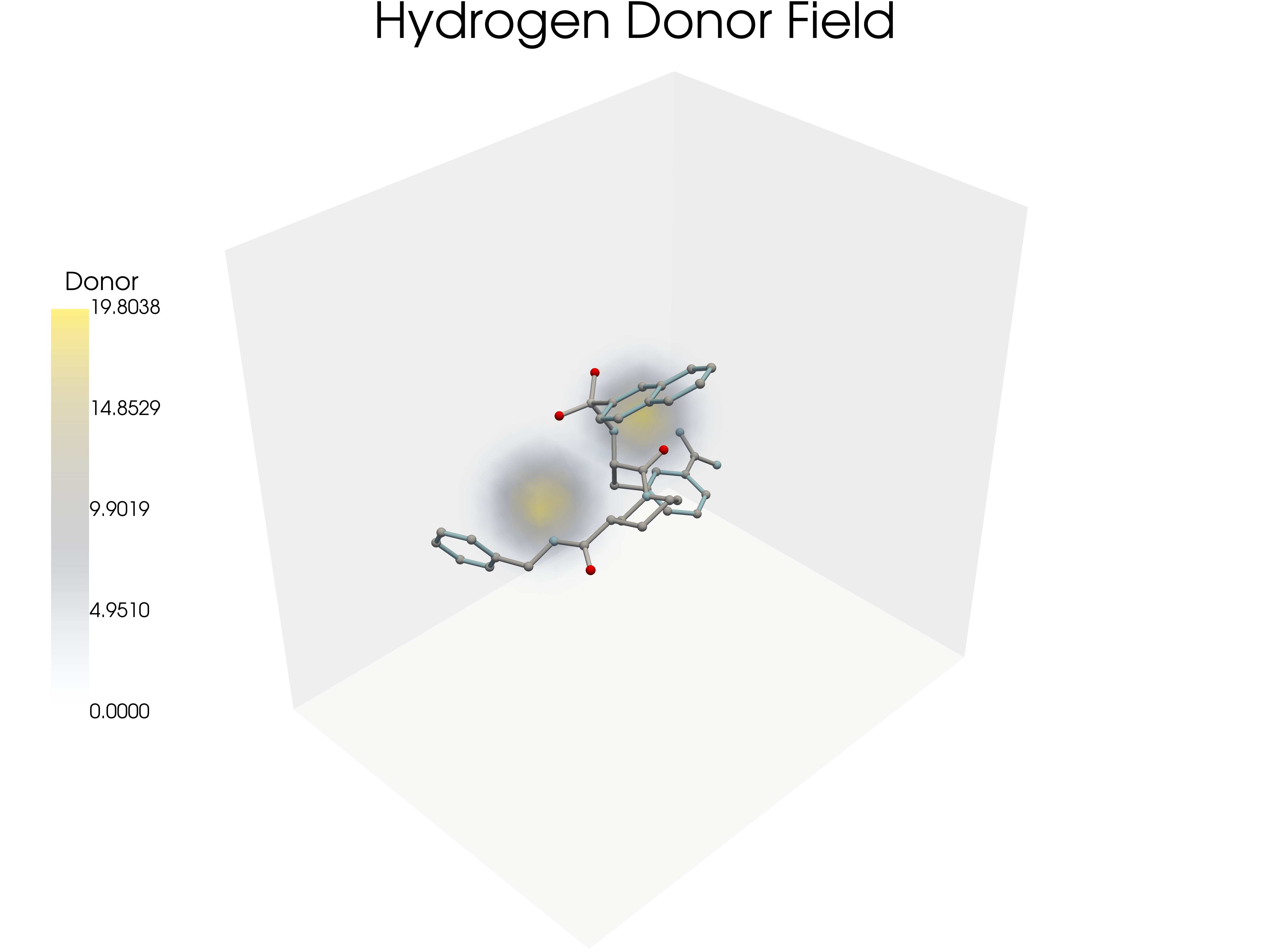

Supplement: Supplementary file 1 [file pharmaceuticals-18-00440-s001.zip › File S1/THR_all_2025-02-21_11-46-58/Field_Plots/hbond_donor.png]

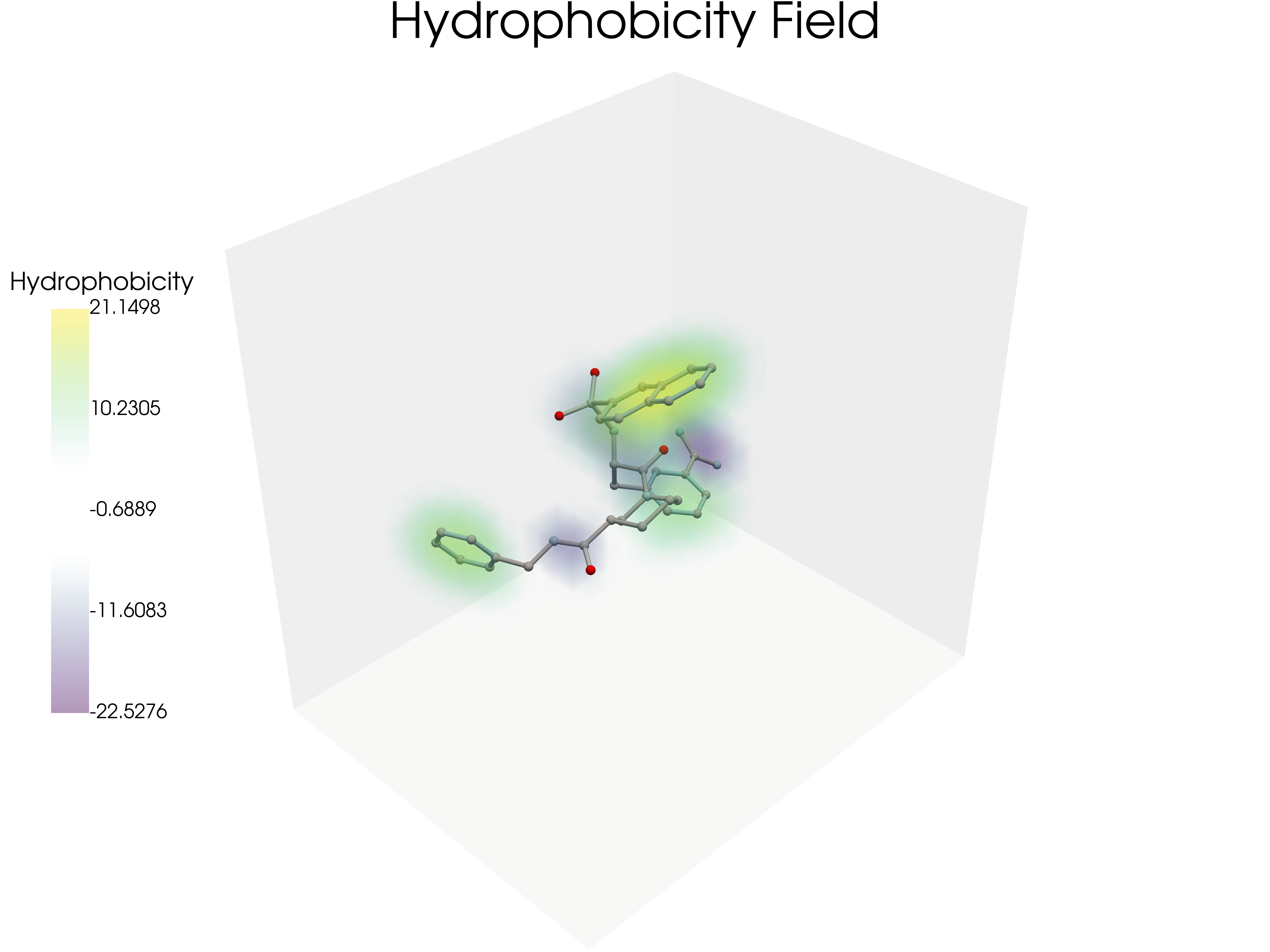

Supplement: Supplementary file 1 [file pharmaceuticals-18-00440-s001.zip › File S1/THR_all_2025-02-21_11-46-58/Field_Plots/hydrophobic.png]

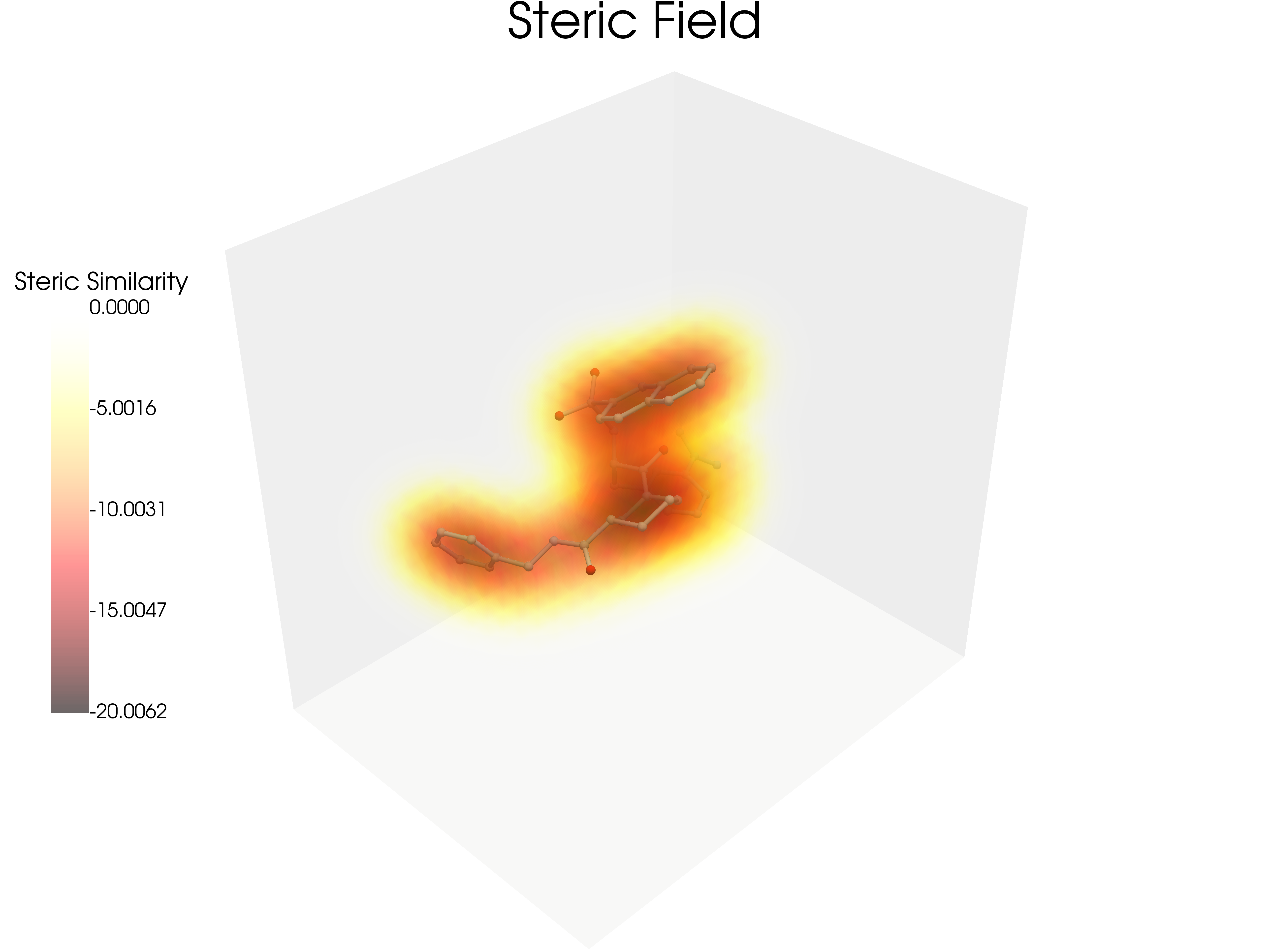

Supplement: Supplementary file 1 [file pharmaceuticals-18-00440-s001.zip › File S1/THR_all_2025-02-21_11-46-58/Field_Plots/steric.png]

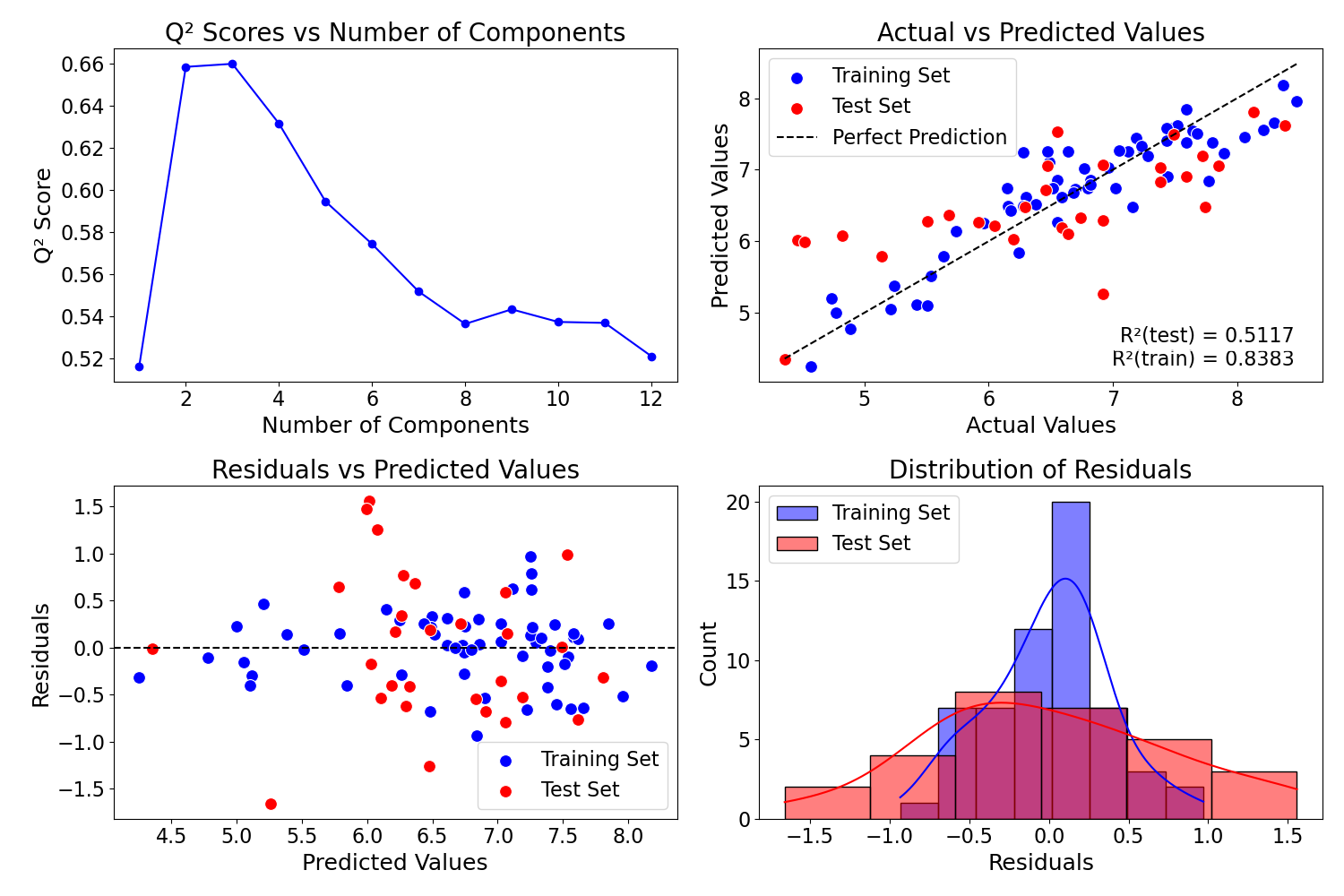

Supplement: Supplementary file 1 [file pharmaceuticals-18-00440-s001.zip › File S1/THR_all_2025-02-21_11-46-58/PLS_Analysis/PLSplots.png]
